# Novel Thiazole-Based SIRT2 Inhibitors Discovered via Molecular Modelling Studies and Enzymatic Assays

**DOI:** 10.3390/ph16091316

**Published:** 2023-09-18

**Authors:** Elena Abbotto, Beatrice Casini, Francesco Piacente, Naomi Scarano, Elena Cerri, Michele Tonelli, Cecilia Astigiano, Enrico Millo, Laura Sturla, Santina Bruzzone, Elena Cichero

**Affiliations:** 1 Department of Experimental Medicine, Section of Biochemistry, University of Genoa, Viale Benedetto XV 1, 16132 Genoa, Italy; elena.abbotto@unige.it (E.A.); francesco.piacente@unige.it (F.P.); elena.cerri.98@gmail.com (E.C.); cecilia.astigiano@edu.unige.it (C.A.); enrico.millo@unige.it (E.M.); laurasturla@unige.it (L.S.); 2 Department of Pharmacy, Section of Medicinal Chemistry, School of Medical and Pharmaceutical Sciences, University of Genoa, Viale Benedetto XV, 3, 16132 Genoa, Italy; beatrice.casini.bc@gmail.com (B.C.); naomi.scarano@edu.unige.it (N.S.); michele.tonelli@unige.it (M.T.); 3 IRCCS Ospedale Policlinico San Martino, Largo Rosanna Benzi 10, 16132 Genova, Italy

**Keywords:** SIRT2, sirtuin, virtual screening, molecular docking, enzymatic assays, inhibitor, HSP70

## Abstract

Recently, the development of sirtuin small molecule inhibitors (SIRTIs) has been gaining attention for the treatment of different cancer types, but also to contrast neurodegenerative disease, diabetes, and autoimmune syndromes. In the search for SIRT2 modulators, the availability of several X-crystallographic data regarding SIRT2−ligand complexes has allowed for setting up a structure-based study, which is herein presented. A set of 116 SIRT2 inhibitors featuring different chemical structures has been collected from the literature and used for molecular docking studies involving 4RMG and 5MAT PDB codes. The information found highlights key contacts with the SIRT2 binding pocket such as Van der Waals and π–π stacking with Tyr104, Phe119, Phe234, and Phe235 in order to achieve high inhibitory ability values. Following the preliminary virtual screening studies, a small in-house library of compounds (**1a**–**7a**), previously investigated as putative HSP70 inhibitors, was described to guide the search for dual-acting HSP70/SIRT2 inhibitors. Biological and enzymatic assays validated the whole procedure. Compounds **2a** and **7a** were found to be the most promising derivatives herein proposed.

## 1. Introduction

Sirtuins (SIRTs) are classified as class III histone deacetylases (HDACs), a family of enzymes that catalyze the removal of acetyl groups from ε-N-acetyl lysine amino acids of histone proteins, counteracting the activity played by histone acetyltransferases (HATs) [1].

Dysregulation involving HDAC and HAT activities results in different disorders such as neurodegenerative syndromes, metabolic diseases, and cancer, which are related to different SIRT functions [1,2,3,4].

Indeed, SIRTs include seven isoforms (SIRT1-7) endowed with different subcellular localizations and substrate specificities. While SIRT1, SIRT6, and SIRT7 are predominant as nuclear proteins, SIRT2 proteins are mostly cytoplasmatic and SIRT3-5 are found in the mitochondria [2,5].

Structurally, SIRT1-7 share a catalytic domain including approximately 270 amino acids, which are delimited by a Rossmann fold and a smaller cavity with the NAD^+^- and a zinc-binding site [6,7]. However, the N-terminal and C-terminal domains of SIRT1-7 are different. Based on the involvement of SIRTs in several biological pathways, such as transcription to metabolism and to genome stability, the dysregulation of SIRTs has been investigated in many diseases, including neurodegenerative disorders, cancer, diabetes, and cardiovascular and autoimmune diseases. In particular, extensive research has already been conducted to identify SIRT1 and, more recently, SIRT2 modulators as well [8,9].

SIRT2 is mainly expressed in the central nervous system (CNS) and is overexpressed in neurological disease, where it seems to promote neurodegenerative events [10]. Accordingly, SIRT2 inhibition has been reported as being able to protect neurons from toxicity due to increased α-synuclein levels—a hallmark of Parkinson’s Disease [11]. Regarding Alzheimer’s Disease, SIRT2 inhibition has been shown to reduce beta-amyloid converting enzyme 1 (BACE1) expression and improve cognitive impairment in mouse models of the disease [12]. In addition, SIRT2 expression is down- or up-regulated in different malignancies, making SIRT2 modulators interesting compounds in the search for anti-cancer agents [2].

Notably, novel approaches for contrasting tumors are necessary not only as a result of increasing incidences of cancer worldwide, but also to overcome the problem of resistance versus conventional anti-cancer therapy. This has spurred different researchers around the world to develop sirtuin small molecule modulators, either inhibitors (SIRTIs) or activators, not only to treat different cancer types, but also to be exploited in neurodegeneration and related pathologies [13].

Computer-aided drug design approaches, such as ligand-based and structure-based methods, represent widely exploited tools to accelerate the discovery of novel bioactive compounds [14,15,16].

Un until now, several X-ray crystallographic data of SIRT2 have been available in the Protein Data Bank [17,18], regarding different protein conformations [19,20] in presence or not of the substrate or of enzyme inhibitors [21]. This large amount of data provide key information to set up structure-based studies towards new putative SIRT2-targeting compounds.

Indeed, efforts to explore SIRT2 selectivity were achieved in 2015 through the publication of the first X-ray crystallographic structure of the protein in complex with the potent and selective inhibitor SirReal2 (PDB code = 4RMG) [22]. Subsequent studies led to further different experimental data regarding SIRT2 in the presence of further different chemo-types as SIRT2 inhibitors [23].

Herein, structure-based virtual screening (SBVS) studies have been developed with the aim of gaining more information about the putative binding mode of different series of effective SIRT2 inhibitors. In particular, SirReal2-like compounds and non-SirReal2-based SIRT2 inhibitors have been taken into account based on the available data reported in the literature [24,25,26]. This information allowed us to deeply explore the aforementioned series of SIRT2 inhibitors in silico, featuring variable rigid or flexible moieties tethered to the main core.

The results have been used to preliminary evaluate a small library of in-house compounds via molecular docking, including thiazole-based derivatives, previously developed as Heat Shock Protein 70 (HSP70) inhibitors [27]. The results obtained in our study allowed for the identification of a number of putative SIRT2 inhibitors, based on their ability to mimic the docking positioning previously observed by known SIRT2 inhibitors.

The subsequent in vitro evaluation confirmed the effectiveness of the proposed compounds **1a**–**7a** as novel SIRT2 inhibitors (SIRT2 inhibition at 150 μM = 76–100%), with **2a**, **6a**, and **7a** presenting the highest inhibition among the tested compounds (SIRT2 inhibition at 150 μM > 90%). Finally, the entire study has been accompanied by in silico prediction of descriptors related to the absorption, distribution, metabolism, and excretion properties (ADME) of **1a**–**7a**, in order to better prioritize the newly discovered SIRT2 inhibitors for further optimization.

## 2. Results

Considering the structural information of the SIRT2−ligand complexes, collecting and exploring the binding mode of different chemo-types as SIRT2 inhibitors is expected to highlight more information about their target flexibility and behavior in the presence of different modulators.

With this aim, herein, we explored the X-ray data of SirReal2 (PDB code = 4RMG) [22] and of a non-SirReal2 inhibitor (PDB code = 5MAT) [25], as downloaded from the Protein Data Bank [17,18]. Both of them were analyzed using the Protein–Ligand Interaction Profiler website (PLIP) [28,29], revealing hydrophobic contacts and π–π stacking as the driving force stabilizing the enzyme−ligand complex.

As shown in Figure S1, SirReal2 efficiently bound SIRT2 via hydrophobic interactions involving the terminal pyrimidine and naphthyl groups and Tyr139, Phe143, Ile169, and Phe119, Phe131, Ile232, Phe234, respectively. Similarly, the same contacts were also experienced by the 5MAT co-crystallized ligand thanks to the naphthyl group and the terminal oxazole ring (Figure S2).

Based on this, we proceeded with molecular docking studies on a dataset of SIRT2 inhibitors collected from the literature (see the chemical structure in Tables S1–S3) [24,25,26], either maintaining or not maintaining the chemical core included in 4RMG or 5MAT.

The obtained results were expected to (i) highlight the most relevant key contacts to stabilize the protein−ligand complexes and (ii) suggest useful hints for the subsequent virtual screening (VS) of the in-house library of thiazole/thiazolinone-based compounds (**1a**–**7a**), which were previously explored as HSP70 inhibitors.

In particular, to assess the most predictive molecular docking protocol to be used for the aforementioned molecular docking studies and VS calculations, deep re-cross docking simulations involving the 4RMG and 5MAT co-crystallized inhibitors were performed, based on a procedure already described in the literature [30,31,32,33].

Two series of re-docking calculations were applied via LeadIT [34] and MOE Dock [35] (details in Section 4).

The top five best scoring docking positions of the SIRT2 co-crystallized inhibitors docked at the 4RMG and 5MAT PDB codes are shown in Table S4. Similar binding modes were calculated for all of the compounds, resulting in recurrent conformer clusters, especially in the case of MOE docking simulations. Indeed, the MOE Dock module was able to suggest more comparable docking poses compared with than the LeadIT simulation when considering the experimental X-ray crystallographic data, providing lower (root mean square deviation values) RMSD values with respect to the experimental positioning. As shown in Figure S3, SirReal2 and the thienopyrimidinone derivative included in 5MAT were more efficiently predicted by MOE, featuring RMSD spanning from 0.520 to 1.380 Å.

Then, comparing 4RMG and 5MAT in terms of protein flexibility, the corresponding RMSD values, evaluated with respect to the alpha carbon atoms (CA atoms) and to the whole protein structure, moved from 2.220 to 2.597 Å (see Figure S4).

This piece of information revealed structural differences in the positioning featured by the residues of the SIRT2 binding site, when in presence of different chemo-types, via induced-fit events. Notably, this kind of result was recently described by us when comparing eight X-ray crystallographic data of SIRT2 [23]. Conversely, the protein domains outside the inhibitor binding cavity were properly superposed, as shown by the alignment and superimposition of the two PDB codes in Figure S5.

**Figure 1:**
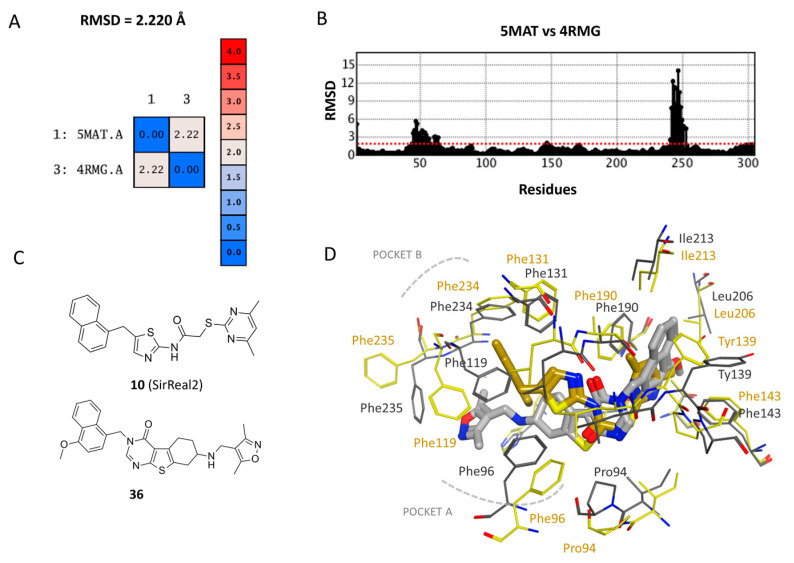
RMSD values as obtained by the superimposition of 4RMG and 5MAT are shown based on the carbon atom alignment (**A**) and the overall RMSD variation trend (**B**). Maximal allowed RMSD variation is reported as red dotted line. Chemical structure of the corresponding co-crystallized inhibitors (**10** in RMG and **36** in 5MAT) are reported (**C**). Superimposition of the 4RMG (in gold) and 5MAT (in grey) PDB codes in the presence of the co-crystallized ligands is also depicted (**D**).

As reported in Figure 1, the main flexible portion of the SIRT2 enzyme includes amino acids 50–120 and 220–280 from the protein primary sequence, which are within proximity of the ligand binding site.

Among them, Pro94, Phe96, Phe119, and Phe235 switched their orientation from the 5MAT to the 4RMG crystallographic data, because of the different dimensions featured by the related co-crystallized inhibitors. Indeed, the main tricyclic ring of the 5MAT ligand extended towards Pro94, Phe96, and Leu134 (pocket A), while the SirReal2 naphthyl group was surrounded by Phe119, Phe234, and Phe235 (pocket B). As a consequence, the SirReal2 naphthyl group and the terminal oxazole ring of the thienopyrimidinone derivatives detected π–π stacking with Phe234 and Phe119, respectively.

On the contrary, the SIRT2 Phe143, Phe190, Leu206, and Ile213 residues were rather stable and maintained comparable conformer positioning between the 4RMG and 5MAT experimental data. In any case, these amino acids were found to be involved in interactions with the co-crystallized inhibitor pyrimidine and naphthyl groups, respectively. Notably, these substituents of the two inhibitors were perfectly superposed, suggesting that binding to the hydrophobic residues Phe143, Phe190, Leu206, and Ile213 plays a key role in efficiently stabilizing the enzyme modulator. On the other hand, the possibility of interaction via Van der Waals contacts or π–π stacking with Phe131, Phe234, Phe96, or Phe119 is expected to contribute to the inhibitor efficiency.

To better investigate this aspect, molecular docking studies involving other known SIRT2 inhibitors were performed. The results of the molecular docking simulations are described as follows.

### 2.1. Molecular Docking Studies of SIRT2 Inhibitors

Molecular docking studies of a large dataset of SIRT2 inhibitors were performed, relying on the X-ray experimental data of SirReal2 (PDB code = 4RMG) [22], taken as representative of the selective and flexible SIRT2 inhibitors, and of a thienopyrimidinone-based compound (PDB code = 5MAT) [25], which was selected as a rigid and bulky reference inhibitor.

Approximately 100 known SIRT2 inhibitors (**1**–**116**) have been collected from the literature [24,25,26] and have been built in silico (see the chemical structure in Tables S1–S3).

**Figure 2:**
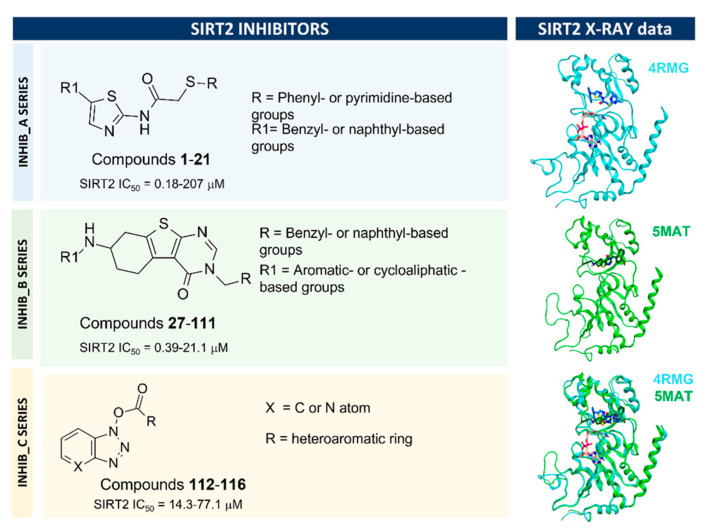
Scheme of the most representative compounds belonging to the main series of SIRT2 inhibitors herein investigated. The exploited SIRT2 X-ray data for the corresponding molecular docking studies are also shown.

Among them, compounds **1**–**26** (series A) feature a SirReal2-like molecular scaffold, whereas compounds **27**–**111** (series B) share a similar chemo-type with respect to the 5MAT co-crystallized ligand (See Figure 2). Finally, SIRT2 inhibitors **112**–**116** (series C) exhibit a different and rather rigid chemical structure (See Figure 2).

The corresponding potency trend of the SIRT2 inhibitors (IC_50_ values) for series A, B, and C, was determined via a fluorescence-based homogeneous assay using the substrate ZMAL (Cbz-Lys(acetyl)-AMC) [24], through enzymatic assays using the fluorogenic peptide substrate from p53 residues 379–382 RHKK(Ac)-AMC for SIRT2 [25,36] and via HPLC assay through a deacetylation reaction of an acetylated histone H3K9 peptide KQTAR(AcK)STGGKAWW (H3K9Ac) taken as a substrate for SIRT2 [26].

While the series A and series B compounds were docked at the 4RMG and 5MAT PDB codes based on their structural similarity with the co-crystallized ligand (see the calculated scoring functions in Tables S5 and S6), those belonging to series C were explored for both of the SIRT2−ligand complexes (PDB codes = 4RMG and 5MAT; the corresponding best ranked poses in tandem with the calculated scoring functions are shown in Tables S7 and S8).

For most of the compounds of the three series of inhibitors A–C, the applied MOE dock scoring function S (ΔG) was able to properly rank the most active derivatives with respect to the less potent ones. Regarding the compounds in series A, featuring a variable potency trend (IC_50_ = 0.18–502.80 μM), the observed S values ranged from −6.5829 to 11.7580 kcal/mol. The best ranked docking poses for all of the most potent inhibitors in series A (**10**, **11**, **13**–**16** IC_50_ < 1 μM) were endowed with S values ≤ 10.000 kcal/mol. Except for compound **8**, all of the best ranked poses for the less potent inhibitors belonging to series A (**6**, **8**, **22**, **26**; IC_50_ > 167 μM) showed higher S values, spanning from −6.5829 to −8.4188 kcal/mol. The compounds in series B (IC_50_ = 0.39–21.10 μM) displayed S values spanning from −11.26263 to −14.6185 kcal/mol, and the most interesting derivatives (**36**, **49**, **56**, **62**, **98** IC_50_ < 1 μM), were accompanied by S values from −12.7933 to −14.1830 kcal/mol. The SIRT2 inhibitors of series C (IC_50_ = 14.3–77.1 μM) included a limited number of analogues, exhibiting a comparable potency trend. In this case, the corresponding S values ranged from −6.3165 to −8.1909 kcal/mol, with compound **114** being the most promising (IC_50_ = 14.3 μM) with the best ranked pose having an S value of −7.6047 kcal/mol.

**Figure 3:**
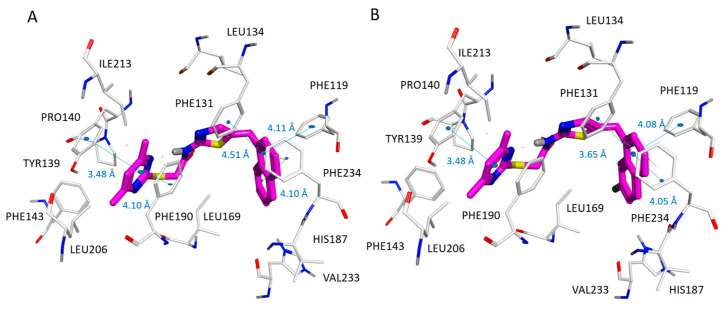
Docking positioning of SirReal2 (**10**; C atom, magenta) (**A**) and of the analogue **11** (C atom; magenta) (**B**) at the 4RMG binding site. Most of the potential π–π stacking contacts are shown in light blue and the corresponding distances in Å.

According to our docking positioning, the best ranked pose of SirReal2 (**10**) highly mimicked the bioactive conformer experienced by the co-crystallized ligand in 4RMG. As shown in Figure S6, the docked **10** was involved in a water-mediated contact with Pro94, while the dimethyl-substituted pyrimidine ring efficiently occupied the protein cavity delimited by the aromatic residues Tyr139, Phe143, and Phe190, displaying π–π stacking. Then, the naphthyl group of reference compound **10** was properly folded in proximity to Ile118, Leu134, Ile232, and Phe119, Phe131, and Phe234, featuring Van der Waal contacts and π–π stacking, respectively (see Figure 3A).

The main thiazole ring was able to detect hydrophobic contacts with the neighboring Ile169, Leu134, and Leu138.

Regarding the development of the SirReal2 (**10**; SIRT2 IC_50_ = 0.44 μM) analogues (**1**–**26**), the introduction of lipophilic atoms, such as the halogen atoms or methyl groups at the naphthyl substituent improved the SIRT2 inhibiting ability, as featured by compounds **11**, **14**, **15** (SIRT2 IC_50_ = 0.18–0.31 μM; see chemical structure in Table S1).

Indeed, the positioning of compound **11** docking suggested a key role played by the halogen atom to fit the enzyme pocket delimited by Ile169 for detecting Van der Waals contacts (see Figure 3B).

As a result, compared with SirReal2 (**10**), the compound **11** naphthyl substituent was also in closer proximity to Phe119, featuring π–π stacking and higher potency values.

**Figure 4:**
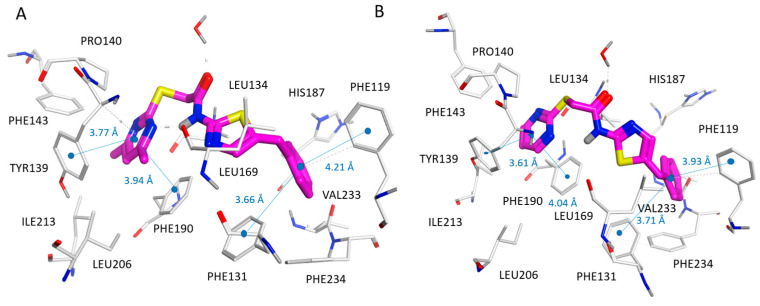
Docking positioning of compounds **1** (C atom, magenta) (**A**) and **2** (C atom; magenta) (**B**) at the 4RMG binding site. Most of the potential π–π stacking contacts are shown in light blue and the corresponding distances in Å.

The replacement of the naphthyl group of the prototype SirReal2 (**10**) with a (substituted)benzyl moiety (see compounds **1**–**5** in Table S1; SIRT2 IC_50_ = 1.33–16.8 μM), as well as the introduction of bulkier substituents at the terminal pyrimidine ring (see compounds **18**, **19**, **21** in Table S1;SIRT2 IC_50_ = 15–127.2 μM), or of totally unsubstituted pyrimidine (see compounds **2**, **17** in Table S1; SIRT2 IC_50_ = 2.34–16.8 μM), were detrimental to the SIRT2 inhibitor ability. Accordingly, SirReal2 analogues **1** and **2** exhibited comparable docking positioning with respect to the prototype, although at the expense of Van der Waals contacts and π–π stacking with the previously mentioned hydrophobic and aromatic residues (see Figure 4A,B).

Next, the replacement of the main thiazole core with a thiadiazole one led to SIRT2 inhibitors with a lower potency, as shown by compound **25** (see the chemical structure in Table S1; SIRT2 IC_50_ = 1.89 μM), compared with **10** (see the chemical structure in Table S1; SIRT2 IC_50_ = 0.44 μM), possibly because this more polar five-membered ring could impair the hydrophobic interactions with Ile169, Leu134, and Leu138. Accordingly, any thiadiazole-based analogue featuring the aforementioned detrimental structural variations at the pyrimidine and/or naphthyl groups also displayed a weak SIRT2 inhibiting ability (see compounds **22**, **24**, and **26**; SIRT2 IC_50_ = 30.9–502.8 μM).

The molecular docking positioning for compounds **25** and **23** is reported in Figure S7.

Exploring the SIRT2 inhibitors of series B via molecular docking studies allowed us to probe the effectiveness of a non-SirReal2-like structure, featuring bulkier and less flexible moieties than the previous prototype.

**Figure 5:**
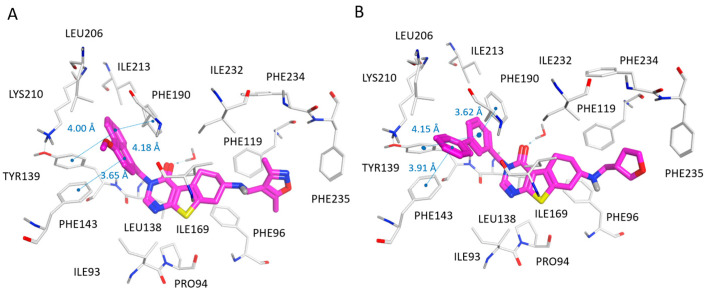
Docking positioning of compounds **36** (C atom, magenta) (**A**) and **94** (C atom; magenta) (**B**) at the 5MAT binding site. Most of the potential π–π stacking contacts are shown in light blue and the corresponding distances in Å.

Based on the PDB code 5MAT, the re-docking pose of the co-crystallized thienopyrimidinone derivative (**36**; SIRT2 IC_50_ = 0.58 μM) displayed π–π stacking involving the terminal naphthyl substituent and the Tyr139, Phe143, Phe190, and Phe214 residues, also featuring van der Waals contacts with the surrounding Pro140, Leu206, and Leu213 (see Figure 5A).

On this basis, the replacement of the naphthyl substituent with the benzyl one impaired the SIRT2 inhibitory activity, as shown by **50**, **54**, and **86** (see the chemical structure in Table S2; SIRT2 IC_50_ = 1.65–2.24 μM) when compared with **53**, **55**, and **89** (see the chemical structure in Table S2; SIRT2 IC_50_ = 9.18–13.40 μM), respectively.

In addition, the 4-methoxy substituent at the naphthyl group was proven to stabilize the inhibitor at the enzyme cavity via water-mediated contacts with the Lys210 side chain (see Figure S8).

Similarly, the terminal heteroaromatic ring of compound **36** was projected in proximity of Phe219 and Phe235, and the oxazole core was also engaged in polar contacts with the surrounding water molecules. As a result, the main tricyclic scaffold was maintained within the protein binding site exhibiting the required flat and hydrophobic features to fit the narrow pocket delimited by Ile93, Leu138, Leu134, Leu169, and Ile232. According to these results, removing the 4-methoxy-naphthyl group in favor of (un)- or 2-methoxy-substituted naphthyl ones led to less potent derivatives. This information was supported by the higher IC_50_ values featured by **27**, **36**, **66**, **74**, and **90** (see the chemical structure in Tables S1 and S2; SIRT2 IC_50_ = 0.58–5.24 μM) when compared with **28**, **35**, **67**, **75**, and **91** (see the chemical structure in Tables S1 and S2; SIRT2 IC_50_ = 1.85–12.48 μM).

Interestingly, the introduction of a biaryl substituent instead of the naphthyl group was sometimes beneficial or advantageous, with respect to 2-methoxy-naphthyl substituted or quinoline-based analogues. Indeed, compounds **94**, **96**, and **102** (see the chemical structure in Table S2; SIRT2 IC_50_ = 1.74–3.74 μM) were more potent than **93**, **95**, and **101**, (see the chemical structure in Table S2; SIRT2 IC_50_ = 5.06–6.03 μM). As shown in Figure 5B, the biaryl substituent of the SIRT2 inhibitor **94** was oriented towards Lys210, gaining cation-π contacts thanks to the ε-amino group of this residue.

Regarding structural variation involving the oxazole terminal ring of the reference compound **36**, the replacement of the R1 substituent of the prototype with a furan-, pyrazole,- or thiophene-based group, led to SIRT2 inhibitors endowed with quite comparable IC_50_ values. Indeed, the five-membered ring compounds **27**–**30** (see the chemical structure in Table S2; SIRT2 IC_50_ = 4.16–11.68 μM), **31**–**33** (see the chemical structure in Table S2; SIRT2 IC_50_ = 4.20–7.43 μM), and **38**–**39** (see the chemical structure in Table S2; SIRT2 IC_50_ = 2.56–6.03 μM) featured a similar SIRT2 inhibitory potency trend compared to the analogues **34**–**37** (see the chemical structure in Table S2; SIRT2 IC_50_ = 0.58–6.94 μM). The introduction in the R1 group of an aromatic six-membered ring allowed for the development of benzyl-, phenyl-, pyridine-, or pyrimidine-based derivatives. Among them, the benzyl- **40**–**59** (see the chemical structure in Table S2; SIRT2 IC_50_ = 0.39–21.10 μM) and phenyl- **60**–**62** (see the chemical structure in Table S2; SIRT2 IC_50_ = 0.97–4.25 μM) were more potent than the pyridine- **63**–**83** (see the chemical structure in Table S1; SIRT2 IC_50_ = 1.20–10.31 μM) and pyrimidine- **84**–**85** (see the chemical structure in Table S2; SIRT2 IC_50_ = 5.30–6.45 μM)-based analogues and quite comparable with the five-membered ring series of ligands **27**–**39** (see the chemical structure in Table S2; SIRT2 IC_50_ = 0.58–11.68 μM).

**Figure 6:**
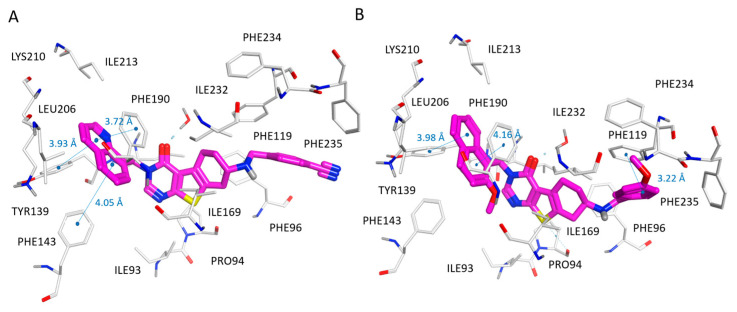
Docking positioning of compounds **49** (C atom, magenta) (**A**) and **56** (C atom; magenta) (**B**) at the 5MAT binding site. Most of the potential π–π stacking contacts are shown in light blue and the corresponding distances in Å.

As shown in Figure 6A, benzyl-based compound **49** (SIRT2 IC_50_ = 0.39 μM) highly mimicked the positioning featured by co-crystallized compound **36** (SIRT2 IC_50_ = 0.58 μM), maintaining the key contacts with the enzyme, for example through π–π stacking.

In addition, the p-CN-benzyl group in R1 of **49** guaranteed π–π stacking with Phe119 and much more polar contacts with the surrounding water molecules, compared with **36**, due to the solvent-exposed area of the protein (see Figure S9). This is thought to result in the higher IC_50_ values of **49** with respect to the prototype. Accordingly, analogue **56** (SIRT2 IC_50_ = 0.65 μM) exhibiting a p-OCH3-benzyl substituent in R1 was also able to display a comparable docking mode and similar potency as the SIRT2 inhibitor (Figure 6B).

**Figure 7:**
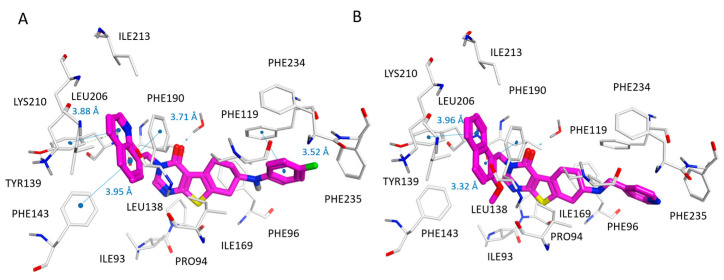
Docking positioning of compounds **62** (C atom, magenta) (**A**) and **66** (C atom; magenta) (**B**) at the 5MAT binding site. Most of the potential π–π stacking contacts are shown in light blue and the corresponding distances in Å.

A similar behavior could be observed within the phenyl-based series, as shown by the docking pose of compound **62** (SIRT2 IC_50_ = 0.97 μM) in Figure S10. In this pose, the p-F-phenyl group in R1 is orientated towards water molecules (solvent-exposed area of SIRT2) and Phe119, while maintaining hydrophobic and π–π stacking interactions (see Figure 7).

Regarding pyridine-based derivatives, similar to most potent derivatives featuring heteroaromatic six-membered ring, the 4-pyridine-substituted (**66**–**69**, **81**–**83**; SIRT2 IC_50_ = 1.20–9.60 μM) inhibitors were proven to be more promising than the 2- or 3-pyridine-substituted ones (**63**–**65**, **70**–**80**; SIRT2 IC_50_ = 1.45–10.31 μM), thanks to the most effective direction featured by the nitrogen atom with respect to the protein solvent-exposed and polar pocket. The positioning of **66** (see the chemical structure in Table S2; SIRT2 IC_50_ = 1.20 μM) is reported in Figure 7B, and is taken as being representative of the whole series (**63**–**83**; SIRT2 IC_50_ = 1.20–10.31 μM).

Finally, the choice of a small cycloaliphatic group in R1 in place of aromatic rings was more effective than the introduction of long-aliphatic chains tethering (hetero)cycloaliphatic groups or than condensed cycloaliphatic and aromatic moieties. Accordingly, the cyclopentyl-based compounds **97**–**99** (see the chemical structure in Table S2; SIRT2 IC_50_ = 0.73–1.52 μM) experienced higher IC_50_ values than the cyclohexyl-alkyl-based analogues **86**–**89** and **95**–**96** (see the chemical structure in Table S2; SIRT2 IC_50_ = 1.65–9.18 μM). Most of the compounds **97**–**99** were also more potent than the (hetero)cycloaliphatic derivatives **90**–**94** and **100**–**103** (see the chemical structure in Table S2; SIRT2 IC_50_ = 1.11–13.14 μM) and more than the condensed cycloaliphatic and aromatic-containing ones **105**–**107** (see the chemical structure in Table S2; SIRT2 IC_50_ = 3.09–16.32 μM).

Interestingly, structural simplification of the main tricyclic core featured by all of the previously mentioned thienopyrimidinone **36** analogues to the byciclic ring led to modest SIRT2 inhibitors **108**–**111** (see the chemical structure in Table S2; SIRT2 IC_50_ = 2.15–8.15 μM), if tethered to two terminal aromatic rings in R and R1. This information agrees with the higher potency values of **108**–**110** (SIRT2 IC_50_ = 2.15–3.36 μM) than that of **111** (SIRT2 IC_50_ = 8.15 μM). In Figure S11, the docking positioning of compounds **86** (see the chemical structure in Table S2; SIRT2 IC_50_ = 1.65 μM) and **90** (see the chemical structure in Table S2; SIRT2 IC_50_ = 1.90 μM), taken as being representative of the cycloaliphatic- and (hetero)cycloaliphatic-based inhibitor series, are reported.

Molecular docking studies performed on non-crystallized derivatives such as **112**–**116** (see the chemical structure in Table S3; SIRT2 IC_50_ = 14.3–77.1 μM) allowed for exploring further structural variations with respect to the previous ones and to deeply probe the role played by smaller and less flexible scaffolds in the place of those by compounds **10** (4RMG co-crystallized ligand) and **36** (5MAT co-crystallized thienopyrimidinone).

**Figure 8:**
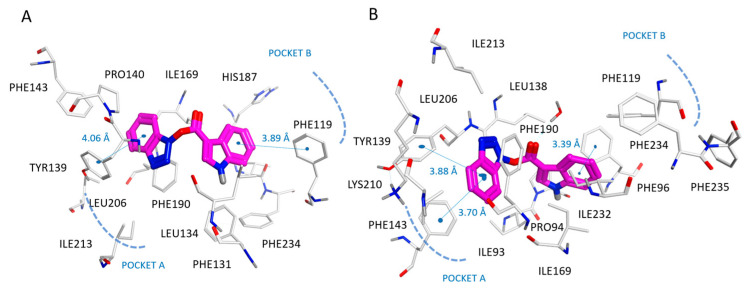
Docking positioning of compound **114** (C atom, magenta) at the 4RMG (**A**) and 5MAT PDB code (**B**) binding sites. Most of the potential π–π stacking contacts are shown in light blue and the corresponding distances in Å.

As shown in Figure 8, the carbonyl group and the benzotriazole ring of **114** highly mimicked the pyrimidine substituent and the pyrimidinone ring of the 5MAT co-crystallized ligand, detecting π–π stacking with Tyr139, Phe143, and Phe190 in any case.

On the contrary, the indole group of **114** only partially simulated the binding positioning featured by the naphthyl-substituted thiazole ring of **10** and that of the oxazole-containing thieno-pyrimidinone of **36**. As a consequence, **114** featured Van der Waals contacts with Pro94, Leu134, and π–π contacts with the surrounding Phe96 (pocket A). According to our calculations, the small dimension of **114** impaired the possibility for the ligand to fit the enzyme cavity properly, as it was not able to occupy the protein cavity delimited by Phe119, Phe234, and Phe235 (pocket B), as experienced by the R1 substituent of the two reference compounds (**10** and **36**). Accordingly, **114** (SIRT2 IC_50_ = 14.3 μM) displayed a lower potency as an SIRT2 inhibitor than **10** (SIRT2 IC_50_ = 0.44 μM) and **36** (SIRT2 IC_50_ = 0.58 μM).

As a result of all of the docking calculations, the development of optimized SirReal2 analogues could be achieved by improving hydrophobic contacts with Ile169, Leu134, and Leu138 (most of them belonging to pocket A) and π–π stacking with Phe119 (belonging to pocket B). On the contrary, the replacement of the main thiazole core or of the naphthyl pendants with more polar heteroaromatic rings could impair the inhibitory ability of the compound for SIRT2. In particular, the key role played by the naphthyl moiety was confirmed by series B of the SIRT2 inhibitors (see the prototype **36** structure). Regarding series B, the presence of H-bonding groups at the naphthyl group seemed to encourage stabilizing the inhibitor at the enzyme cavity, via water-mediated contacts with Lys210. On the other hand, the replacement of the **36** oxazole terminal ring with a further five- or six- membered rings was proven to be beneficial, especially in the case of furan-, pyrazole-, or thiophene-based groups. In addition, the choice of a small cycloaliphatic group such as the cyclopentyl group instead of the oxazole ring was advantageous.

Thus, it should be noted that the main features resulting in effective inhibitors for both series A–B for the docking calculations were related to properly target the SIRT2 cavity via interactions with aromatic and hydrophobic residues involving pockets A and B, as the inhibitor was also stabilized by polar contacts via water molecules and Pro94 or Lys210.

Concerning the SIRT2 inhibitors in series C, maintaining a small, planar, and electron-rich main moiety (such as compound **114**), the presence of two terminal aromatic substituents led to a folded ligand fitting the aforementioned minimum criteria needed to achieve SIRT2 inhibition. Then, high potency values were managed through the introduction of bulkier terminal groups to guarantee hydrophobic contacts with both pockets A and B.

### 2.2. Virtual Screening of in-House Libraries Compounds as SIRT2 Inhibitors

**Figure 9:**
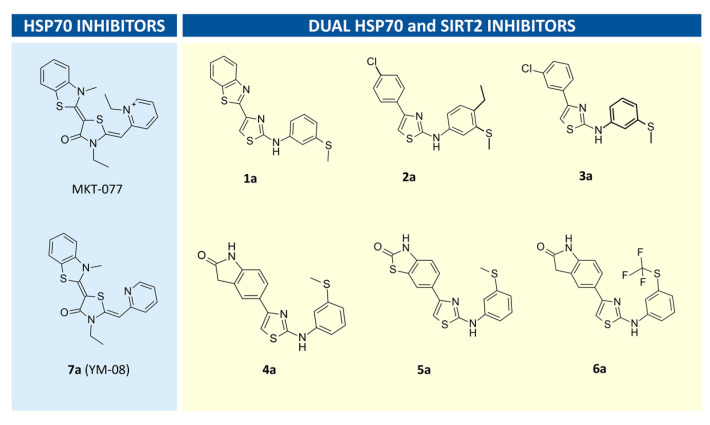
Chemical structure of the known HSP70 inhibitors MKT-077 and of the analogue YM-08 (**7a**) (**left**); series of screened in silico and in in vitro assays thiazole derivatives as dual inhibitors of HSP70 and SIRT2 (**right**).

In an attempt to find novel SIRT2 inhibitors, we performed a preliminary VS study to in silico explore the putative SIRT2-targeting ability featured by a small library of in-house compounds (**1a**–**6a**) (Figure 9A). The synthesis of **1a**–**6a** has been already reported by us [28,37,38], whereas compound **7a** (YM-08) was purchased, being commercially available as an HSP70 inhibitor [39].

Recently, we developed molecular modeling studies to explore putative HSP70 inhibitors, based on the structural similarity between **1a** and the well-known HSP70 ligand MKT-077 [27]. Indeed, HSP70 inhibition has been reported in the literature as being beneficial for the development of anti-cancer agents [40,41], and, more recently, as a modulator for the treatment of cystic fibrosis [27].

Compound **1a** resembled the chemical structure of the HSP70 inhibitors MKT-077 and YM-08, thanks to the central five-membered ring tethered to the bicyclic and phenyl rings, which was the in-house HSP70 inhibitor prototype. While the related analogues **4a**–**6a** maintained a terminal hydrophobic bicyclic substituent linked to the thiazole main core, **2a** and **3a** were derived via structural simplification of the bicyclic ring, exhibiting a p-Cl- or m-Cl phenyl group.

As shown in Figure 9, all of the compounds herein matched the specific requirement to also feature an SIRT2 inhibitory ability.

To investigate the behavior of compounds **1a**–**6a** as putative SIRT2 inhibitors, VS studies were performed through two runs of calculations using X-ray data from both 4RMG and 5MAT. In both cases, the protein binding site was maintained as a rigid (VS_RP; see the calculated scoring functions in Tables S9 and S10) or as flexible (VS_FP; the corresponding best ranked poses in tandem with the calculated scoring functions are shown in Tables S11 and S12) object during the calculations (details in Section 4), while the ligands were flexible.

To ascertain the reliability of the procedure so as to identify novel putative SIRT2 ligands, the known SIRT2 inhibitor AGK2 [42] was also evaluated via molecular docking calculations. Notably, this procedure was recently applied by us with success to identify novel scaffolds for the design of new SIRT2 inhibitors [23]. According to our results, all of the compounds occupied SIRT2 pockets A and B with the terminal aromatic ring. In particular, the bulkier substituent at positions 2 and 4 of the thiazole compound was projected towards pocket B, exhibiting π–π stacking with Phe119, Phe234, and Phe235, while the other aromatic group was oriented in pocket A, displaying van der Waals contacts with Leu206 and Ile 213, and π–π stacking with Tyr139 and Phe143. Notably, this kind of positioning was maintained at both PDB codes as VS_RP. The stability of the corresponding poses was then evaluated via VS_FP, providing comparable results and key contacts at the modelled SIRT2−ligand complexes.

**Figure 10:**
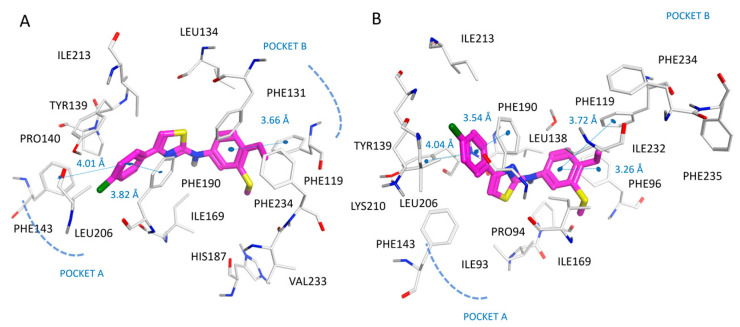
Docking positioning of compound **2a** (C atom, magenta) at the 4RMG (**A**) and at the 5MAT PDB code (**B**) binding site. Most of the potential π–π stacking contacts are shown in light blue and the corresponding distances in Å.

In particular, the docking mode featured by **2a** and **6a** in the 4RMG and 5MAT data highlighted several hydrophobic contacts involving the pocket A and B residues (Figure S12). Protein−ligand interactions involving Phe96, Ile118, Phe119, and Ile232 were maintained by both the **2a** and **6a** substituents projected towards pocket B, suggesting both the compounds as putative SIRT2 inhibitors and confirming once again these residues as pivotal to guide the positioning of the inhibitor. In Figure 10, the docking positioning of compound **2a**, taken as one of the most promising thiazoles of the series, is reported.

**Figure 11:**
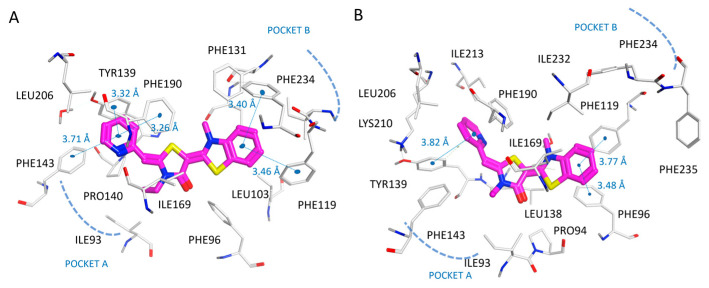
Docking positioning of compound **7a** (YM-08; C atom, magenta) at the 4RMG (**A**) and 5MAT PDB code (**B**) binding site. Most of the potential π–π stacking contacts are shown in light blue and the corresponding distances in Å.

Then, compound **7a** (YM-08) bearing a thiazolinone-based main core as a bioisostere substitution of the thiazole one featured by compounds **1a**–**6a**, together with maintained terminal aromatic features, was also evaluated via molecular docking. As shown in Figure 11, the results pointed out a comparable positioning with respect to the one experienced by the previous **1a**–**6a**, displaying the following: (i) van der Waals contacts and π–π stacking between the bicyclic ring of **7a** and Ile232, Phe96, Phe131, Phe119, and Phe234; (ii) π–π stacking involving the ligand pyridine ring and Tyr139, Phe143, and Phe190; and (iii) hydrophobic contacts from the thiazolinone core and Leu138.

On this basis, the in-house library of compounds **1a**–**6a** and the HSP70 inhibitor **7a** (YM-08) were retained and evaluated via enzymatic assays to ascertain their SIRT2 inhibitory ability.

In addition, the HSP70-targeting activity of **1a**–**6a**, previously reported based on computational methods [27], was also verified by biochemical assays.

### 2.3. Biological Evaluation of Thiazoles ***1a**–**6a*** and of YM-08 (***7a***) as SIRTs Inhibitors

The effect of the in silico screened compounds on the SIRT2 deacetylase activity was evaluated thanks to the incubation of recombinant SIRT2 with an acetylated peptide (H3K9Ac, a peptide acetylated on Lys 9) and NAD^+^. The percentage of SIRT2 activity inhibition obtained with each compound is reported in Table 1 (see dose−response curves in Figure S13).

We subsequently determined the IC_50_ values for **1a** and for the most promising compounds: all of the compounds were found to inhibit SIRT2 activity in the low micromolar range (SIRT2 IC_50_ = 17–45 μM), and were endowed with a comparable SIRT2 inhibitory ability with respect to all of the the series C compounds that were previously discussed (**112**–**116**; SIRT2 IC_50_ = 14.3–77.1 μM), as well as most compounds of series B (**27**–**111**; SIRT2 IC_50_ = 0.39–21.1 μM).

Given that the most used SIRT6 inhibitor also inhibited SIRT2 to a certain extent [43], the inhibition exerted by **1a** against SIRT6 was also determined. Compound **1a** did not significantly affect SIRT6 deacetylase nor the depalmitoylase activity (assayed by adding the palmitoylated peptide H3K9Palm). Thus, compound **1a** was selective over SIRT6.

**Figure 12:**
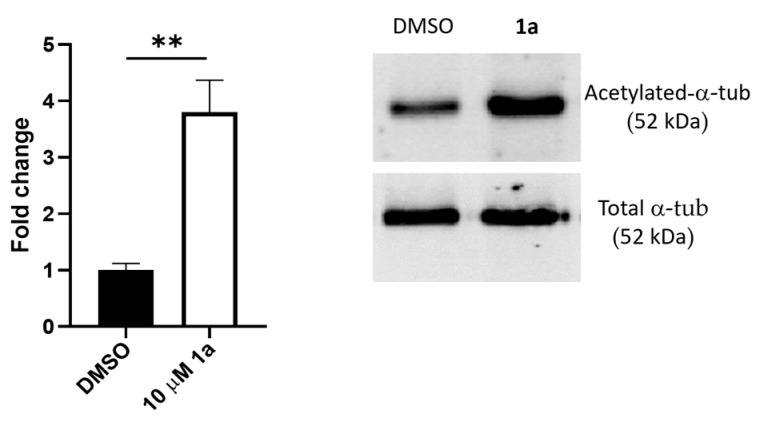
Compound **1a** increased α-tubulin acetylation in the cultured cells. The cells were incubated with 10 μM of compound **1a** or the respective amount of vehicle DMSO, and were used for protein lysate generation; then, the total and acetylated α-tubulin levels were detected by Immunoblotting. The levels of acetylated α-tubulin were quantified, normalized to the total α-tubulin, and expressed as percentage increase vs. vehicle-treated cells. One representative Western blot analysis and the mean ± SD of 3 quantifications are shown. **, *p* < 0.01.

Finally, experiments were performed to determine whether compound **1a** was active in intact cells. The acetylation status of α-tubulin, a known target of SIRT2 deacetylase activity, was used to monitor SIRT2 activity in human cells. As shown in Figure 12 (see also Figure S14), compound **1a** was proven to cross the plasma membrane and inhibit SIRT2 activity, as verified by the higher levels of the acetylated SIRT2 substrate.

### 2.4. Biological Evaluation of Thiazoles ***1a**–**6a*** and of YM-08 (***7a***) as HSP70 Inhibitors

The impact of selected molecules on the ATPase catalytic activity of an in-house produced recombinant human HSP70 protein was determined using the Malachite green assay, incubating the recombinant protein with ATP in the presence or absence of different compounds (300 μM final concentration). The percentage of inhibition exerted by the selected compounds on the ATPase activity was as follow: **1a**, 49 ± 2%; **2a**, 40 ± 1%; **3a**, 44 ± 4%; **4a**, 20 ± 1%; **5a**, 19 ± 1%; **6a**, 37 ± 1%; **7a**, 47 ± 1%.

### 2.5. In Silico Prediction of ADMET Properties

Nowadays, in silico prediction of the absorption, distribution, metabolism, excretion, and toxicity (ADMET) properties is thought to be a promising tool in medicinal chemistry and these techniques are widely exploited in the literature [44,45,46].

Herein, we have developed a perspective on the calculated ADMET properties that explain the drug-like profile of compounds **1a**–**7a** using the ACD/Lab Percepta platform [47] and SwissADME website [48].

Based on Veber’s [49] and Lipinski’s rules [50], the following descriptors have been calculated: (i) logarithmic ratio of the octanol−water partitioning coefficient (cLogP), (ii) molecular weight (MW) of the compounds, (iii) H-bonding acceptor number (HBA), H-bonding donor moieties (HBD), (iv) number of rotatable bonds (nRot_bond), and (v) topological polar surface area (TPSA) (see Table S13).

All of the new derivatives and the reference SIRT2 inhibitors, AGK2 and SirReal2, fulfil most of Lipinski’s rule and Veber’s rule.

Further ADME parameters have been taken into account, such as human intestinal absorption (HIA), estimation of the plasmatic protein binding event (% PPB), volume of distribution (Vd), ligand affinity for human serum albumin (LogKa HSA), and potential oral bioavailability as a percentage (F %) (see Table S13), once again supporting the drug-like profile of these thiazole-based SIRT2 inhibitors. All of them were endowed with comparable or higher F values than AGK2 and SirReal2.

Finally, in silico evaluation of the toxicity properties in terms of cytochrome inhibition and for a lethal dose via oral administration in a mouse model have been explored, as well as PAINS (Pan Assay Interference structures) analysis (Table S14). The results show no cytochrome inhibition events or PAINS ability.

## 3. Discussion

Sirtuin isoform 2 (SIRT2) is one of the seven sirtuin isoforms present in humans. It exhibits a very interesting druggable profile in medicinal chemistry. Currently, a number of X-ray crystal structures for SIRT2 are available and allow for deeper structure-based studies towards the identification of novel SIRT2 modulators.

Herein, we reported molecular docking studies of 116 SIRT2 inhibitors collected from the literature in order to explore different series of chemotype interacting with SIRT2. The first series of calculations involving SirReal2 inhibitors (**1**–**26**) pointed out pivotal interactions at the protein−ligand complex, thanks to aromatic and hydrophobic residues, such as Phe119, Phe234, and Phe235. In addition, the compounds were proven to be stabilized at the biological target via polar contacts with water molecules and the SIRT2 residue Pro94. Comparable contacts were also shown for thienopirimidinone-containing derivatives (**27**–**111**), with additional interactions also being detected, such as π–π stacking involving the terminal naphthyl substituent of the 5MAT co-crystallized ligand and the Tyr139, Phe143, Phe190, and Phe214 residues. Interestingly, polar contacts with Lys210 were proven to be effective.

The subsequent molecular docking studies involving compounds **112**–**116** allowed for considering the role played by smaller and less flexible scaffolds in place of the previous ones within 4RMG and 5MAT. According to our calculations, the small dimension of this series of SIRT2 inhibitors impaired the ligand ability to fit the enzyme cavity properly, as they were not able to interact with Tyr104, Phe119, Phe234, and Phe235 (pocket B). On the other hand, Van der Waals contacts with Pro94, Leu134, and Ile169 and π–π contacts with Phe96 (pocket A) were maintained, revealing these residues as being vital in order to achieve enzyme inhibition. The derived information highlighted key contacts with the SIRT2 binding pocket which allowed us to preliminary evaluate via virtual screening a small in-house library of thiazole-based compounds (**1a**–**7a**) that were previously investigated as putative HSP70 inhibitors.

Notably, as HSP70 inhibition is known to be advantageous for the development of anti-cancer agents [40,41], the identification of dual inhibitors (against HSP70 and SIRT2) may represent a promising strategy for contrasting tumors.

The biological and enzymatic assays confirmed the dual HSP70/SIRT2 inhibitory ability featured by all of the in-house derivatives, with **7a** being the most effective (SIRT2 IC_50_ = 19.9 μM, HSP70 = 47% inhibition). While **2a** was proven to be the most promising new thiazole-based SIRT2 inhibitor investigated herein (SIRT2 IC_50_ = 17.3 μM, HSP70 = 40%), prototype **1a** was the most potent HSP70 inhibitor compared with **7a** (YM-08), and was also endowed with an SIRT2 inhibitory ability.

Notably, the experimental results allowed us to validate the computational studies and to support the development of the thiazole scaffold towards promising novel SIRT2 inhibitors.

## 4. Materials and Methods

### 4.1. Molecular Modeling Studies

All of the studied ligands were manually built in silico using the MOE Builder tool of the MOE2019.01 software [35] and then parametrized (AM1 partial charges as calculation method) and energy minimized with the Energy Minimize tool of the same software, using MMFF94x forcefield. The RMS (root mean square) gradient was equal to 0.0001, and was calculated as the root mean square gradient the norm of the gradient times the square root of the number of (unfixed) atoms. This produced a single low-energy conformation for each ligand [35].

All of the selected X-ray data of SIRT2 in the presence of different inhibitors were downloaded from the Protein Data Bank [17,18] and prepared for the subsequent molecular docking simulation via the QuickPrep module implemented in MOE software.

Re-cross docking simulations were performed using the LeadIT 2.1.8 software suite (www.biosolveit.com) [34] and the MOE Dock Template-based methodology [35]. LeadIT 2.1.8 software relies on the FlexX scoring algorithm, which is able to estimate the binding free energy using the Gibbs−Helmholtz equation [51]. This software detects the binding site using a radius of 10 Å far from the present co-crystallized modulator in order to set up a spherical search space for the following docking calculations. Details of this docking procedure have been previously reported [52,53].

The MOE Dock template-based approach uses the methodology developed for Molecular Superpose to place ligands in the active site based on one or more reference structures (templates), according to flexible alignment. This approach aligns template and input molecules via an undirected heavy atom and projected feature triplet matching scheme. The scoring function incorporates terms for reference and ligand similarity as well as a protein−ligand clash term. The calculation of the enthalpy-based Affinity dG scoring function allowed for ranking 50 poses towards five final ones for each ligand. The enthalpy-based Affinity dG scoring function to score generated poses has been previously reported in the literature [54,55,56].

Regarding compounds **1**–**116** and **1a**–**7a** VS_RP, molecular docking calculations were performed applying the DOCK tool implemented in MOE according to the template similarity methodology, including all of the residues placed at a 4.5 Å distance from the PDEB code co-crystallized inhibitor.

In silico evaluation of the enthalpy-based Affinity dG scoring function allowed for ranking 50 poses towards 10 final ones for each inhibitor (series A–C and **1a**–**7a** via VS_RP).

Then, VS_FP involving **1a**–**7a** was performed by running molecular docking via the Induced Fit method (MOE software) [35]. This procedure allowed for moving the protein binding site as it was flexible during calculations. The Induced Fit method refined each protein−ligand complex to five final docking poses, using the Affinity dG as the definitive scoring function for the final pose ranking.

Details of docking procedures have been previously reported by us [23].

### 4.2. Biological Evaluation of Compounds ***1a**–**7a*** as SIRT2 Inhibitors

The synthesis of peptides H3K9Ac and H3K9Palm and the evaluation of the SIRT2 deacetylase activity and of the SIRT6 deacetylase and depalmitoylase activity were carried out as previously described [23]. The IC_50_ of compound **1a** was determined as in [43].

The bronchial epithelial CFBE41o- cells were cultured as in [20]. The cells (5 × 10^5^) were plated in 100 mm cell culture dishes and allowed to adhere for 24 h. Thereafter, the cells were incubated with 10 μM of compound **1a** or the respective amount of vehicle DMSO (0.1% final concentration in the cell culture). The cells were lysed in a lysis buffer (25 mM Tris-HCl, pH 7.8, 2 mM DTT, 2 mM EDTA, 10% glycerol, and 1% Triton X-100, and protease inhibitor cocktail). Thirty micrograms of the proteins were loaded on a 10% polyacrylamide gel and separated using SDS-PAGE. The proteins were subsequently transferred to nitrocellulose membranes and the expression of the total or acetylated α-tubulin was detected using the following primary antibodies: anti-α-tubulin (rabbit polyclonal, #2125, Cell Signalling Technology, Danvers, MA, USA) and anti-acetylated α-tubulin (rabbit polyclonal, #5335, Cell Signalling Technology). Following incubation with the appropriate secondary antibodies and Amersham ECL detection (Cytiva, Milano, Italy), the band intensity was quantified using the ChemiDoc imaging system (Bio-Rad, Milan, Italy). α-tubulin acetylation was normalized to the total α-tubulin levels.

### 4.3. Biological Evaluation of Compounds ***1a**–**7a*** as HSP70 Inhibitors

The recombinant human Hsp70 protein was produced in BL21 (DE3) *E. coli* transformed with the pET-6xHis/hHSP70 plasmid containing the coding sequence for human HSP70 (hHSPA1A gene, NM_005345.6; purchased from Vector Builder, Vector ID VB210930). This vector allowed for producing an N-terminal 6xHis tag fusion HSP70 protein.

BL21 cells containing the vector were initially grown in Luria−Bertani medium at 37 °C (Merck, Milan, Italy) with 200 μg/mL ampicillin until the culture reached an OD_600_ of 0.5. The 6xHis/hHSP70 fusion protein was expressed by adding isopropyl-β-d-thiogalactopyranoside (final concentration of 0.2 mM) and incubating the bacteria culture at 37 °C for 2 h. The cells were harvested by centrifugation and then lysed by sonication in 20 mM Tris-HCl, 250 mM KCl, and 10 mM MgCl_2_ pH 7.4; after the addition of Triton X-100 to a final concentration of 1% and incubation for 30 min at 4 °C, the lysates were centrifugated at 10,000× *g* for 15 min. The HSP70 protein was purified by affinity chromatography using His GraviTrap TALON columns (Euroclone, Milan, Italy). Briefly, after loading the lysate, the resin was washed twice with 20 mM Tris-HCl, 250 mM KCl, and 10 mM MgCl_2_ pH 7.4 and twice with the same buffer containing 10 mM of imidazole. The elution of the recombinant protein was obtained with 150 mM imidazole, pH 7.4. Imidazole was removed from the recombinant protein through overnight dialysis using a dialysis tubing membrane (Merck KGaA, Frankfurter Strasse 250 Darmstadt, Germany) and the protein was concentrated using a Centricon (Merck Millipore, 11 Avenida Norte Bis No. 513, San Salvador, El Salvador). The protein concentrations were determined using the Bradford assay (Bio-Rad Laboratories, Inc. Italy, Segrate) and the protein purity was monitored using SDS-PAGE.

The ATPase catalytic activity of recombinant human HSP70 was measured with a colorimetric assay using the malachite green reagent. Briefly, 6 µM of HSP70 protein was incubated for 30 min at 37 °C with 5 mM ATP in 20 mM Tris-HCl, 250 mM KCl, 10 mM MgCl_2_, and 0.02%TritonX-100, pH 7.4. The released phosphates were quantified by adding the malachite green reagent to an aliquot of this incubation and the absorbance was recorded at 620 nm using a Clariostar plate reader. The effects of thiazoles-based molecules on the ATPase catalytic activity were measured by adding (or not) the different molecules to the incubation (at 300 µM final concentration). To correct for non-enzymatic hydrolysis of ATP and for a specific absorbance of molecules, the absorbance signal of identically treated samples lacking the HSP70 protein was subtracted.

### 4.4. In Silico Prediction of ADMET Properties

The predictive evaluation of all of the cited ADMET parameters was performed by means of the Advanced Chemistry Development (ACD) using the Percepta platform [47]. The software prediction relies on the software-implemented training libraries, which include experimentally determined pharmacokinetic and safety properties regarding different series of ligands. The prediction of PAINS (Pan Assay Interference structures) was managed via the SwissADME website [48], as a useful approached described in the literature [57,58,59].

## 5. Conclusions

Recently, a number of X-ray crystal structures of SIRT2 have become available, paving the way for setting up structure-based studies for new putative SIRT2-targeting compounds.

Herein, we collected and explored the binding mode of SIRT2 inhibitors exhibiting different scaffolds, relying on comparisons between several SIRT2 X-ray data. The computational studies were performed in tandem with extensive molecular docking calculations, and led to new insights regarding the SIRT2 inhibitor binding mode. The obtained results pointed out key hydrophobic contacts as the driving force to stabilize the enzyme−ligand complex. In particular, binding to Phe143, Phe190, Leu206, and Ile213 was proven to be effective at stabilizing the enzyme modulator, while further Van der Waals and π–π interactions with Phe131, Phe234, or Phe96, Phe119 (pocket A and B) are expected to contribute to the inhibitor efficiency. This suggests there should be subsequent VS of in-house compounds, including thiazole/thiazolinone-based derivatives (**1a**–**7a**), previously explored in silico as HSP70 inhibitors. Biological and enzymatic assays validated compounds **1a**–**7a** as SIRT2 and HSP70 inhibitors, suggesting compounds **2a** and **7a** (with SIRT2 IC_50_ = 17.3 and 19.9 μM, respectively) as being the most promising derivatives proposed herein. Accompanying in silico ADMET prediction studies supported both molecules as candidates for the development of putative drug-like compounds.

## Figures and Tables

**Table 1:** Compound activity as putative inhibitors of SIRT2, tested on the recombinant protein (n.d., not determined).

Compound	% Inhibition SIRT2 (at 150 μM)	SIRT2 IC_50_ (μM)
**1a**	72 ± 8	45.1 ± 5.0
**2a**	100 ± 9	17.3 ± 2.0
**3a**	60 ± 8	n.d.
**4a**	60 ± 5	n.d.
**5a**	76 ± 9	n.d.
**6a**	92 ± 7	27.3 ± 4.7
**7a**	93 ± 9	19.9 ± 2.1
**SirReal2**	100 ± 5	n.d.
**AGK2**	97 ± 5	n.d.

## Data Availability

Data is contained within the article and supplementary material.

## Supplementary Materials

The following supporting information can be downloaded at: https://www.mdpi.com/article/10.3390/ph16091316/s1: Figure S1. Scheme of the most relevant interactions involving the SIRT2 inhibitor SirReal2 (3TE) and the biological target (PDB code = 4RMG). Figure S2: Scheme of the most relevant interactions involving the selective thienopyrimidinone based SIRT2 inhibitor (7KJ) and the biological target (PDB code = 5MAT). Figure S3: Comparison of the best scored 4RMG and 5MAT ligand docking poses with respect to the same crystallized compounds at the corresponding 4RMG and 5MAT PDB codes. Figure S4: RMSD values (Å) as obtained by superimposition of 4RMG and 5MAT. Figure S5: Superimposition of the 4RMG and 5MAT PDB codes in presence of the co-crystallized ligands. Figure S6: Docking positioning of SirReal2 and of the analogue **11** at the 4RMG binding site. Figure S7: Docking positioning of compound **25** and of **23** at the 4RMG binding site. Figure S8: Docking positioning of compound **36** and of **94** at the 5MAT binding site. Figure S9: Docking positioning of compound **49** and of **56** at the 5MAT binding site. Figure S10: Docking positioning of compound **62** and of **66** (C atom; magenta) at the 5MAT binding site. Figure S11: Docking positioning of compound **86** and of **90** at the 5MAT binding site. Figure S12: Docking positioning of compound **2a** and of **6a** at the 4RMG and at the 5MAT PDB code binding site. Figure S13: Concentration-response plots of the putative SIRT2 inhibitors, tested on SIRT2 recombinant protein. Figure S14: Compound **1a** increase α-tubulin acetylation in cultured cells. The three performed Western blot analysis are shown. Table S1: Chemical structure and SIRT2 inhibitory ability featured by compounds **1**–**26**; Table S2: Chemical structure and SIRT2 inhibitory ability featured by compounds **27**–**111**; Table S3: Chemical structure and SIRT2 inhibitory ability featured by compounds **112**–**116**; Table S4: Five top scored re-docking positioning of the 4RMG and 5MAT co-crystallized ligands; Table S5: Ten top scored docking positioning of SirReal2 at the 4RMG PDB code; Table S6: Ten top scored docking positioning of the 5MAT co-crystallized ligand and of the related analogues at the 5MAT PDB code; Table S7: Ten top scored docking positioning of the SIRT2 inhibitors **112**–**116** at the 4RMG PDB code; Table S8: Ten top scored docking positioning of the SIRT2 inhibitors **112**–**116** at the 5MAT PDB code; Table S9: Rigid protein Virtual screening. Ten top scored docking positioning of **1a**–**7a** at the 4RMG PDB code; Table S10: Rigid protein Virtual screening. Ten top scored docking positioning of **1a**–**7a** at the 5MAT PDB code; Table S11: Flexible protein Virtual screening. Five top scored docking positioning of **1a**–**7a** at the 4RMG PDB code; Table S12: Flexible protein Virtual screening. Five top scored docking positioning of **1a**–**7a** via at the 5MAT PDB code; Table S13: Calculated propertied based on the Lipinski’s and Veber’s rules as referred to **1a**–**7a** and to the reference compounds AGK2, SirReal2; Table S14: Calculated ADMET descriptors concerning **1a**–**7a** and the reference compounds AGK2, SirReal2.
