# Supplementary material for: Novel Thiazole-Based SIRT2 Inhibitors Discovered via Molecular Modelling Studies and Enzymatic Assays
Source: Pharmaceuticals (Basel). 2023 Sep 18;16(9):1316. doi: 10.3390/ph16091316 (PMC10535842; doi:10.3390/ph16091316)
Supplement: Supplementary file 1 [file pharmaceuticals-16-01316-s001.zip › pharmaceuticals-2556008-supplementary.pdf]

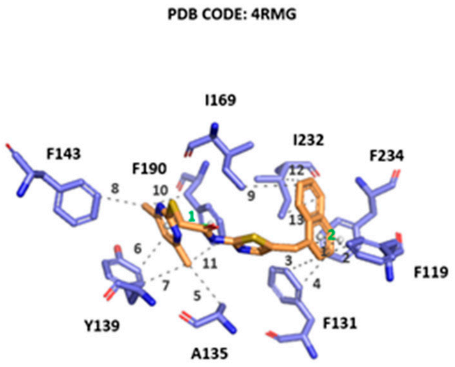

Hydrophobic interactions

Index	Residue number	Residue	Distance	Ligand Atom
1	119A	PHE	3.51	2357
2	119A	PHE	3.45	2378
3	131A	PHE	3.82	2378
4	131A	PHE	3.64	2379
5	135A	ALA	3.91	2373
6	139A	TYR	3.60	2374
7	139A	TYR	3.79	2373
8	143A	PHE	3.74	2376
9	169A	ILE	3.77	2362
10	190A	PHE	3.54	2376
11	190A	PHE	3.45	2373
12	232A	ILE	3.85	2364
13	232A	ILE	3.45	2365

π-Stacking

Index	Residue number	Residue	Distance	Ligand atom
1	190A	PHE	4.05	2363, 2370, 2371, 2372, 2374, 2375
2	234A	PHE	4.86	2357, 2358, 2378, 2379, 2380, 2381

Figure S1. Scheme of the most relevant interactions involving the SIRT2 inhibitor SirReal2 (3TE) and the biological target (*PDB code = 4RMG*) [22]. Distance values are reported in Å. Hydrophobic interactions are represented as grey dotted lines, and indexed with dark grey numbers. π-Stacking are represented as dark green long-dash dotted lines, and indexed with green numbers. Hydrogen bonds are represented as blue solid lines, indexes with red numbers. Water bridges are indicated with light blue solid lines, indexes with blue numbers. The white spheres indicate the center of an aromatic ring (involved in pi-stacking).

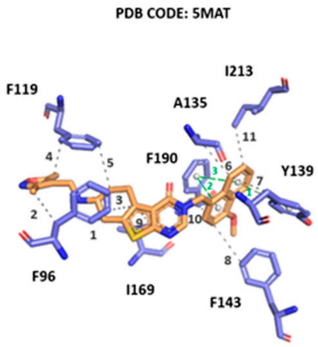

Hydrophobic interactions

Index	Residue number	Residue	Distance	Ligand Atom
1	96A	PHE	3.36	9626
2	96A	PHE	3.80	9635
3	96A	PHE	3.75	9624
4	119A	PHE	3.77	9631
5	119A	PHE	3.85	9628
6	135A	ALA	3.95	9611
7	139A	TYR	3.98	9611
8	143A	PHE	3.91	9616
9	169A	ILE	3.58	9624
10	190A	PHE	3.99	9616
11	213A	ILE	3.88	9610

π-Stacking

Index	Residue number	Residue	Distance	Ligand atom
1	139A	TYR	4.66	9608, 9609, 9610, 9611, 9612, 9613
2	190A	PHE	3.80	9612, 9613, 9614, 9615, 9616, 9617
3	190A	PHE	4.04	9608, 9609, 9610, 9611, 9612, 9613

Figure S2. Scheme of the most relevant interactions involving the selective thienopyrimidinone based SIRT2 inhibitor (7KJ) and the biological target (*PDB code = 5MAT*) [25]. Distance values are reported in Å. Hydrophobic interactions are represented as grey dotted lines, and indexed with dark grey numbers. π-Stacking are represented as dark green long-dash dotted lines, and indexed with green numbers. Hydrogen bonds are represented as blue solid lines, indexes with red numbers. Water bridges are indicated with light blue solid lines, indexes with blue numbers. The white spheres indicate the center of an aromatic ring (involved in pi-stacking).

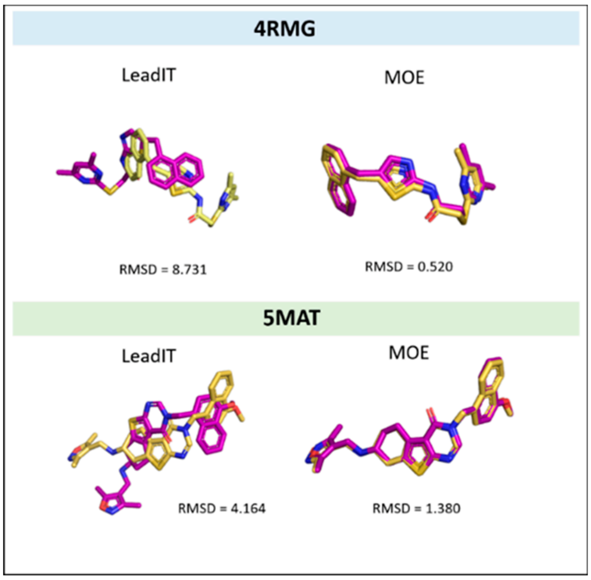

Figure S3. Comparison of the best scored 4RMG and 5MAT ligand docking poses (purple ligand) with respect to the same crystallized compounds at the corresponding 4RMG and 5MAT PDB codes (yellow ligand) as obtained by LeadIT and MOE molecular docking. RMSD values (Å) have been evaluated by Pymol [The PyMOL Molecular Graphics System, Version 1.2r3pre, Schrödinger, LLC].

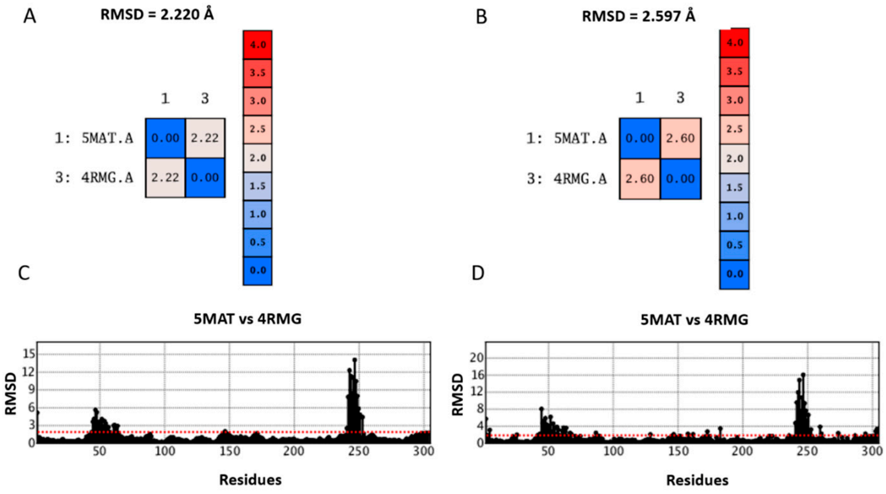

Figure S4. RMSD values (Å) as obtained by superimposition of 4RMG and 5MAT are shown based on the carbon atom alignment (A) and at the whole structure (B). The corresponding overall RMSD variation trend is also reported (C,D).

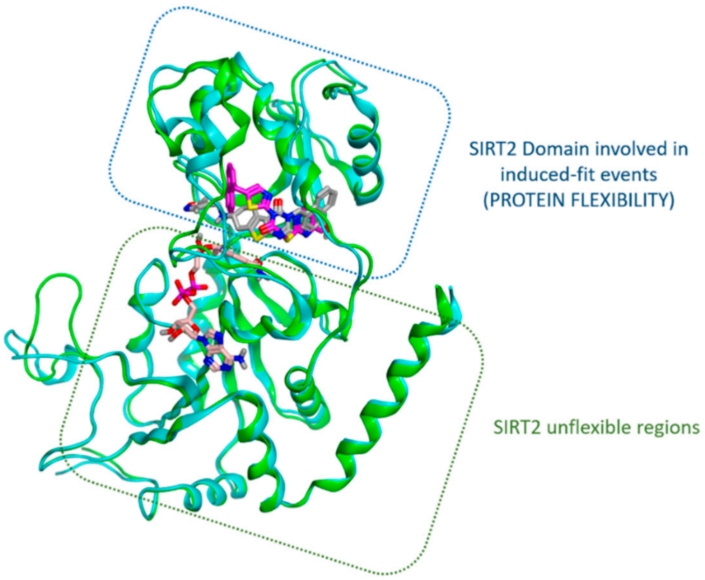

Figure S5. Superimposition of the 4RMG (in cyan) and 5MAT (in green) PDB codes in presence of the co-crystallized ligands is also depicted. Most flexible areas due to the inhibitor structure are also highlighted.

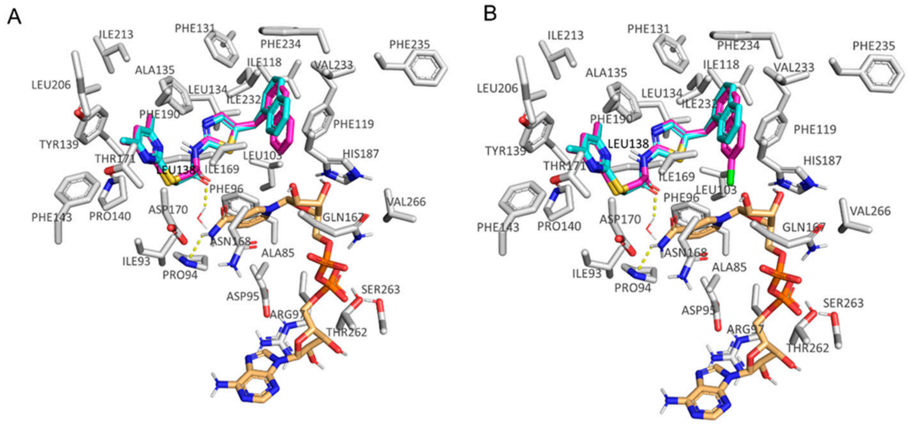

Figure S6 Docking positioning of SirReal2 (C atom, magenta) (A) and of the analogue **11** (C atom; magenta) (B) at the 4RMG binding site. The co-crystallized SirReal2 (C atom; cyan) and NAD^+ (C atom; light orange) structures are also reported.

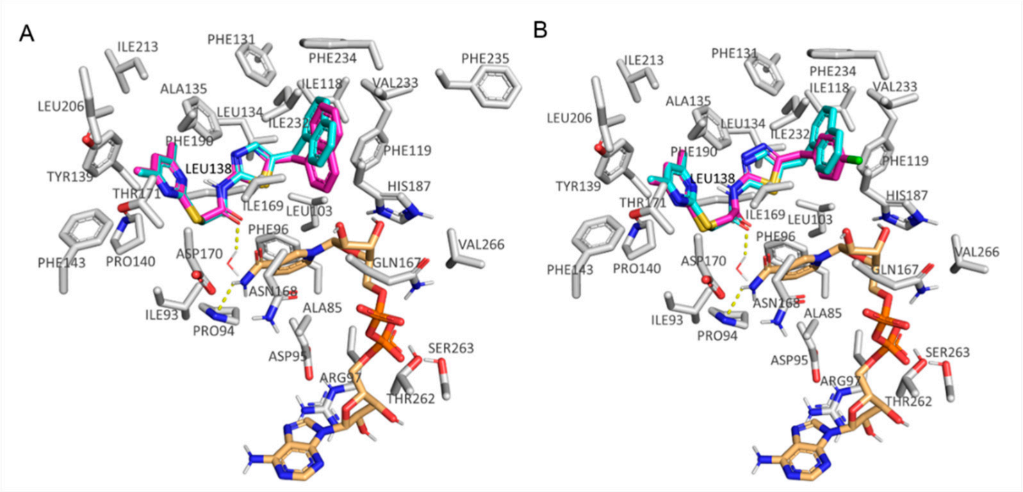

Figure S7 Docking positioning of compound **25** (C atom, magenta) (A) and of **23** (C atom; magenta) (B) at the 4RMG binding site. The co-crystallized SirReal2 (C atom; cyan) and NAD+ (C atom; light orange) structures are also reported.

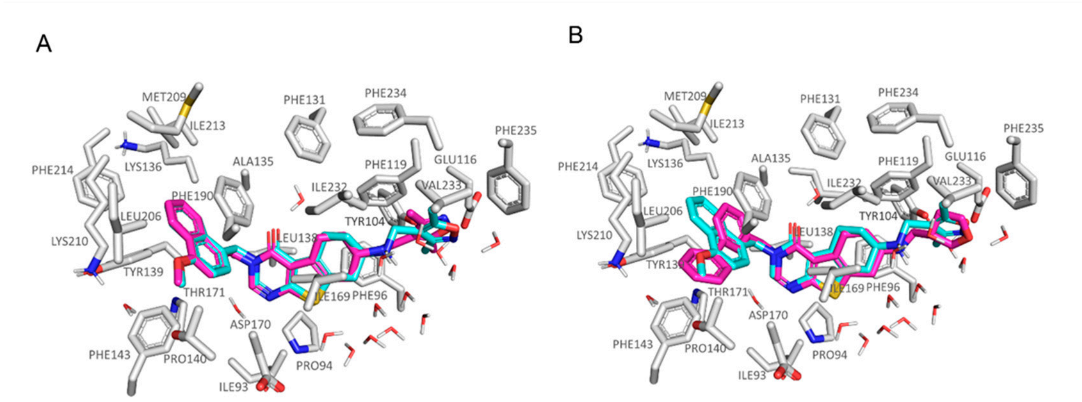

Figure S8. Docking positioning of compound **36** (C atom, magenta) (A) and of **94** (C atom; magenta) (B) at the 5MAT binding site. The co-crystallized thienopyrimidinone inhibitor **36** (C atom; cyan) structure is also reported.

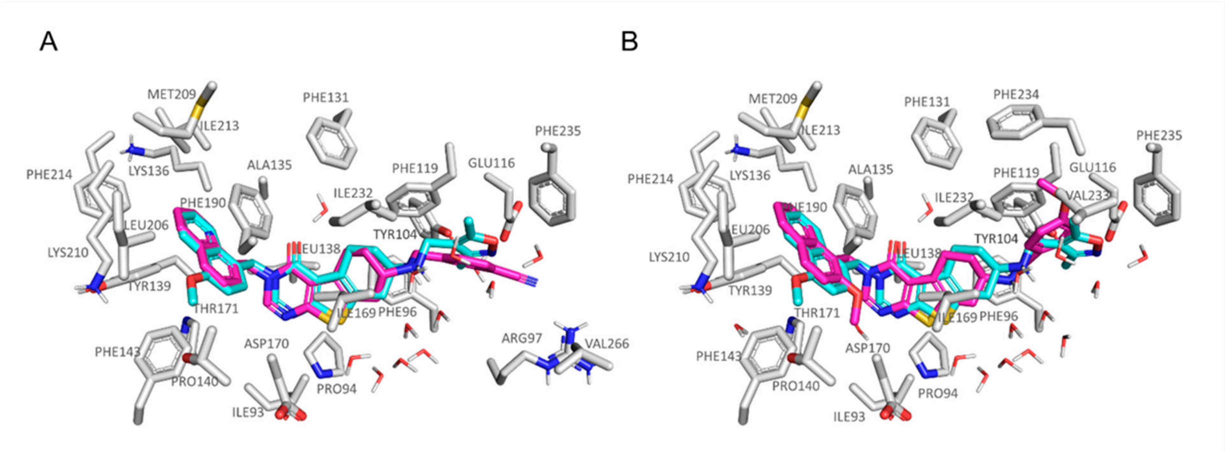

Figure S9. Docking positioning of compound **49** (C atom, magenta) (A) and of **56** (C atom; magenta) (B) at the 5MAT binding site. The co-crystallized thienopyrimidinone inhibitor **36** (C atom; cyan) structure is also reported.

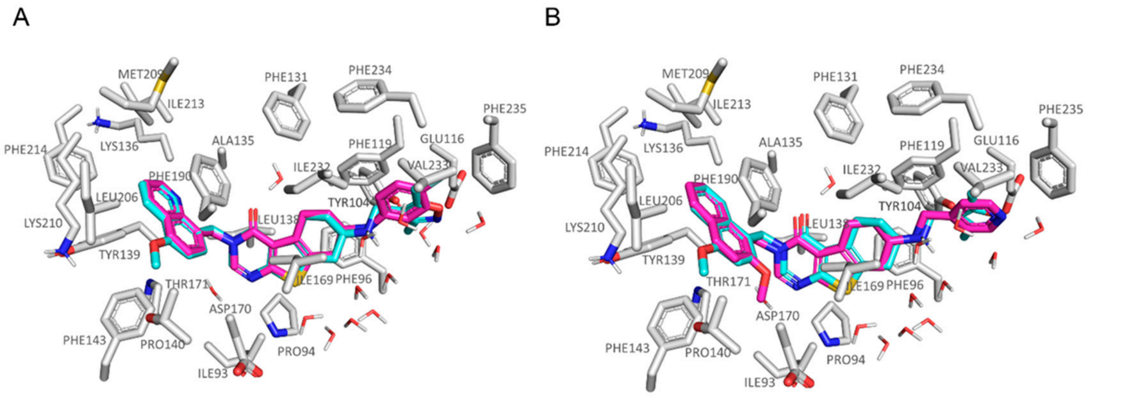

Figure S10. Docking positioning of compound **62** (C atom, magenta) (A) and of **66** (C atom; magenta) (B) at the 5MAT binding site. The co-crystallized thienopyrimidinone inhibitor **36** (C atom; cyan) structure is also reported.

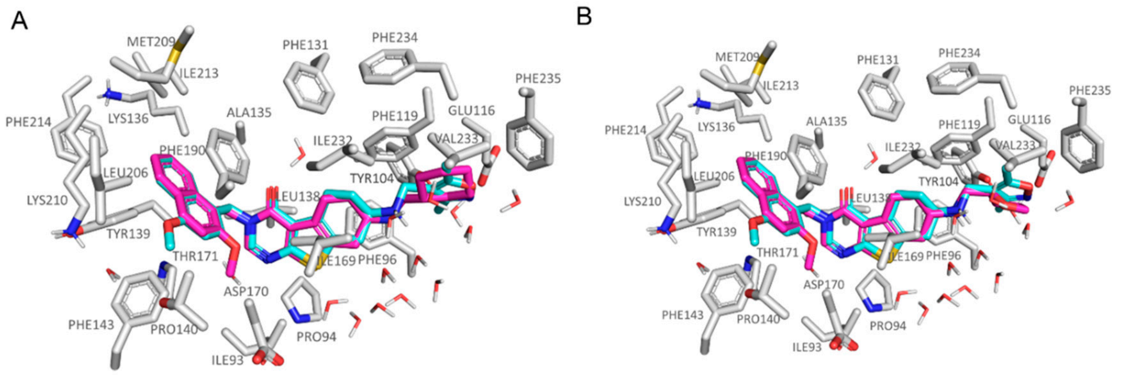

Figure S11. Docking positioning of compound **86** (C atom, magenta) (A) and of **90** (C atom; magenta) (B) at the 5MAT binding site. The co-crystallized thienopyrimidinone inhibitor **36** (C atom; cyan) structure is also reported.

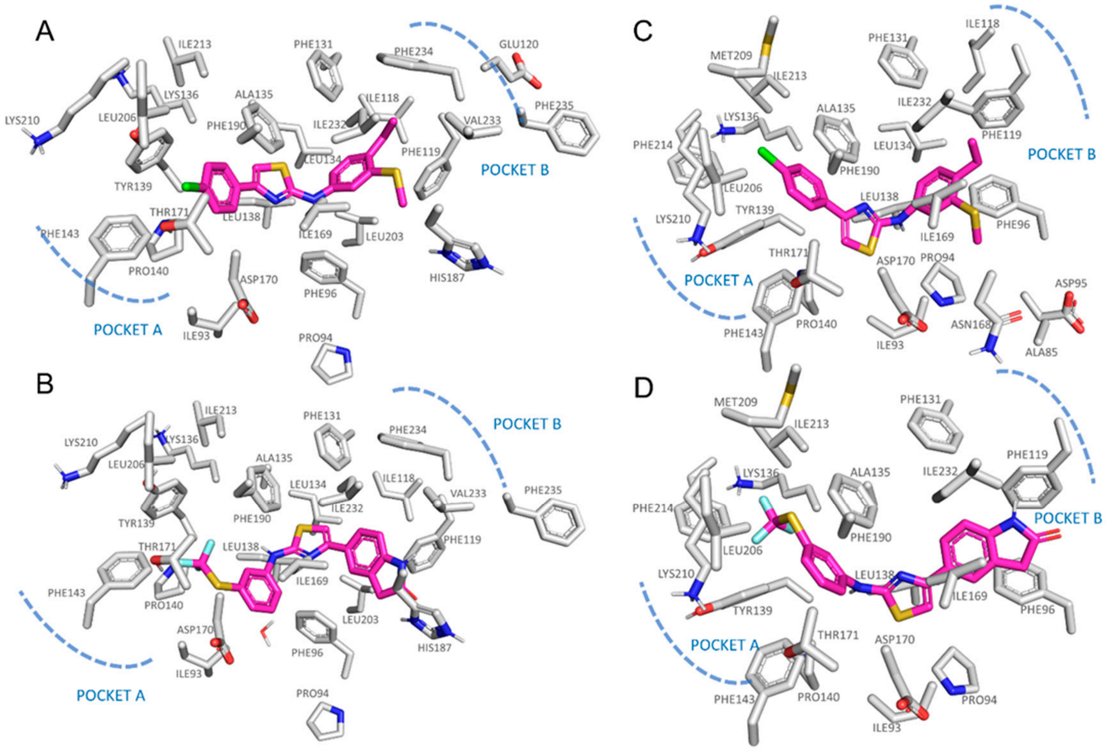

Figure S12. Docking positioning of compound **2a** (C atom, magenta) at the 4RMG (A) and at the 5MAT PDB code (C) binding site. The docking mode of the analogue **6a** (taken as representative of the most related analogues of YM-08 herein described) at the 4RMG (B) and at the 5MAT PDB code (D) binding site is also reported.

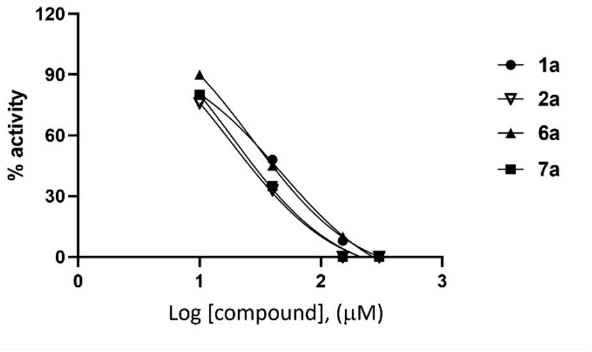

Figure S13. Concentration-response plots of the putative SIRT2 inhibitors, tested on SIRT2 recombinant protein. Data are plotted as % activity vs. inhibitor logarithmic micromolar concentration.

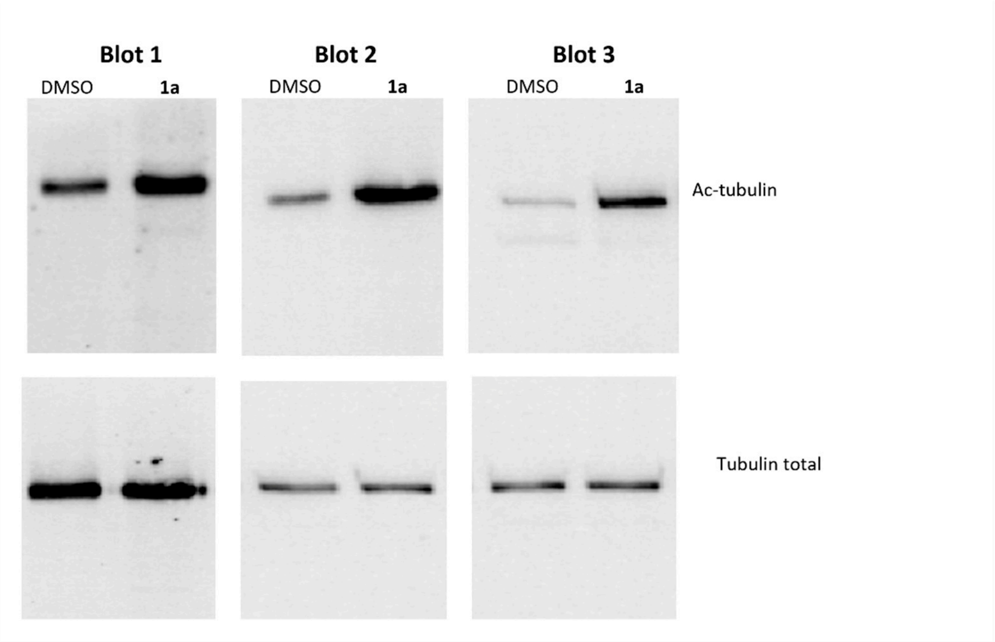

Figure S14. Compound **1a** increase α-tubulin acetylation in cultured cells. Cells were incubated with 10 μM compound **1a**, or the respective amount of vehicle DMSO, and used for protein lysate generation, and total and acetylated α-tubulin levels were detected by Immunoblotting. The three performed Western blot analysis are shown.

Table S1. Chemical structure and SIRT2 inhibitory ability featured by compounds **1-26** (as SirReal2 analogues) [24].

Compound	Chemical Structure	IC_{50} (μM)
**1**	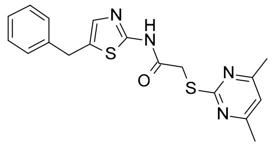	3.75
**2**	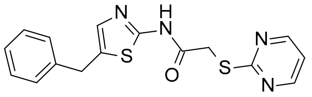	16.8
**3**	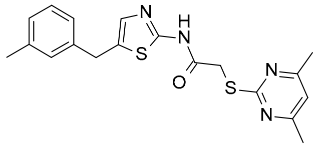	1.64
**4**	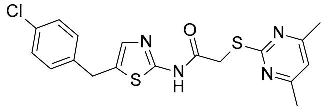	3.40
**5**	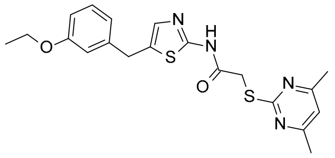	1.33
**6**	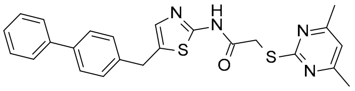	164.5
**7**	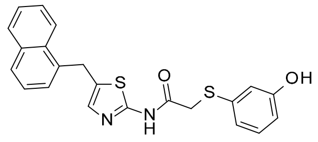	143
**8**	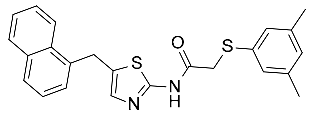	207
**9**	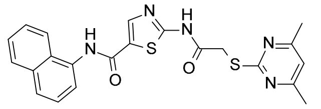	33
**10** (SirReal2)	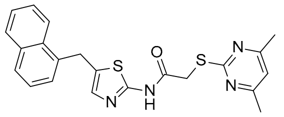	0.44
**11**	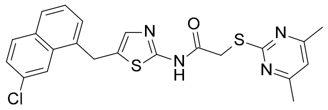	0.18
**12**	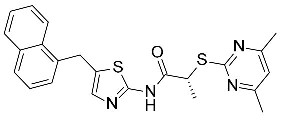	9.77
**13**	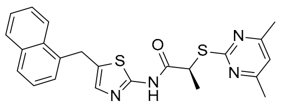	0.26

**14**	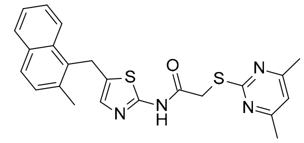	0.31
**15**	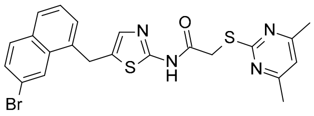	0.21
**16**	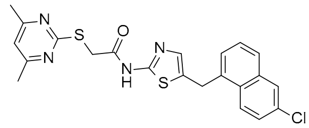	0.48
**17**	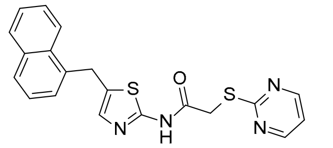	2.34
**18**	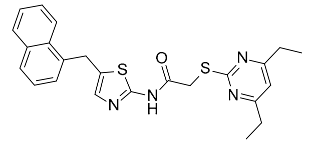	45.6
**19**	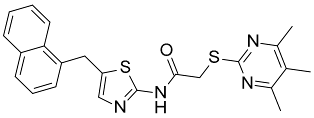	15.0
**20**	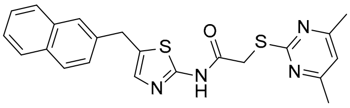	65
**21**	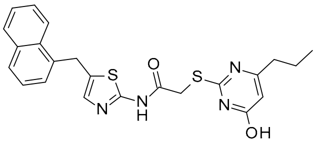	127.2
**22**	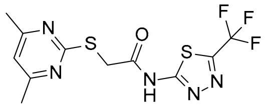	502.8
**23**	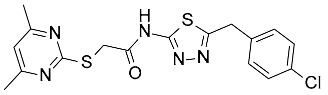	30.9
**24**	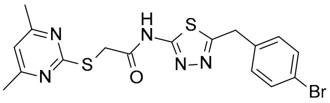	167.7
**25**	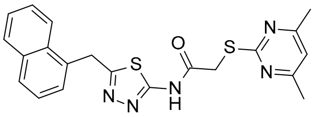	1.89
**26**	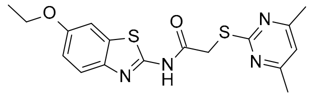	207

Table S2. Chemical structure and SIRT2 inhibitory ability featured by compounds **27-111** [25].

Compound	Chemical Structure	IC_{50} (μM)

**27**	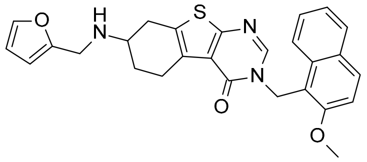	5.24
**28**	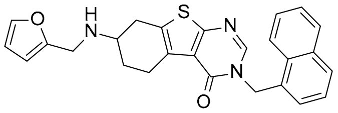	7.61
**29**	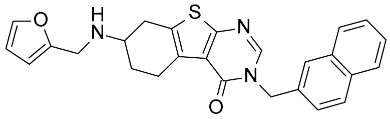	11.68
**30**	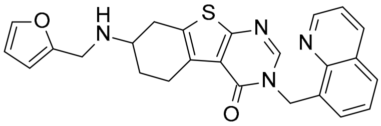	4.16
**31**	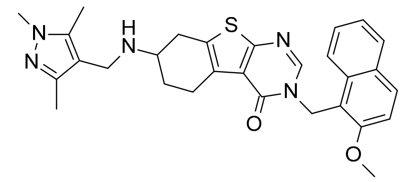	5.11
**32**	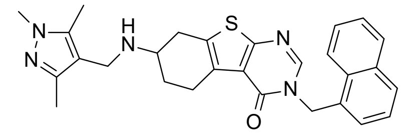	4.20
**33**	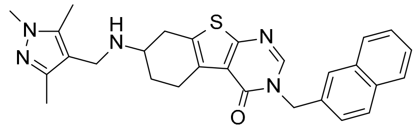	7.43
**34**	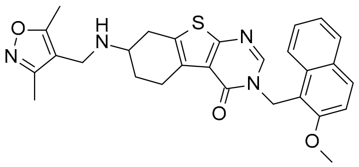	4.00
**35**	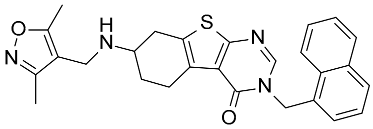	1.85
**36**	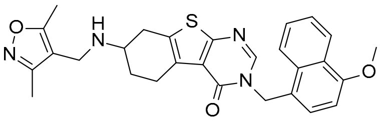	0.58
**37**	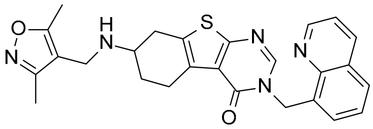	6.94
**38**	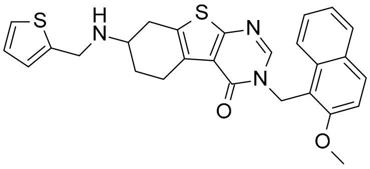	6.03

**39**	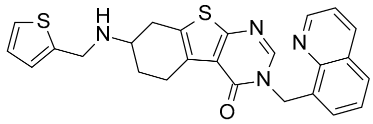	2.56
**40**	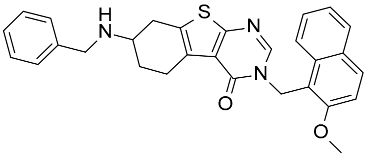	21.10
**41**	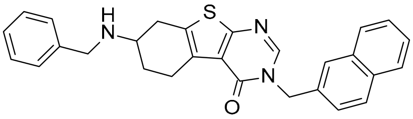	5.45
**42**	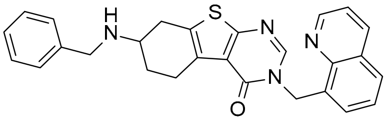	2.50
**43**	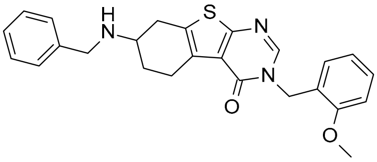	10.42
**44**	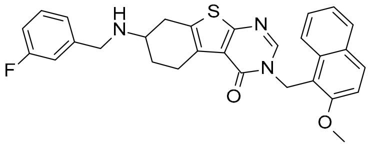	4.07
**45**	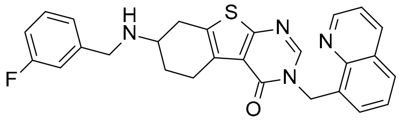	1.72
**46**	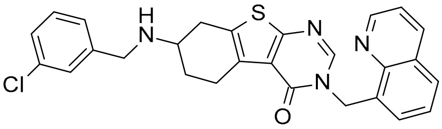	2.84
**47**	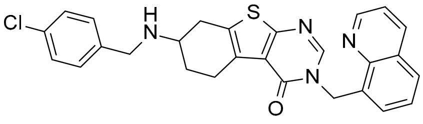	1.27
**48**	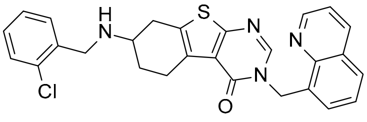	3.80
**49**	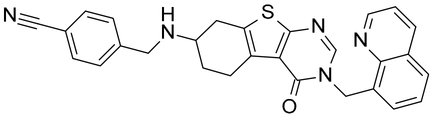	0.39
**50**	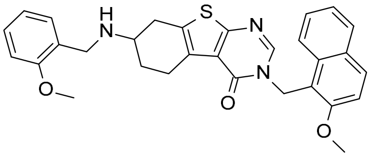	2.24

**51**	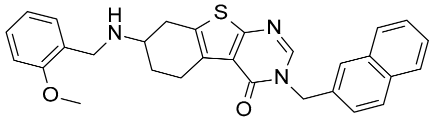	4.22
**52**	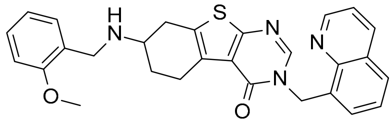	5.61
**53**	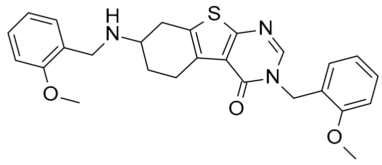	9.59
**54**	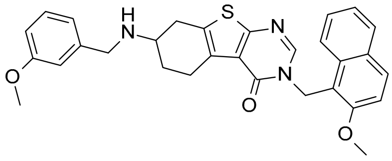	1.90
**55**	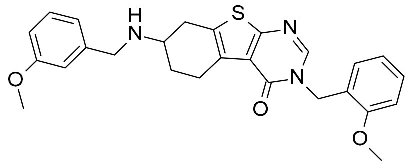	13.40
**56**	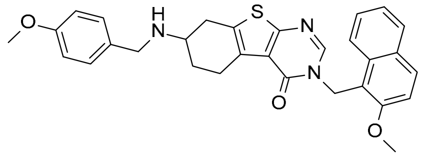	0.65
**57**	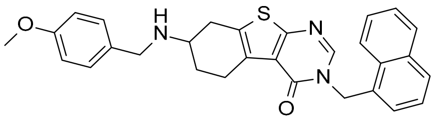	3.2
**58**	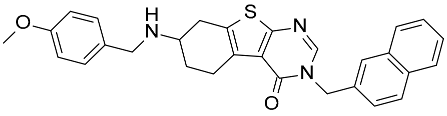	3.82
**59**	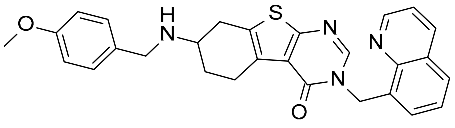	1.83
**60**	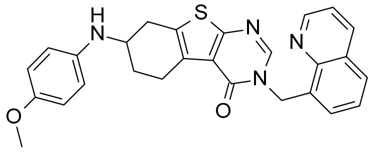	2.48
**61**	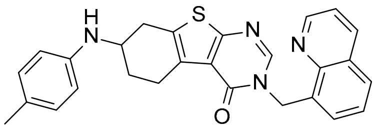	4.25
**62**	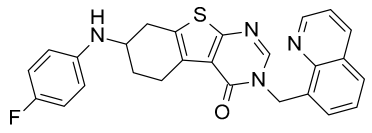	0.97

**63**	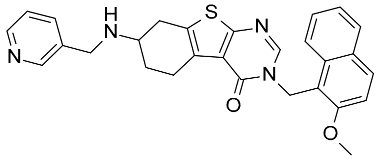	1.45
**64**	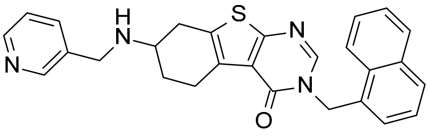	2.99
**65**	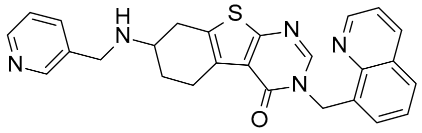	2.92
**66**	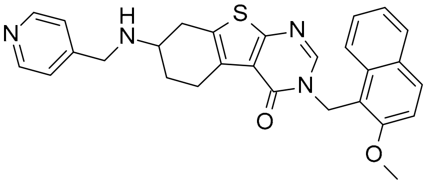	1.20
**67**	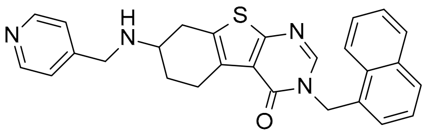	3.58
**68**	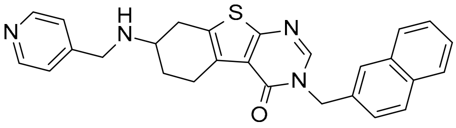	3.14
**69**	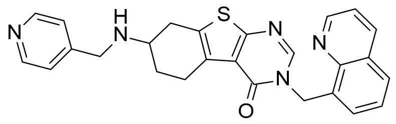	3.68
**70**	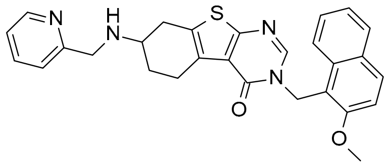	3.12
**71**	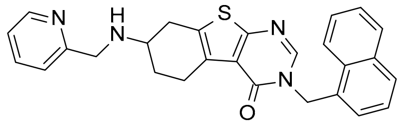	7.68
**72**	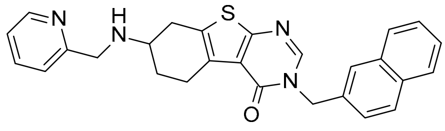	8.67
**73**	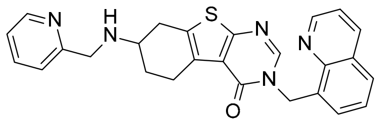	2.61
**74**	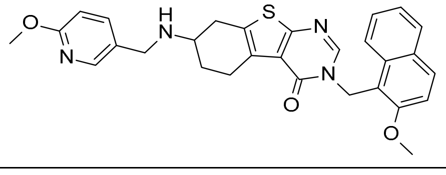	1.9

**75**	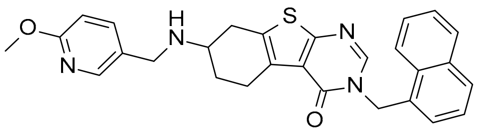	7.59
**76**	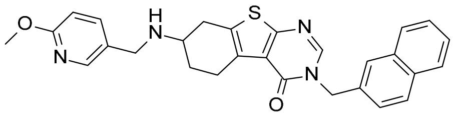	10.17
**77**	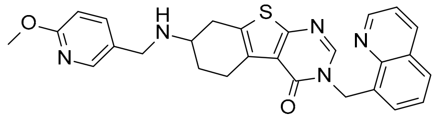	2.65
**78**	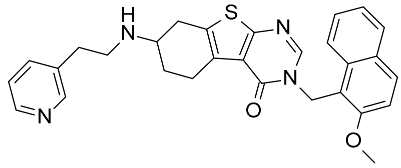	10.31
**79**	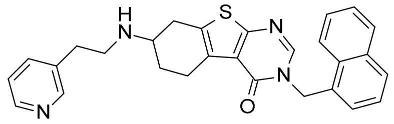	9.57
**80**	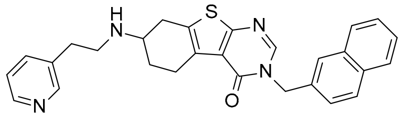	8.68
**81**	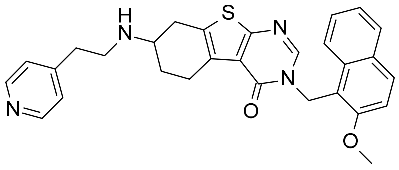	2.75
**82**	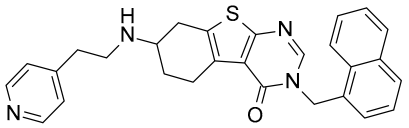	5.79
**83**	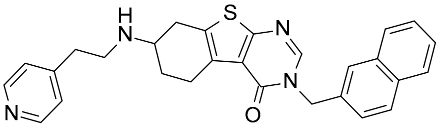	9.60
**84**	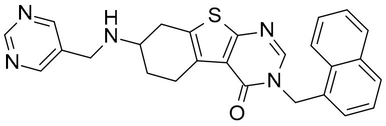	6.45
**85**	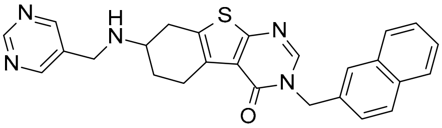	5.30
**86**	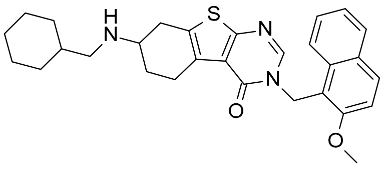	1.65

**87**	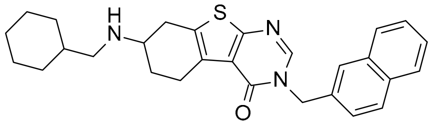	5.53
**88**	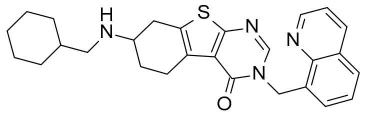	2.43
**89**	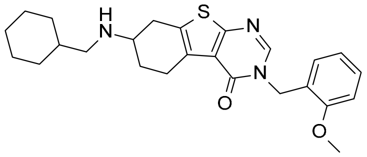	9.18
**90**	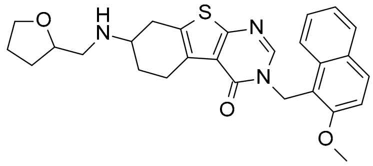	1.90
**91**	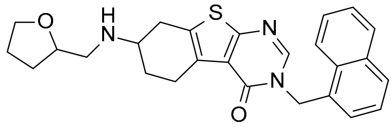	12.48
**92**	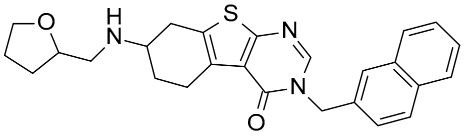	13.14
**93**	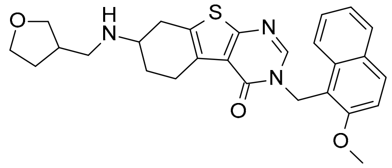	6.03
**94**	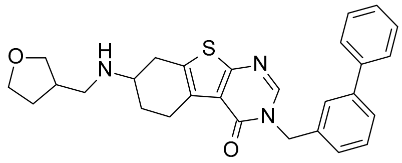	2.58
**95**	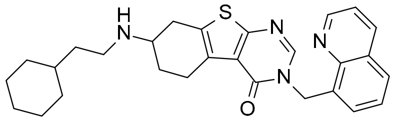	5.85
**96**	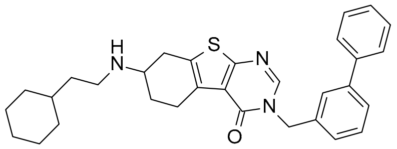	3.74
**97**	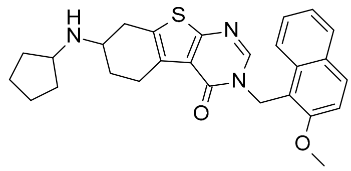	1.18

**98**	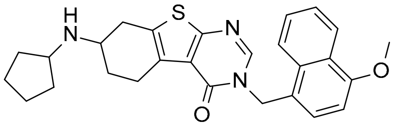	0.73
**99**	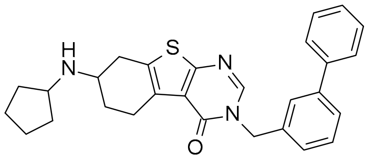	1.52
**100**	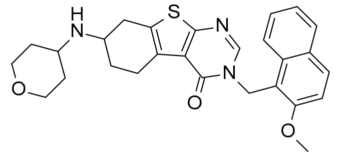	1.11
**101**	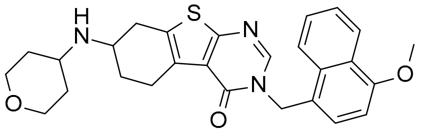	5.06
**102**	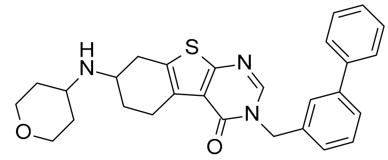	1.74
**103**	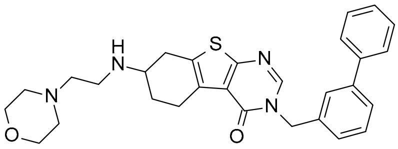	3.14
**104**	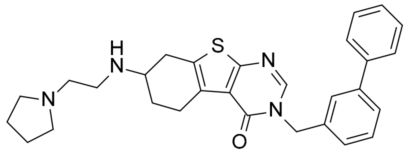	3.78
**105**	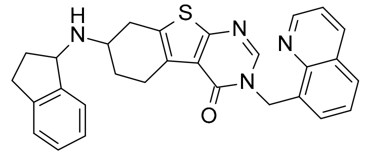	3.09
**106**	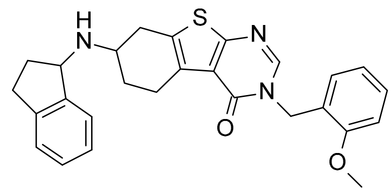	16.32
**107**	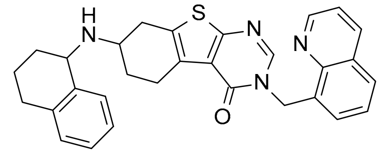	5.49
**108**	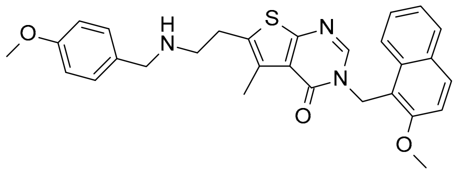	3.36

**109**	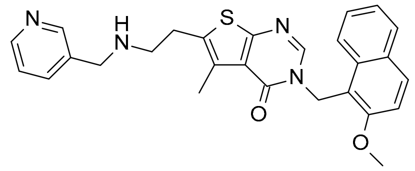	2.15
**110**	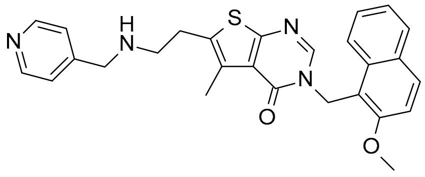	2.93
**111**	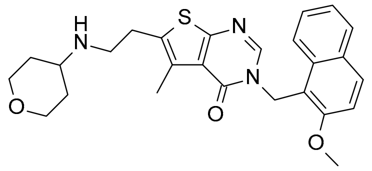	8.15

Table S3. Chemical structure and SIRT2 inhibitory ability featured by compounds **112-116** ([26].

Compound	Chemical Structure	IC_{50} (μM)
**112**	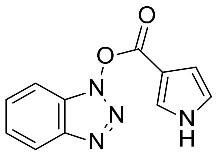	56.6
**113**	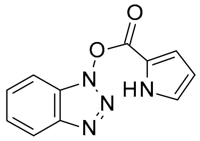	42.7
**114**	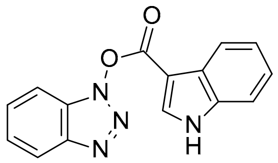	14.3
**115**	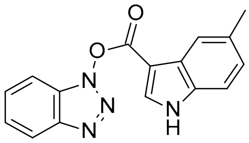	74.0
**116**	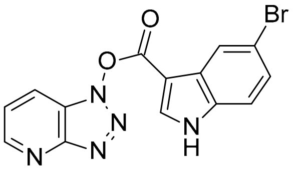	77.1

Table S4. Five top scored re-docking positioning of the 4RMG and 5MAT co-crystallized ligands at the corresponding experimental data, based on the LeadIT and MOE software (see material and method section for details). The predicted ΔG value of each protein-ligand complex has been reported.

	LeadIT re-docking	MOE re-docking

SIRT2 co-crystallized inhibitor	4RMG Binding Affinity Energy (kJ/mol)	5MAT Binding Affinity Energy (kJ/mol)	4RMG Binding Affinity Energy (kcal/mol)	5MAT Binding Affinity Energy (kcal/mol)
4RMG ligand pose 1	-25.9191	-	-10,3013	-
4RMG ligand pose 2	-25.7311	-	-9,94133	-
4RMG ligand pose 3	-24.9027	-	-9,62434	-
4RMG ligand pose 4	-24.8213	-	-9,55069	-
4RMG ligand pose 5	-24.2870	-	-9,52854	-
5MAT ligand pose 1	-	-26.1502	-	-13,602593
5MAT ligand pose 2	-	-24.6139	-	-13,588899
5MAT ligand pose 3	-	-22.7001	-	-13,042794
5MAT ligand pose 4	-	-21.7195	-	-12,688121
5MAT ligand pose 5	-	-21.6132	-	-12,586191

Table S5. Ten top scored docking positioning of SirReal2 (**10**) and of the related analogues (**1**-**26**) at the 4RMG PDB code (MOE software). The predicted ΔG value of each protein-ligand complex has been reported, as calculated in terms of final scoring function (S, as Kcal/mol).

Compound	S	E_conf	E_place	E_score1	E_refine	E_score2
**1**	-9.6736	-33.1359	-22.7067	-8.4093	-8.3120	-9.6736
**1**	-9.0728	-47.8155	-23.2848	-9.8777	-26.1328	-9.0728
**1**	-8.9807	-44.9250	-33.6730	-10.8258	-23.7357	-8.9807
**1**	-8.9344	-41.2470	-28.1481	-7.9972	-23.2107	-8.9344
**1**	-8.6167	-48.5433	-27.4762	-7.8290	-29.6131	-8.6167
**1**	-7.9467	-42.3455	-25.6510	-9.6144	-5.7398	-7.9467
**1**	-7.8165	-48.3387	-18.7807	-9.1360	-19.5343	-7.8165
**1**	-7.7813	-39.4436	-18.4346	-8.5048	-13.8324	-7.7813
**1**	-7.3651	-49.1032	-20.0311	-8.1013	-15.2034	-7.3651
**1**	-7.2839	-47.3109	-21.0416	-8.2842	-16.6757	-7.2839
**2**	-8.1144	-50.7917	-25.3729	-9.3513	-26.1691	-8.1144
**2**	-8.0771	-39.1846	-19.6832	-9.5829	-7.5630	-8.0771
**2**	-7.4749	-51.0584	-17.8084	-8.7937	-29.0829	-7.4749
**2**	-7.4748	-51.0620	-32.6211	-8.6946	-29.0796	-7.4748
**2**	-7.3055	-47.1204	-18.6753	-8.4466	-17.7960	-7.3055
**2**	-7.1458	-43.6664	-22.3148	-8.8797	-13.1793	-7.1458
**2**	-7.0571	-50.5811	-21.4530	-8.5875	-17.2021	-7.0571

**2**	-6.9682	-48.3567	-33.7238	-11.2191	-12.2058	-6.9682
**2**	-6.9432	-45.4379	-22.9673	-9.5667	-3.6194	-6.9432
**2**	-6.9389	-45.7644	-21.3027	-9.6643	-14.8018	-6.9389
**3**	-10.5878	-18.9457	-31.4863	-10.1438	7.0218	-10.5878
**3**	-9.5626	-42.2045	-20.2995	-8.6613	-19.2315	-9.5626
**3**	-9.3506	-38.9212	-17.5137	-8.2308	-24.7261	-9.3506
**3**	-9.3231	-42.5655	-26.5405	-9.0895	-25.1933	-9.3231
**3**	-9.0031	-45.2524	-38.7712	-11.1381	-28.2734	-9.0031
**3**	-8.8932	-38.5696	-30.9905	-9.4520	-11.4654	-8.8932
**3**	-8.4852	-32.0124	-21.0520	-7.9074	-2.9656	-8.4852
**3**	-7.9748	-49.6609	-24.3158	-8.8533	-20.5586	-7.9748
**3**	-7.7630	-45.5956	-24.9819	-9.4224	-17.9371	-7.7630
**3**	-7.6503	-29.1979	-25.3221	-8.5440	-3.4975	-7.6503
**4**	-9.6325	-39.6040	-30.8450	-9.4190	-18.6845	-9.6325
**4**	-8.8963	-38.9960	-29.0621	-8.7822	-22.9874	-8.8963
**4**	-8.2922	-33.7673	-22.1036	-9.7895	13.6997	-8.2922
**4**	-8.2484	-45.4510	-24.6903	-8.9422	-12.4736	-8.2484
**4**	-8.2292	-53.1790	-24.0905	-9.8634	-17.0082	-8.2292
**4**	-8.2195	-37.3738	-19.3905	-8.6728	-17.8328	-8.2195
**4**	-8.1213	-45.5405	-27.4410	-9.0916	-8.7742	-8.1213
**4**	-8.0147	-51.0825	-24.6135	-9.6065	-13.9583	-8.0147
**4**	-8.0053	-42.8437	-23.6788	-9.1258	-4.9787	-8.0053
**4**	-7.8601	-44.9619	-29.9113	-9.6261	-14.8783	-7.8601
**5**	-8.9024	6.7965	-21.2115	-8.9830	6.9895	-8.9024
**5**	-8.6758	-36.1406	-31.6933	-8.8887	-15.4207	-8.6758
**5**	-7.7182	-35.3433	-21.9398	-8.5505	-14.9504	-7.7182
**5**	-7.7043	-43.6640	-24.9165	-8.8838	-18.8552	-7.7043
**5**	-7.6287	-40.6700	-19.4641	-7.9739	-13.8240	-7.6287
**5**	-7.5847	-38.9270	-25.1764	-8.7668	-25.6075	-7.5847
**5**	-7.1810	-38.1658	-25.1428	-8.2855	-25.1012	-7.1810
**5**	-7.1136	-38.9045	-25.9766	-9.9756	-16.3470	-7.1136
**5**	-7.1081	-47.6091	-25.5370	-8.9308	-15.3825	-7.1081
**5**	-6.8486	-43.6880	-15.8370	-8.5546	-22.7091	-6.8486
**6**	-10.2206	-18.3512	-20.8563	-8.0137	-29.2764	-10.2206
**6**	-10.1788	-14.6657	-19.3076	-8.8039	-25.0414	-10.1788
**6**	-10.1258	-27.5984	-19.4824	-8.3457	-20.2695	-10.1258
**6**	-10.1151	-20.2532	-23.2904	-7.7866	-27.3793	-10.1151
**6**	-9.9975	-7.3810	-16.8774	-9.4954	51.6183	-9.9975
**6**	-9.5889	-17.8983	-20.4954	-7.9549	-20.1281	-9.5889
**6**	-9.4249	-19.6337	-23.0076	-8.3819	-19.3121	-9.4249
**6**	-9.3384	-2.3643	-24.5323	-9.1810	-11.1050	-9.3384
**6**	-9.2658	-17.5509	-23.8552	-11.6104	-14.9267	-9.2658
**6**	-9.1761	-14.7681	-24.0717	-7.6903	-17.0571	-9.1761
**7**	-10.6856	17.3195	-27.4333	-8.9435	-18.4120	-10.6856
**7**	-10.6822	34.8136	-23.6160	-8.6588	10.1869	-10.6822
**7**	-10.5591	21.4632	-36.2113	-8.8634	-14.0017	-10.5591

**7**	-10.3860	28.1219	-22.6676	-8.7134	9.1169	-10.3860
**7**	-10.2416	14.3181	-32.8900	-9.6292	-4.3309	-10.2416
**7**	-10.1546	24.4966	-36.0244	-11.1829	-13.7041	-10.1546
**7**	-10.0031	17.4580	-33.5266	-8.8884	8.2728	-10.0031
**7**	-9.9702	16.7595	-28.3186	-9.1978	-14.5834	-9.9702
**7**	-9.6948	9.7073	-19.7065	-9.4492	-16.3879	-9.6948
**7**	-9.3994	20.0443	-27.0758	-9.0775	-23.4827	-9.3994
**8**	-11.7580	40.0966	-29.1720	-7.8238	-1.2025	-11.7580
**8**	-11.6980	42.5667	-31.3180	-7.8524	-13.9896	-11.6980
**8**	-11.5612	41.7446	-28.0426	-9.0847	-6.9450	-11.5612
**8**	-10.9653	38.1951	-32.7198	-8.4754	-14.6462	-10.9653
**8**	-10.4682	41.3565	-26.8452	-8.1665	-15.6221	-10.4682
**8**	-10.3436	41.5951	-28.2643	-9.8387	3.7742	-10.3436
**8**	-10.2528	37.3372	-27.3794	-9.1686	-8.8902	-10.2528
**8**	-10.0720	59.8457	-28.9742	-7.6376	17.5465	-10.0720
**8**	-9.9405	42.9009	-14.2820	-8.5186	-21.5935	-9.9405
**8**	-9.5348	49.6718	-24.1953	-8.9034	13.8869	-9.5348
**9**	-8.2959	-51.1083	-17.5034	-7.6183	-21.9051	-8.2959
**9**	-8.2330	-44.7898	-15.3641	-7.4359	-3.5915	-8.2330
**9**	-8.2169	-39.4007	-20.7057	-10.2535	-6.6024	-8.2169
**9**	-8.1991	-57.5728	-13.9010	-9.7210	-15.5623	-8.1991
**9**	-8.1117	-52.1246	-21.2162	-7.5788	-25.9125	-8.1117
**9**	-7.8609	-54.2362	-17.8920	-9.9149	-24.2803	-7.8609
**9**	-7.8525	-47.1343	-23.1347	-7.8417	-21.8377	-7.8525
**9**	-7.8255	-54.9607	-28.4475	-10.5584	-28.8390	-7.8255
**9**	-7.7479	-53.5500	-18.6650	-9.8284	7.0213	-7.7479
**9**	-7.4265	-50.0922	-20.2700	-7.4052	-22.1531	-7.4265
**10**	-10.3013	-24.7614	-34.0070	-9.0498	-25.9820	-10.3013
**10**	-9.9413	-30.5955	-27.3615	-9.0646	-9.6791	-9.9413
**10**	-9.6243	-32.5099	-23.1356	-9.6876	-24.2546	-9.6243
**10**	-9.5507	-24.9688	-35.1714	-8.2626	-8.1118	-9.5507
**10**	-9.5285	-28.3704	-27.7602	-9.4189	-24.8285	-9.5285
**10**	-9.3648	-18.8765	-25.0676	-7.7735	-7.3949	-9.3648
**10**	-9.1062	-26.4878	-20.6396	-7.5317	-17.7843	-9.1062
**10**	-8.8971	-33.5893	-31.8139	-8.7369	-3.9934	-8.8971
**10**	-8.7971	-27.0707	-30.9790	-8.4944	-8.3836	-8.7971
**10**	-8.6063	-28.5688	-22.6237	-9.0123	-2.4891	-8.6063
**11**	-10.6562	-28.6143	-30.2226	-10.0730	-27.0208	-10.6562
**11**	-10.4639	-32.6675	-34.6832	-8.2885	-25.0037	-10.4639
**11**	-10.3029	-21.6914	-16.9705	-7.7928	-17.4857	-10.3029
**11**	-10.1306	-33.2248	-24.6398	-7.9922	-25.0305	-10.1306
**11**	-9.6599	-36.0355	-24.0793	-8.0476	-12.0786	-9.6599
**11**	-9.6021	-26.2137	-28.9337	-10.1389	-10.5818	-9.6021
**11**	-9.3068	-29.2502	-28.9363	-8.0988	22.9821	-9.3068
**11**	-9.2230	-10.1949	-22.7890	-8.5630	34.6320	-9.2230
**11**	-8.8541	-23.0532	-33.5683	-8.6387	10.6831	-8.8541

**11**	-8.6680	-4.6168	-18.9917	-7.9516	46.3885	-8.6680
**12**	-10.4594	-20.3199	-29.2042	-7.5386	-16.1867	-10.4594
**12**	-10.2066	-23.6985	-18.6446	-7.4939	-23.7270	-10.2066
**12**	-10.1864	-14.3867	-33.4577	-8.2143	-15.5228	-10.1864
**12**	-10.1074	5.0938	-30.5640	-8.6197	-16.7069	-10.1074
**12**	-9.9647	-20.5903	-42.4968	-10.2617	-15.0006	-9.9647
**12**	-9.6585	-8.4244	-38.5863	-7.5063	7.8775	-9.6585
**12**	-9.5658	6.0184	-25.9751	-9.1940	4.5910	-9.5658
**12**	-9.2844	-2.6998	-25.6502	-7.7236	10.8821	-9.2844
**12**	-9.2085	-14.7410	-29.3802	-9.5225	8.3429	-9.2085
**12**	-9.1918	-7.4412	-38.2670	-9.2751	-0.6775	-9.1918
**13**	-10.2202	-4.9189	-30.9119	-7.5871	-7.2494	-10.2202
**13**	-9.3077	-6.8987	-23.2635	-7.0652	13.2543	-9.3077
**13**	-9.2865	-15.4676	-22.0314	-7.0299	-8.6863	-9.2865
**13**	-9.1559	-6.8228	-27.6958	-7.1761	13.0956	-9.1559
**13**	-9.1208	-10.8410	-30.6928	-8.3212	-23.0170	-9.1208
**13**	-9.0966	-12.1160	-27.5214	-6.8693	6.5325	-9.0966
**13**	-8.7387	-20.5482	-18.4107	-7.0563	-17.3811	-8.7387
**13**	-8.6515	-9.4790	-38.0645	-8.9112	-5.0422	-8.6515
**13**	-8.5797	-1.5751	-22.5957	-8.9429	11.9075	-8.5797
**13**	-8.4716	-12.0293	-12.9102	-6.8801	70.4553	-8.4716
**14**	-10.9063	-26.4287	-23.8714	-8.2233	-28.9128	-10.9063
**14**	-10.7691	-17.6250	-31.2709	-10.0769	-24.6988	-10.7691
**14**	-10.6037	-15.7013	-34.5166	-10.0945	-25.2533	-10.6037
**14**	-10.5381	-13.1540	-36.5153	-11.1021	-18.3544	-10.5381
**14**	-10.3331	-19.0223	-45.5135	-8.8616	-10.1119	-10.3331
**14**	-10.0489	-23.5580	-31.9473	-10.9461	-7.1689	-10.0489
**14**	-9.8798	-13.3115	-30.7720	-9.6762	-11.8429	-9.8798
**14**	-9.6127	-18.0083	-34.3563	-7.8220	-12.8450	-9.6127
**14**	-9.5808	-15.6977	-27.5591	-9.4734	-18.5116	-9.5808
**14**	-9.5779	-18.1302	-29.9098	-7.2881	-16.4635	-9.5779
**15**	-10.9934	-31.4326	-35.4511	-8.8395	-23.6716	-10.9934
**15**	-10.6488	-27.4007	-32.7834	-8.0095	-27.4353	-10.6488
**15**	-10.0166	-26.9978	-25.3344	-9.0240	26.9721	-10.0166
**15**	-9.9849	-27.5636	-28.3650	-12.2052	-16.1783	-9.9849
**15**	-9.9638	-16.6293	-30.1041	-7.5196	-19.0977	-9.9638
**15**	-9.7594	-29.4214	-22.8583	-7.2011	-17.4539	-9.7594
**15**	-9.4975	-23.1519	-28.0405	-10.2808	-3.8178	-9.4975
**15**	-9.3298	-28.7265	-31.8064	-7.0323	0.0171	-9.3298
**15**	-9.0968	-16.9413	-32.0815	-8.8226	12.2085	-9.0968
**15**	-9.0501	-13.6634	-25.6612	-8.1658	40.9827	-9.0501
**16**	-10.6166	-25.9804	-37.6326	-9.0688	-12.3348	-10.6166
**16**	-10.1895	-33.9157	-38.8749	-9.7439	-33.1806	-10.1895
**16**	-9.9181	-24.0170	-30.0355	-8.0552	-18.1332	-9.9181
**16**	-9.4597	-27.6432	-26.4770	-7.9959	-22.0286	-9.4597
**16**	-9.4071	-21.7246	-23.3507	-8.5213	6.5402	-9.4071

**16**	-9.3512	-15.3919	-16.8763	-8.4363	-13.6957	-9.3512
**16**	-9.2732	-20.0033	-22.0248	-8.1488	-13.4452	-9.2732
**16**	-9.0266	-32.3940	-20.4978	-8.0440	-19.4790	-9.0266
**16**	-8.8986	-29.4664	-20.0783	-8.3610	-20.4193	-8.8986
**16**	-8.8841	-17.0353	-29.4641	-8.4029	13.5789	-8.8841
**17**	-9.1257	-34.3286	-25.1491	-8.5305	-11.0866	-9.1257
**17**	-9.0535	-32.0351	-38.1564	-11.2084	-25.2556	-9.0535
**17**	-8.9872	-35.3704	-26.4558	-10.7152	-21.7535	-8.9872
**17**	-8.8453	-35.0594	-34.8193	-9.1521	-31.2273	-8.8453
**17**	-8.8197	-25.2356	-35.4336	-9.2441	-20.6142	-8.8197
**17**	-8.7712	-29.3959	-29.5622	-9.3473	-14.1821	-8.7712
**17**	-8.7319	-15.5646	-29.5904	-10.1696	-0.7843	-8.7319
**17**	-8.6685	-28.9091	-39.3241	-10.0620	16.0342	-8.6685
**17**	-8.4218	-35.4225	-27.6388	-9.1644	-14.6738	-8.4218
**17**	-8.3903	-29.5489	-23.4110	-9.1690	-25.7979	-8.3903
**18**	-10.9132	-28.8382	-29.2158	-9.0911	-23.4994	-10.9132
**18**	-10.7645	18.6252	-29.5605	-9.5664	40.6146	-10.7645
**18**	-10.7488	-15.5522	-36.4968	-8.7999	4.2133	-10.7488
**18**	-10.3054	-31.6483	-16.6813	-7.0013	-9.1165	-10.3054
**18**	-10.2512	-31.4450	-31.6273	-8.0424	-10.4274	-10.2512
**18**	-10.0207	-29.2022	-21.0523	-7.8890	-17.7643	-10.0207
**18**	-9.7634	-25.7046	-20.5480	-8.4192	-18.0784	-9.7634
**18**	-9.6922	-24.0033	-20.8684	-8.0622	-4.4832	-9.6922
**18**	-9.6305	-20.5643	-27.1845	-8.6961	-8.8487	-9.6305
**18**	-9.5857	-28.5568	-23.2858	-7.4273	-7.0697	-9.5857
**19**	-10.5465	-21.4047	-19.6544	-7.3632	-24.8632	-10.5465
**19**	-10.3235	-15.3884	-24.6740	-9.1857	-11.7835	-10.3235
**19**	-10.3089	-19.0376	-38.2177	-7.2021	-8.0437	-10.3089
**19**	-10.0874	-26.4603	-24.7009	-9.7398	-24.5458	-10.0874
**19**	-10.0660	-22.2702	-31.1315	-7.5225	-16.6384	-10.0660
**19**	-9.5875	-18.0126	-28.1411	-12.1383	-9.5315	-9.5875
**19**	-9.4035	-11.7819	-24.8886	-7.6191	18.9914	-9.4035
**19**	-9.1588	-10.3560	-33.1363	-7.5553	-2.3734	-9.1588
**19**	-8.9529	-12.5664	-22.2521	-9.1346	-6.0371	-8.9529
**19**	-8.7488	-17.6764	-25.6652	-7.4175	-19.3158	-8.7488
**20**	-9.8997	-23.2073	-38.4303	-10.1196	-22.4494	-9.8997
**20**	-9.8335	-31.9465	-21.3469	-8.9687	-20.5395	-9.8335
**20**	-9.7847	-35.4399	-25.3276	-8.2725	-8.5359	-9.7847
**20**	-9.7056	-35.4825	-24.4275	-9.2452	-15.0068	-9.7056
**20**	-9.6922	-36.1153	-25.5113	-9.4894	3.0631	-9.6922
**20**	-9.5823	-33.9328	-29.4496	-8.2530	-29.6905	-9.5823
**20**	-9.1916	-2.2633	-26.7092	-8.6151	-8.2384	-9.1916
**20**	-9.0225	-7.9301	-28.2626	-9.9367	11.8937	-9.0225
**20**	-9.0159	5.6404	-31.1558	-8.9310	7.7178	-9.0159
**20**	-8.9593	-27.5656	-29.4755	-8.6201	-9.2410	-8.9593
**21**	-10.3466	-74.5313	-26.5733	-7.8552	1.1872	-10.3466

**21**	-10.1784	-65.6338	-29.5907	-7.8150	-9.4752	-10.1784
**21**	-10.1400	-73.8693	-17.7255	-7.2412	-13.5851	-10.1400
**21**	-9.8201	-71.4550	-26.7174	-10.4892	-16.9526	-9.8201
**21**	-9.6702	-75.3307	-28.0195	-8.3123	-13.0244	-9.6702
**21**	-9.6097	-74.1524	-22.4535	-9.4349	-25.2809	-9.6097
**21**	-9.2538	-59.2648	-20.9093	-7.6330	-1.7428	-9.2538
**21**	-9.0444	-76.8688	-21.0809	-10.5278	-12.8067	-9.0444
**21**	-8.8755	-70.9223	-34.8721	-9.2474	11.8864	-8.8755
**21**	-8.8673	-74.1085	-22.1266	-7.0535	-15.2793	-8.8673
**22**	-6.5829	-45.2019	-24.1186	-11.0163	-21.3166	-6.5829
**22**	-6.2949	-39.1498	-29.4145	-9.8431	-18.5776	-6.2949
**22**	-6.2614	-41.2986	-24.1521	-10.3302	-22.6898	-6.2614
**22**	-6.1403	-39.8166	-14.3284	-9.6674	-8.9823	-6.1403
**22**	-6.0049	-35.6872	-23.6061	-11.1338	-15.8810	-6.0049
**22**	-5.8925	-36.0508	-22.5130	-9.6932	-9.9161	-5.8925
**22**	-5.7120	-38.8513	-17.1319	-9.4724	-16.8881	-5.7120
**22**	-5.6929	-39.0247	-21.8272	-9.2551	-5.0394	-5.6929
**22**	-5.5749	-28.8211	-19.6026	-10.2740	7.1840	-5.5749
**22**	-5.4436	-36.3360	-15.2979	-9.1857	5.5821	-5.4436
**23**	-7.9453	-32.5525	-29.2791	-10.1585	-21.6102	-7.9453
**23**	-7.8743	-38.8735	-21.0039	-9.1751	-6.9734	-7.8743
**23**	-7.8520	-47.6927	-25.3313	-10.1763	-19.4091	-7.8520
**23**	-7.7916	-44.5202	-19.7182	-10.0078	-6.8589	-7.7916
**23**	-7.6041	-38.2136	-18.8017	-9.3837	-10.6959	-7.6041
**23**	-7.1665	-25.0813	-32.5311	-10.5645	-7.9834	-7.1665
**23**	-6.8596	-41.3360	-21.4407	-8.9792	-20.0473	-6.8596
**23**	-6.8152	-31.4049	-30.8381	-10.5613	-17.1668	-6.8152
**23**	-6.5691	-36.5193	-24.6843	-9.2312	-15.9135	-6.5691
**23**	-6.5479	-37.8798	-20.8198	-9.5899	-3.9509	-6.5479
**24**	-8.2257	-37.3468	-21.3838	-8.9584	-10.4321	-8.2257
**24**	-7.9658	-46.7672	-25.2297	-10.1278	-16.4499	-7.9658
**24**	-7.9354	-37.1187	-21.0829	-9.4778	-3.1675	-7.9354
**24**	-7.8835	-31.9361	-29.3573	-10.4310	-21.6342	-7.8835
**24**	-7.8481	-43.8213	-19.4112	-10.0983	-3.4339	-7.8481
**24**	-7.6514	-43.1165	-25.8889	-9.1070	-14.5444	-7.6514
**24**	-7.1150	-40.0278	-20.7802	-8.9239	-18.7713	-7.1150
**24**	-7.1086	-23.9030	-27.9680	-9.5643	-8.3010	-7.1086
**24**	-6.9847	-40.2804	-19.9925	-10.2596	-19.3855	-6.9847
**24**	-6.8436	-30.8219	-30.7609	-10.6322	-17.2149	-6.8436
**25**	-9.8010	-0.2864	-25.3180	-8.9122	1.6786	-9.8010
**25**	-9.5036	48.0792	-27.6488	-8.3412	13.2863	-9.5036
**25**	-9.4333	-23.8532	-21.7237	-8.0486	-27.9726	-9.4333
**25**	-9.2903	-22.9323	-34.9015	-9.2897	-25.1079	-9.2903
**25**	-9.1747	-15.1628	-25.4830	-9.4153	-19.4816	-9.1747
**25**	-8.8014	-24.9057	-27.7189	-8.5957	-25.2209	-8.8014
**25**	-8.6933	-22.0933	-23.5723	-8.6786	-20.8280	-8.6933

**25**	-8.6250	-22.1995	-28.8001	-9.0128	-18.5694	-8.6250
**25**	-8.4322	-20.3900	-31.6146	-8.4239	-7.7097	-8.4322
**25**	-8.2039	-26.0399	-30.1917	-10.8389	-15.9871	-8.2039
**26**	-8.4188	-48.4585	-32.6319	-9.2593	-12.0207	-8.4188
**26**	-8.3322	-51.8575	-29.8560	-9.2534	-10.6359	-8.3322
**26**	-8.1416	-38.1972	-28.0455	-9.3176	-6.3073	-8.1416
**26**	-7.5334	-48.7150	-27.7979	-9.0693	-18.6052	-7.5334
**26**	-7.5296	-45.4954	-26.8853	-8.7970	-6.1994	-7.5296
**26**	-7.4183	-29.0929	-23.5025	-9.0357	14.3720	-7.4183
**26**	-7.1307	-45.2895	-18.2753	-9.5477	-11.6866	-7.1307
**26**	-6.8220	-52.5688	-20.7508	-8.6127	-26.3605	-6.8220
**26**	-6.8126	-52.7980	-23.9842	-8.9408	-24.1386	-6.8126
**26**	-6.7481	-48.3484	-24.7800	-8.7916	-21.9452	-6.7481

Table S6. Ten top scored docking positioning of the 5MAT co-crystallized ligand (**36**) and of the related analogues (**27-111**) at the 5MAT PDB code (MOE software). The predicted ΔG value of each protein-ligand complex has been reported, as calculated in terms of final scoring function (S, as Kcal/mol).

**Compound**	**S**	**E_conf**	**E_place**	**E_score1**	**E_refine**	**E_score2**
**27**	-13.8512	19.8990	-43.1961	-11.7123	-33.0118	-13.8512
**27**	-13.2111	14.3341	-47.6496	-13.2845	-49.7220	-13.2111
**27**	-13.0939	22.3950	-38.0595	-10.6986	-45.1665	-13.0939
**27**	-13.0310	23.0860	-40.7726	-9.5778	-36.0707	-13.0310
**27**	-12.8640	26.9085	-32.5700	-9.9519	-39.0048	-12.8640
**27**	-12.8452	22.9183	-40.1373	-12.6830	-11.0974	-12.8452
**27**	-12.7959	23.2919	-30.5397	-9.4822	-12.0560	-12.7959
**27**	-12.4992	15.0494	-41.6590	-12.4822	-46.9961	-12.4992
**27**	-12.4291	20.3228	-25.6230	-10.3228	-12.7027	-12.4291
**27**	-12.2688	47.6150	-35.3863	-9.5620	-19.7380	-12.2688
**28**	-13.7933	20.5136	-36.3695	-11.9576	-33.1795	-13.7933
**28**	-13.5993	23.7646	-34.3127	-10.2732	-31.9539	-13.5993
**28**	-13.2294	21.3097	-38.4485	-11.0205	-43.3686	-13.2294
**28**	-12.7786	16.0013	-41.6782	-10.2130	-50.6158	-12.7786
**28**	-12.6065	43.0109	-34.7978	-10.5834	-34.3015	-12.6065
**28**	-12.5922	27.5169	-36.9508	-10.9090	-22.2423	-12.5922
**28**	-12.4279	20.9377	-38.9122	-9.9867	-41.8661	-12.4279
**28**	-11.8322	21.6933	-34.8426	-12.5748	-38.1605	-11.8322
**28**	-11.1703	28.1473	-39.7805	-10.1053	-36.8209	-11.1703
**28**	-11.1669	28.1785	-39.2714	-10.0919	-36.8644	-11.1669
**29**	-13.5610	28.9188	-35.3942	-9.8132	-25.6281	-13.5610
**29**	-13.2728	21.7737	-37.3687	-9.8373	-33.9999	-13.2728
**29**	-13.1734	25.6240	-40.6709	-10.1765	-26.8896	-13.1734
**29**	-12.9733	15.2370	-47.3552	-12.5202	-37.1424	-12.9733
**29**	-12.8407	22.0143	-37.1096	-16.8381	-39.3378	-12.8407
**29**	-12.8074	17.1666	-36.4757	-10.9632	-20.0087	-12.8074
**29**	-12.7987	24.3451	-35.8327	-12.9319	-26.8087	-12.7987

**29**	-12.6550	20.4753	-50.8925	-13.1994	-26.8969	-12.6550
**29**	-12.5750	27.1464	-40.3263	-10.8245	-23.8529	-12.5750
**29**	-12.4757	22.0879	-38.8985	-10.7238	-26.6940	-12.4757
**30**	-12.8481	18.7634	-30.4731	-10.3969	-34.6136	-12.8481
**30**	-12.7308	30.2546	-42.4051	-10.7245	-29.0960	-12.7308
**30**	-12.7022	19.0084	-40.0123	-13.0794	-33.2987	-12.7022
**30**	-12.2533	12.1384	-34.0683	-10.2952	-49.3284	-12.2533
**30**	-11.4523	32.6988	-33.5936	-10.9802	-26.9011	-11.4523
**30**	-11.4246	14.3461	-45.0312	-10.6579	-43.0877	-11.4246
**30**	-11.3941	14.6740	-40.7159	-11.1659	-44.4056	-11.3941
**30**	-11.3395	37.7396	-29.8846	-10.4495	-26.8998	-11.3395
**30**	-11.2993	36.7691	-41.1554	-12.3046	-26.6708	-11.2993
**30**	-10.5868	44.5546	-33.7568	-10.1709	4.2797	-10.5868
**31**	-13.7665	-34.2014	-44.8527	-8.5284	-29.5514	-13.7665
**31**	-13.7433	-33.5328	-38.7338	-9.0976	-35.9491	-13.7433
**31**	-13.6748	-30.6335	-38.1154	-10.0175	-35.7954	-13.6748
**31**	-13.6614	-36.6577	-44.6026	-8.9577	-32.5031	-13.6614
**31**	-13.5418	-38.6822	-44.8596	-7.7302	-41.2775	-13.5418
**31**	-13.4707	-17.3477	-42.9367	-8.0465	-15.7683	-13.4707
**31**	-13.4185	-2.0717	-33.6429	-9.6503	-11.8363	-13.4185
**31**	-13.1154	-35.0731	-38.6715	-9.5445	-14.7303	-13.1154
**31**	-12.4509	-29.4201	-44.5714	-10.5895	-32.7545	-12.4509
**31**	-11.9655	-29.8461	-43.1771	-10.4825	-7.6319	-11.9655
**32**	-14.0006	-44.5943	-35.9708	-7.3518	-52.2306	-14.0006
**32**	-13.6284	-36.2096	-36.5015	-8.9155	-43.4281	-13.6284
**32**	-13.4924	-39.8865	-33.2457	-6.8563	-45.5756	-13.4924
**32**	-12.9146	-13.4036	-35.5605	-8.9820	-25.9612	-12.9146
**32**	-12.5734	-23.2643	-36.1002	-11.5443	-14.4709	-12.5734
**32**	-12.4554	-25.4020	-32.1846	-6.7077	-30.1256	-12.4554
**32**	-12.3419	-19.4887	-44.4752	-10.6278	-38.6995	-12.3419
**32**	-12.3198	-21.7785	-46.9182	-9.1131	-24.7787	-12.3198
**32**	-12.2493	-32.8255	-38.4893	-7.6205	-45.2202	-12.2493
**32**	-12.0440	20.5016	-26.4206	-6.7671	34.0724	-12.0440
**33**	-14.0138	-40.8196	-34.4410	-10.6657	-39.5058	-14.0138
**33**	-13.7676	-38.6884	-44.9758	-12.5074	-32.7214	-13.7676
**33**	-13.6296	-41.4731	-36.6718	-9.5051	-40.5945	-13.6296
**33**	-13.3244	-5.1951	-48.3929	-14.1228	-18.7416	-13.3244
**33**	-13.0774	-28.4142	-40.9996	-12.7552	-23.4711	-13.0774
**33**	-13.0024	-31.3044	-45.4761	-10.8068	-29.5890	-13.0024
**33**	-12.9963	-22.3188	-34.6624	-7.7080	-8.4716	-12.9963
**33**	-12.9222	-32.3423	-49.9409	-9.0198	-38.5617	-12.9222
**33**	-12.7077	-33.7822	-33.3698	-7.8915	-9.5056	-12.7077
**33**	-12.5227	-32.4870	-46.5041	-8.9804	-24.6842	-12.5227
**34**	-13.7640	-17.6397	-50.3886	-11.3216	-37.9434	-13.7640
**34**	-13.6336	-17.4945	-40.2624	-9.0534	-40.7014	-13.6336
**34**	-13.4175	-26.1063	-46.5903	-13.5878	-48.8494	-13.4175

**34**	-13.3141	-14.5793	-44.2070	-8.5712	-15.7644	-13.3141
**34**	-12.9640	-21.1294	-42.4607	-9.3006	-15.5032	-12.9640
**34**	-12.3468	-7.6987	-45.6512	-9.1846	-33.8373	-12.3468
**34**	-12.3335	4.0365	-38.9412	-7.7351	-14.6884	-12.3335
**34**	-12.1722	-24.7566	-46.6333	-7.4150	-47.6785	-12.1722
**34**	-11.9837	2.5043	-38.5293	-12.6012	-20.6687	-11.9837
**34**	-11.7222	3.0986	-36.0657	-10.2526	13.6257	-11.7222
**35**	-13.9123	-30.6940	-52.1551	-9.0691	-52.0915	-13.9123
**35**	-13.6736	-30.8008	-41.9155	-9.8979	-52.2860	-13.6736
**35**	-13.6055	-29.9868	-47.2390	-11.4017	-52.8482	-13.6055
**35**	-13.5708	-6.0863	-48.9925	-12.9954	-34.4562	-13.5708
**35**	-13.3191	-23.6444	-50.0908	-11.9080	-46.4736	-13.3191
**35**	-12.8048	-14.8350	-41.4670	-10.5112	-26.0041	-12.8048
**35**	-12.7660	3.8861	-35.3415	-8.8830	-30.8438	-12.7660
**35**	-12.6380	2.7028	-42.2043	-9.9033	-35.4931	-12.6380
**35**	-12.4982	-22.0239	-54.2046	-8.9687	-47.0820	-12.4982
**35**	-12.3641	-1.9847	-48.3263	-11.5155	-14.0503	-12.3641
**36**	-13.6026	-20.6210	-53.7004	-15.1157	-51.4327	-13.6026
**36**	-13.5889	-10.2977	-35.2132	-7.9602	-46.4230	-13.5889
**36**	-13.0428	-14.6524	-37.4798	-7.1726	-44.4309	-13.0428
**36**	-12.6881	-1.1768	-42.7482	-8.0616	-32.6498	-12.6881
**36**	-12.5862	-8.4642	-34.2406	-9.7077	-45.9022	-12.5862
**36**	-12.4366	16.4665	-45.3031	-9.1396	-8.9867	-12.4366
**36**	-12.1411	-19.3406	-55.5793	-12.9646	-48.8317	-12.1411
**36**	-11.9847	-4.0796	-37.5540	-9.6170	-14.5390	-11.9847
**36**	-11.7261	1.3808	-43.5532	-12.5893	-29.4314	-11.7261
**36**	-11.5957	-11.7872	-40.2544	-7.2610	-42.6412	-11.5957
**37**	-13.3214	-33.5584	-38.1982	-10.1945	-53.8128	-13.3214
**37**	-13.1623	-32.9282	-44.0711	-10.3494	-54.4239	-13.1623
**37**	-12.9758	-33.1496	-51.1265	-11.4095	-53.0920	-12.9758
**37**	-12.5409	-11.0960	-39.6599	-9.8869	-37.7167	-12.5409
**37**	-12.4724	-28.6984	-44.1670	-11.8351	-49.5563	-12.4724
**37**	-12.3752	-4.2760	-39.9129	-9.8124	-28.6775	-12.3752
**37**	-12.0415	-0.1026	-37.2286	-9.3720	-44.1187	-12.0415
**37**	-11.9801	-29.9448	-41.7596	-12.2597	-53.7466	-11.9801
**37**	-11.9653	-7.7805	-35.1027	-8.8405	-21.4743	-11.9653
**37**	-11.7270	-13.1977	-39.6514	-10.7935	-23.8043	-11.7270
**38**	-13.3981	4.9575	-40.9022	-10.5418	-42.4481	-13.3981
**38**	-13.1662	-3.0783	-38.3598	-11.5197	-50.6553	-13.1662
**38**	-12.6364	49.0696	-36.0929	-10.5468	-17.3932	-12.6364
**38**	-12.4161	-0.8939	-45.8682	-15.5214	-28.3246	-12.4161
**38**	-12.3289	0.6280	-42.2444	-7.6989	-49.0106	-12.3289
**38**	-11.9959	-1.4598	-38.1605	-6.7193	-39.7205	-11.9959
**38**	-11.5525	-3.1377	-35.3100	-10.3129	-30.2601	-11.5525
**38**	-11.3778	30.8752	-40.2948	-7.0241	-15.6522	-11.3778
**38**	-11.1596	16.1104	-34.7962	-9.0941	-29.0040	-11.1596

**38**	-10.8567	27.7468	-32.3571	-7.3581	11.8447	-10.8567
**39**	-12.5896	1.2284	-40.8510	-10.4871	-43.7443	-12.5896
**39**	-12.3767	-1.0193	-22.7414	-8.2605	-36.5567	-12.3767
**39**	-12.2847	10.6079	-27.8917	-10.7867	-30.6461	-12.2847
**39**	-12.0136	-6.5238	-46.5636	-9.7272	-50.5142	-12.0136
**39**	-11.9907	1.6301	-32.0365	-9.6012	-39.8792	-11.9907
**39**	-11.7289	0.1328	-47.9350	-13.1341	-44.3061	-11.7289
**39**	-11.3853	-4.1783	-47.0634	-12.6566	-42.2848	-11.3853
**39**	-11.3514	7.3164	-28.8458	-8.7414	-40.5488	-11.3514
**39**	-11.2344	18.2430	-29.3209	-10.1047	-16.5838	-11.2344
**39**	-11.2288	-4.3136	-40.0282	-9.3302	-50.0569	-11.2288
**40**	-14.0032	-0.7707	-44.3738	-9.0391	-23.8177	-14.0032
**40**	-13.7801	-10.6646	-43.5544	-8.6926	-37.1141	-13.7801
**40**	-13.4991	-10.0892	-28.7787	-10.8442	-34.0532	-13.4991
**40**	-13.2724	-12.3719	-54.1408	-10.1707	-41.0531	-13.2724
**40**	-12.9570	-20.3420	-49.5140	-10.9151	-52.6283	-12.9570
**40**	-12.7464	36.5059	-39.6774	-11.0504	-8.2966	-12.7464
**40**	-12.6200	-11.5706	-38.5458	-9.1477	-9.6088	-12.6200
**40**	-12.5950	-14.9929	-41.2096	-11.9880	-12.1407	-12.5950
**40**	-12.2539	-16.4771	-31.9248	-8.7512	-30.8365	-12.2539
**40**	-12.0013	52.5555	-39.3035	-9.7990	16.3370	-12.0013
**41**	-13.5511	-13.2100	-31.4676	-9.9335	-33.4003	-13.5511
**41**	-13.4789	-9.6950	-29.7862	-9.7098	-20.8234	-13.4789
**41**	-13.3264	-3.9835	-32.6615	-11.5881	-24.4862	-13.3264
**41**	-12.9364	-18.8923	-36.7402	-12.0232	-20.1154	-12.9364
**41**	-12.8886	-11.5050	-40.0701	-12.3425	-24.4412	-12.8886
**41**	-12.7519	-12.1727	-37.1982	-11.6345	-34.0868	-12.7519
**41**	-12.4783	-18.4015	-37.1894	-11.1595	-45.7812	-12.4783
**41**	-12.2522	-16.9492	-40.5469	-13.8644	-38.2157	-12.2522
**41**	-12.1766	-11.4505	-38.4546	-9.9999	-24.1538	-12.1766
**41**	-12.0101	-15.5218	-41.4840	-9.8669	-42.8378	-12.0101
**42**	-12.7192	-17.2847	-30.2507	-10.7294	-35.1065	-12.7192
**42**	-12.5080	-4.1715	-27.4738	-11.2150	-28.7673	-12.5080
**42**	-12.4262	-21.7582	-46.0773	-11.0130	-42.1385	-12.4262
**42**	-12.2180	-15.7704	-26.7758	-10.6742	-43.9343	-12.2180
**42**	-12.1684	-11.4512	-34.4844	-9.0041	-37.4335	-12.1684
**42**	-11.7978	-19.1810	-35.3161	-10.5006	-41.8872	-11.7978
**42**	-11.5818	-12.6694	-33.7600	-8.7547	-36.6595	-11.5818
**42**	-10.9494	6.0429	-33.1513	-10.2307	-20.5989	-10.9494
**42**	-10.0181	45.4687	-25.7773	-8.5781	14.4853	-10.0181
**42**	-9.9754	12.2522	-31.5069	-8.5314	20.5793	-9.9754
**43**	-11.4166	-26.6021	-36.2197	-10.0276	-32.7049	-11.4166
**43**	-11.4124	-14.3273	-28.5823	-11.1820	-38.9501	-11.4124
**43**	-10.9305	-30.3217	-17.2587	-9.2917	-47.8341	-10.9305
**43**	-10.8638	-15.0178	-38.7550	-9.4134	-40.0438	-10.8638
**43**	-10.4175	-13.7642	-34.0909	-11.2312	-2.7356	-10.4175

**43**	-10.3609	-18.7102	-29.5760	-10.2758	-36.1569	-10.3609
**43**	-10.2125	0.0244	-40.3844	-11.4903	-12.3740	-10.2125
**43**	-9.5179	54.7409	-40.8698	-9.6923	38.5640	-9.5179
**43**	-9.1669	10.3397	-24.2023	-9.1047	7.8721	-9.1669
**43**	-8.8457	-6.9708	-34.6096	-9.3115	-22.1128	-8.8457
**44**	-14.0347	-10.3253	-48.3179	-9.6834	-39.1517	-14.0347
**44**	-13.7515	-7.8029	-38.7942	-7.4995	-41.2130	-13.7515
**44**	-13.6749	-16.2315	-43.2363	-7.7794	-35.4194	-13.6749
**44**	-13.6075	-11.5926	-28.4547	-7.6838	-42.5105	-13.6075
**44**	-13.3390	-19.8514	-40.8796	-7.4622	-53.5541	-13.3390
**44**	-13.3031	-18.9304	-37.4423	-11.4599	-46.0426	-13.3031
**44**	-13.2797	-10.7259	-34.9186	-8.3613	-34.7792	-13.2797
**44**	-13.1579	1.2545	-39.5438	-8.9292	-32.3028	-13.1579
**44**	-13.0512	-12.9617	-39.7622	-8.2985	-45.6125	-13.0512
**44**	-12.9888	13.6495	-50.4482	-13.0184	-17.1723	-12.9888
**45**	-13.5419	-22.1804	-46.8147	-13.1392	-46.6126	-13.5419
**45**	-13.2754	-23.9385	-58.3942	-14.0385	-50.3643	-13.2754
**45**	-12.7223	-19.3172	-47.5019	-13.0683	-45.1110	-12.7223
**45**	-12.4114	-17.5152	-55.0891	-13.6121	-38.1268	-12.4114
**45**	-12.2561	-11.7990	-33.6952	-10.4369	-23.3546	-12.2561
**45**	-12.1918	3.1134	-36.4149	-11.6763	-25.4335	-12.1918
**45**	-11.7655	-16.1135	-48.2648	-12.0478	-49.9158	-11.7655
**45**	-11.6072	-8.6433	-37.3349	-11.9411	-36.5438	-11.6072
**45**	-11.5378	-13.1703	-43.0023	-13.3392	-32.3785	-11.5378
**45**	-10.5546	23.7107	-35.9571	-11.2383	-10.6790	-10.5546
**46**	-13.2975	-21.8807	-53.9858	-13.4737	-41.6838	-13.2975
**46**	-13.1141	-26.1278	-38.8115	-9.6114	-49.0905	-13.1141
**46**	-12.5069	-21.4024	-44.1080	-9.3995	-44.1746	-12.5069
**46**	-12.3053	-15.6082	-42.5636	-12.6769	-27.3567	-12.3053
**46**	-11.7313	2.9387	-44.5826	-10.2653	-33.1775	-11.7313
**46**	-11.6241	-19.7851	-51.7702	-9.5639	-48.4754	-11.6241
**46**	-11.5807	-20.7137	-32.8293	-8.9874	-44.5154	-11.5807
**46**	-11.3473	3.0037	-39.7755	-10.7785	-24.1779	-11.3473
**46**	-11.3098	-8.5977	-48.0245	-10.3266	-37.5450	-11.3098
**46**	-11.0659	-13.0864	-43.3921	-9.1659	-44.6833	-11.0659
**47**	-13.3821	-26.1688	-43.8762	-11.0187	-52.1650	-13.3821
**47**	-13.1587	15.8179	-37.3004	-10.8657	-23.6752	-13.1587
**47**	-12.9265	-20.5126	-40.7651	-10.1491	-44.4801	-12.9265
**47**	-12.3689	11.1423	-44.0544	-11.7004	-26.6998	-12.3689
**47**	-12.1380	-13.2793	-50.9958	-11.6710	-24.3165	-12.1380
**47**	-12.0682	-8.7567	-36.4372	-11.7053	-41.2941	-12.0682
**47**	-12.0459	3.9665	-43.3981	-11.1840	-29.4694	-12.0459
**47**	-11.8545	2.1064	-36.1844	-10.5664	-35.8327	-11.8545
**47**	-11.8284	-3.4644	-36.8583	-8.6710	-29.7918	-11.8284
**47**	-11.6973	-24.1103	-43.5626	-12.6398	-45.7107	-11.6973
**48**	-13.1556	-25.1576	-40.2821	-9.8775	-50.5338	-13.1556

**48**	-12.9981	-22.8460	-37.1197	-10.5051	-42.4775	-12.9981
**48**	-12.6963	-20.0537	-46.8380	-11.4574	-46.6118	-12.6963
**48**	-12.2934	7.9757	-33.5355	-9.4604	-32.5550	-12.2934
**48**	-12.2479	-12.6121	-45.4599	-11.3318	-23.8446	-12.2479
**48**	-12.0097	0.3862	-44.2684	-8.7827	-27.2750	-12.0097
**48**	-11.6771	-17.4411	-51.7076	-10.7034	-47.2686	-11.6771
**48**	-11.5257	8.5557	-36.5825	-8.9421	-24.3018	-11.5257
**48**	-11.1817	16.6081	-26.0066	-9.5603	-6.4803	-11.1817
**48**	-10.9613	5.1344	-41.0108	-12.3577	-14.0886	-10.9613
**49**	-12.7933	-9.7074	-21.9221	-8.0964	-40.3094	-12.7933
**49**	-12.7521	-3.6040	-26.8038	-6.6101	-45.3566	-12.7521
**49**	-12.7319	1.5786	-34.3900	-6.2885	-35.0355	-12.7319
**49**	-12.1493	1.4253	-32.3483	-9.5886	-40.9433	-12.1493
**49**	-11.6715	0.8774	-39.0125	-11.3212	-43.5050	-11.6715
**49**	-11.6030	-2.6652	-32.2241	-7.3582	-43.4429	-11.6030
**49**	-11.3078	-8.2726	-39.3298	-10.8798	-49.7030	-11.3078
**49**	-10.8580	61.9777	-34.3435	-7.1691	-4.2323	-10.8580
**49**	-10.5481	32.7679	-37.9815	-10.4339	-2.6278	-10.5481
**49**	-10.3671	13.2774	-22.6225	-8.9539	-30.0671	-10.3671
**50**	-13.8392	-18.1751	-30.4046	-8.6235	-45.7203	-13.8392
**50**	-13.6661	-27.0785	-49.3611	-13.1180	-49.3480	-13.6661
**50**	-13.0172	28.3814	-38.5343	-10.0422	-14.8785	-13.0172
**50**	-12.9278	12.8394	-44.8245	-12.7765	-26.7866	-12.9278
**50**	-12.4505	-5.9417	-36.7734	-13.6128	-39.4881	-12.4505
**50**	-11.8804	11.3783	-37.1219	-9.1164	20.4044	-11.8804
**50**	-11.8518	-13.2095	-48.1415	-9.4808	-38.0760	-11.8518
**50**	-11.7239	5.5817	-34.6484	-9.1148	-29.0115	-11.7239
**50**	-11.7075	-10.2921	-37.2823	-13.3676	-0.5119	-11.7075
**50**	-11.5107	31.9435	-29.3437	-9.4338	29.9620	-11.5107
**51**	-13.7883	7.6509	-46.8929	-10.1293	-27.7185	-13.7883
**51**	-13.2380	-26.6568	-58.3904	-18.9680	-41.9473	-13.2380
**51**	-13.2136	9.9830	-36.0956	-9.5590	-3.3990	-13.2136
**51**	-13.1540	-26.7450	-54.9873	-13.0011	-43.2851	-13.1540
**51**	-13.1158	-26.7594	-47.2821	-9.5956	-37.9855	-13.1158
**51**	-13.0159	4.1678	-33.2303	-10.3080	-9.3978	-13.0159
**51**	-12.9518	-2.9867	-33.9749	-14.2296	-29.9339	-12.9518
**51**	-12.7569	-13.5937	-34.7216	-9.5843	-30.9802	-12.7569
**51**	-12.7455	-24.3533	-51.5597	-12.3108	-42.6786	-12.7455
**51**	-12.6641	-9.4377	-31.6432	-9.6153	-28.9647	-12.6641
**52**	-12.9806	-28.9372	-52.8971	-11.4308	-52.9894	-12.9806
**52**	-12.4618	4.3009	-44.4114	-12.1447	-37.9971	-12.4618
**52**	-12.3771	-23.6996	-45.6748	-13.0755	-47.3868	-12.3771
**52**	-12.3157	-8.4034	-37.3597	-11.2706	-25.9177	-12.3157
**52**	-11.8850	-11.4651	-31.8572	-8.6076	-32.3071	-11.8850
**52**	-11.8782	-6.7971	-55.1378	-13.0544	-44.9926	-11.8782
**52**	-11.8701	2.5317	-26.9295	-11.0123	-31.3625	-11.8701

**52**	-11.7362	-21.1704	-49.8952	-10.0522	-49.6559	-11.7362
**52**	-11.4888	0.1870	-32.7496	-11.6447	-29.6304	-11.4888
**52**	-11.4493	-10.6219	-29.3432	-13.2205	-41.0117	-11.4493
**53**	-12.1244	-16.3808	-33.7633	-9.7718	-45.8756	-12.1244
**53**	-11.7586	-24.9152	-37.9334	-13.3320	-37.7661	-11.7586
**53**	-11.6973	-36.7841	-50.3262	-15.1446	-45.0810	-11.6973
**53**	-11.6679	-15.0802	-31.6507	-10.7842	-30.4491	-11.6679
**53**	-11.5853	-29.8594	-27.4664	-9.5643	-40.5194	-11.5853
**53**	-11.5847	-6.3379	-28.5108	-10.5223	-22.9851	-11.5847
**53**	-11.5283	-14.6597	-37.9745	-9.5021	-38.4061	-11.5283
**53**	-11.1510	-5.1789	-34.4352	-12.8692	-31.8544	-11.1510
**53**	-10.8981	-13.4692	-41.4048	-10.2757	-18.1005	-10.8981
**53**	-10.6784	-22.2780	-44.1126	-10.4752	-45.7846	-10.6784
**54**	-14.6185	-26.0278	-34.5468	-8.7438	-49.7642	-14.6185
**54**	-14.2622	-27.0783	-53.4028	-14.8399	-50.4705	-14.2622
**54**	-13.6954	-21.1861	-34.2916	-8.4222	-46.1712	-13.6954
**54**	-13.6003	-28.7744	-50.8359	-11.7983	-50.4383	-13.6003
**54**	-13.5535	-15.6099	-36.1169	-13.3530	-43.7820	-13.5535
**54**	-13.4837	-11.6425	-38.2003	-10.0242	-16.9913	-13.4837
**54**	-13.4012	-23.8097	-50.3499	-10.3969	-56.0773	-13.4012
**54**	-13.2315	-12.9052	-43.5539	-8.7183	-18.7741	-13.2315
**54**	-13.1380	25.0088	-37.3259	-9.2597	-20.4772	-13.1380
**54**	-13.0218	11.7680	-46.9775	-9.7545	-29.4251	-13.0218
**55**	-12.5108	-38.6145	-34.1747	-10.2303	-48.8318	-12.5108
**55**	-12.0345	-30.7563	-40.6124	-10.7600	-34.9088	-12.0345
**55**	-11.5653	-39.5727	-56.9283	-13.5959	-49.2778	-11.5653
**55**	-11.5252	-35.8523	-40.4632	-10.6461	-44.6601	-11.5252
**55**	-11.2118	-9.8840	-40.5530	-10.7072	-34.5762	-11.2118
**55**	-11.2021	-16.6496	-29.5262	-10.5766	-27.9855	-11.2021
**55**	-11.1291	-20.9423	-46.9576	-11.2346	-28.8825	-11.1291
**55**	-10.8769	-25.1403	-46.7387	-12.1593	-42.4818	-10.8769
**55**	-10.5114	-35.6424	-44.2518	-12.7907	-44.8490	-10.5114
**55**	-10.4169	-21.7600	-35.5243	-11.7051	-12.1192	-10.4169
**56**	-14.1830	-8.8564	-31.8562	-5.7408	-20.9343	-14.1830
**56**	-13.7064	1.0132	-34.2297	-6.0833	-12.6849	-13.7064
**56**	-13.6645	-25.1134	-57.5025	-15.7181	-44.3644	-13.6645
**56**	-13.5320	-23.6290	-62.6683	-15.0014	-48.6397	-13.5320
**56**	-12.8553	12.3955	-51.4116	-10.1350	-16.4720	-12.8553
**56**	-12.7569	-12.3379	-44.3988	-7.9754	-40.5060	-12.7569
**56**	-12.7087	16.5573	-41.9856	-6.8657	-23.4981	-12.7087
**56**	-12.6397	-12.0491	-50.2470	-7.2176	-42.0270	-12.6397
**56**	-12.4116	-8.2579	-33.9495	-6.3058	-42.9889	-12.4116
**56**	-12.1113	-18.5716	-34.9932	-7.2034	-51.1063	-12.1113
**57**	-13.6550	-26.9699	-40.6004	-6.9055	-51.6924	-13.6550
**57**	-13.4206	-20.1427	-41.7333	-9.9497	-42.8487	-13.4206
**57**	-12.7985	0.4067	-31.3176	-7.6495	-21.1334	-12.7985

**57**	-12.6458	-7.7168	-39.1107	-7.9225	-36.7553	-12.6458
**57**	-12.5077	-4.9713	-40.4629	-7.3566	-23.8941	-12.5077
**57**	-12.1400	-22.4970	-35.3129	-7.2954	-51.5332	-12.1400
**57**	-12.1142	2.5505	-37.3789	-7.8197	-14.2406	-12.1142
**57**	-12.0795	-16.9935	-33.2184	-6.5594	-44.7513	-12.0795
**57**	-11.9635	-18.1802	-47.0230	-8.4971	-47.5477	-11.9635
**57**	-11.8314	-4.4014	-38.9400	-8.5301	-21.5823	-11.8314
**58**	-14.3016	-0.3993	-27.4885	-7.7850	-28.3905	-14.3016
**58**	-13.4936	-27.0620	-42.3317	-12.9667	-41.4724	-13.4936
**58**	-13.2147	-27.4859	-28.0230	-7.0678	-39.7205	-13.2147
**58**	-13.1740	-22.8668	-27.4706	-9.0573	-39.3521	-13.1740
**58**	-13.1523	-18.1139	-52.1759	-11.9675	-29.9761	-13.1523
**58**	-12.9894	156.3997	-25.8789	-8.0220	-24.5266	-12.9894
**58**	-12.7916	-11.4853	-30.7798	-10.9079	-30.0139	-12.7916
**58**	-12.6407	-25.9884	-40.7841	-11.3975	-39.0247	-12.6407
**58**	-12.1244	-13.3346	-34.9376	-9.6400	-27.7983	-12.1244
**58**	-12.0252	-14.2841	-36.6422	-9.9344	-40.2411	-12.0252
**59**	-13.2718	-20.2551	-39.2537	-9.4082	-40.0476	-13.2718
**59**	-13.0911	-28.4623	-40.8507	-9.1953	-50.1638	-13.0911
**59**	-12.2647	-27.3340	-31.1926	-8.5219	-46.9763	-12.2647
**59**	-11.9778	-17.2239	-32.3321	-8.0337	-24.7821	-11.9778
**59**	-11.9341	-10.3280	-40.7646	-10.9912	-27.2495	-11.9341
**59**	-11.9087	-16.1516	-32.4795	-13.6776	-21.4284	-11.9087
**59**	-11.7667	-11.6529	-32.7501	-8.8027	-39.3952	-11.7667
**59**	-11.6463	-0.6526	-34.8905	-10.6542	-34.6521	-11.6463
**59**	-11.4428	-20.8476	-49.0451	-12.3816	-48.8932	-11.4428
**59**	-11.3742	-20.4922	-45.3874	-11.5512	-50.4936	-11.3742
**60**	-13.2075	-48.4054	-40.1493	-10.8937	-49.1921	-13.2075
**60**	-12.7155	-35.5933	-34.3425	-10.8909	-34.8162	-12.7155
**60**	-12.6208	-43.9062	-35.6014	-8.3528	-43.7297	-12.6208
**60**	-12.4807	-39.5102	-33.1673	-8.2524	-26.5661	-12.4807
**60**	-12.3872	-49.0280	-55.8362	-11.7723	-52.6159	-12.3872
**60**	-12.0067	-44.7143	-44.5592	-11.8999	-47.4870	-12.0067
**60**	-11.9919	-61.8779	-43.8852	-8.1424	-56.4139	-11.9919
**60**	-11.9806	-34.5630	-36.6362	-9.5903	-38.8203	-11.9806
**60**	-11.9268	-38.1599	-35.1911	-9.4343	-26.4134	-11.9268
**60**	-11.4305	-53.1167	-31.8499	-8.6353	-53.4131	-11.4305
**61**	-12.5530	-49.8130	-51.9517	-9.8812	-51.0399	-12.5530
**61**	-12.2047	-61.3790	-46.0253	-6.9513	-53.7175	-12.2047
**61**	-12.0921	-54.1304	-31.6421	-6.4168	-38.5454	-12.0921
**61**	-11.9229	-51.1252	-29.0060	-7.6181	-37.7394	-11.9229
**61**	-11.9068	-26.2420	-29.1773	-6.7668	-34.8186	-11.9068
**61**	-11.7751	-51.9403	-36.6503	-7.0082	-42.8068	-11.7751
**61**	-11.6799	-28.9007	-38.1284	-6.3571	-31.8758	-11.6799
**61**	-11.5330	-56.7927	-38.2355	-8.3208	-47.8067	-11.5330
**61**	-11.3320	-33.9165	-33.1656	-10.0188	-28.0285	-11.3320

**61**	-10.9573	-24.1948	-33.2862	-7.3768	0.9153	-10.9573
**62**	-12.8540	-42.3809	-39.9832	-10.4330	-53.1147	-12.8540
**62**	-12.4902	-30.3087	-46.0509	-11.1391	-35.2665	-12.4902
**62**	-12.3279	-53.2756	-55.4473	-12.8699	-54.9192	-12.3279
**62**	-12.3272	-37.8065	-33.9747	-7.4054	-47.4412	-12.3272
**62**	-11.8563	-43.6080	-35.1313	-10.3697	-44.3985	-11.8563
**62**	-11.6217	-48.5963	-48.5129	-11.9105	-49.1575	-11.6217
**62**	-11.5146	-22.4542	-41.2770	-8.9547	-32.1601	-11.5146
**62**	-11.4508	-26.6763	-37.6921	-7.6513	-28.6058	-11.4508
**62**	-11.3525	-37.8352	-42.4645	-11.8782	-31.9592	-11.3525
**62**	-10.3463	-35.8754	-33.8431	-10.5629	-23.4638	-10.3463
**63**	-13.2525	-6.2756	-42.5933	-8.9734	-26.0298	-13.2525
**63**	-12.9550	-16.7276	-34.1030	-8.7086	-35.7748	-12.9550
**63**	-12.9088	29.5713	-39.5973	-8.1115	-16.8710	-12.9088
**63**	-12.7972	-21.3276	-39.8552	-9.5525	-35.7076	-12.7972
**63**	-12.5679	-24.0458	-42.6747	-9.4972	-49.8591	-12.5679
**63**	-12.5286	5.3555	-42.2068	-9.1755	-27.5184	-12.5286
**63**	-12.5125	-23.3497	-36.7474	-7.6593	-46.8865	-12.5125
**63**	-12.1636	-0.6526	-43.1836	-8.9224	-26.4689	-12.1636
**63**	-12.0483	-11.6229	-31.8981	-7.1367	-38.4928	-12.0483
**63**	-11.8618	-17.9441	-43.2079	-13.1748	-13.4143	-11.8618
**64**	-13.8097	-7.3049	-38.3513	-12.8912	-29.0650	-13.8097
**64**	-13.2814	-17.2722	-32.7194	-10.3465	-35.1301	-13.2814
**64**	-12.6986	-8.5669	-38.6921	-8.8118	-31.4763	-12.6986
**64**	-12.4663	0.8389	-43.2471	-11.4945	-14.0134	-12.4663
**64**	-12.4062	-23.5899	-39.3265	-10.2675	-46.5382	-12.4062
**64**	-12.3469	-23.5615	-42.8033	-9.0918	-46.6341	-12.3469
**64**	-12.1303	-11.5912	-46.0056	-8.6503	-38.6653	-12.1303
**64**	-12.0466	-8.8167	-38.4024	-8.9616	-17.4070	-12.0466
**64**	-11.5609	-16.4439	-42.0832	-10.2160	-40.0849	-11.5609
**64**	-11.5365	-22.3071	-41.7942	-8.5068	-43.2138	-11.5365
**65**	-12.9602	-21.1204	-37.6510	-12.2081	-34.8482	-12.9602
**65**	-12.3153	-20.7404	-41.5716	-11.1575	-36.4752	-12.3153
**65**	-11.9880	-8.1979	-31.4857	-9.0963	-35.2679	-11.9880
**65**	-11.9637	-26.5573	-43.3226	-9.6534	-45.3685	-11.9637
**65**	-11.8193	-10.5129	-40.4252	-11.6558	-31.7654	-11.8193
**65**	-11.7695	-26.2150	-36.8579	-9.8613	-47.6667	-11.7695
**65**	-11.6979	-26.2258	-23.6157	-8.2318	-47.6878	-11.6979
**65**	-11.6623	-19.9120	-35.4432	-8.4619	-39.8155	-11.6623
**65**	-11.4905	-19.9490	-41.6359	-9.0957	-43.1325	-11.4905
**65**	-11.2418	-21.1449	-46.6909	-11.4705	-43.5161	-11.2418
**66**	-13.6990	-26.1916	-45.1097	-8.6150	-43.8589	-13.6990
**66**	-13.3732	-20.1699	-42.8221	-12.1253	-31.0348	-13.3732
**66**	-13.2886	-17.6663	-49.9053	-12.7866	-40.6390	-13.2886
**66**	-13.0796	-9.1628	-37.1722	-9.0928	-25.3483	-13.0796
**66**	-13.0780	-19.4362	-38.7221	-9.4091	-11.8869	-13.0780

**66**	-12.8610	-23.8370	-49.1472	-11.9056	-36.0190	-12.8610
**66**	-12.8095	-19.1408	-47.6619	-10.9694	-42.8989	-12.8095
**66**	-12.1663	-25.0890	-30.4856	-8.1567	-30.2497	-12.1663
**66**	-12.1067	5.8700	-37.8185	-9.4969	-18.8537	-12.1067
**66**	-12.0772	-21.6829	-45.7581	-13.7578	-13.5120	-12.0772
**67**	-13.9796	-20.6041	-43.0267	-10.7297	-34.4042	-13.9796
**67**	-13.4776	-25.7803	-39.5777	-10.3523	-40.7110	-13.4776
**67**	-13.0869	-20.8451	-34.4727	-9.0522	-34.8810	-13.0869
**67**	-12.7911	-24.7405	-39.2548	-8.7914	-47.0249	-12.7911
**67**	-12.5278	-20.3770	-39.3209	-10.2211	-43.9894	-12.5278
**67**	-12.5162	-11.4448	-42.1218	-8.9182	-16.9667	-12.5162
**67**	-12.3918	-25.9171	-49.7430	-10.3040	-46.3417	-12.3918
**67**	-12.1342	-18.9841	-44.7963	-8.9992	-40.4572	-12.1342
**67**	-12.0536	-16.1618	-40.0572	-9.5105	-37.3238	-12.0536
**67**	-11.9251	14.2625	-37.3588	-9.3029	-24.1452	-11.9251
**68**	-14.0554	-18.1182	-37.9432	-11.7738	-33.8052	-14.0554
**68**	-13.9820	-17.5183	-50.4422	-12.3431	-23.0904	-13.9820
**68**	-13.6401	-17.5452	-46.8268	-11.9360	-26.7259	-13.6401
**68**	-13.4916	-14.3456	-37.4747	-10.7835	-28.3440	-13.4916
**68**	-13.1126	-18.3998	-37.6185	-13.0567	-34.7212	-13.1126
**68**	-13.0386	-24.3894	-39.0903	-12.1890	-18.4818	-13.0386
**68**	-12.9899	-25.7458	-39.8891	-11.8399	-30.8272	-12.9899
**68**	-12.9545	-16.1429	-32.9921	-10.8480	-27.9704	-12.9545
**68**	-12.7784	-25.3711	-45.4381	-13.5095	-32.5497	-12.7784
**68**	-12.6078	-25.7272	-50.4269	-12.3277	-37.7286	-12.6078
**69**	-12.9563	-23.8870	-40.8116	-11.9337	-36.5296	-12.9563
**69**	-12.3779	-22.3282	-39.4844	-11.8521	-43.2589	-12.3779
**69**	-11.8656	-25.0779	-39.2201	-10.2093	-37.8377	-11.8656
**69**	-11.7229	-28.6378	-42.2671	-11.7101	-48.2044	-11.7229
**69**	-11.5304	-16.2834	-31.1486	-9.6968	-18.6652	-11.5304
**69**	-10.8914	-18.4420	-36.4562	-9.0827	-38.3225	-10.8914
**69**	-10.8306	-21.4203	-40.4016	-9.4648	-36.4363	-10.8306
**69**	-10.5235	37.0160	-30.8162	-9.0301	13.1881	-10.5235
**69**	-10.4766	-25.2370	-45.2662	-9.5556	-39.9727	-10.4766
**69**	-9.7537	1.3724	-35.7753	-9.8240	-7.9178	-9.7537
**70**	-13.5819	10.0278	-42.4961	-10.1223	-40.8663	-13.5819
**70**	-13.3155	0.5682	-39.9373	-9.6047	-33.6458	-13.3155
**70**	-13.1174	5.3806	-48.4417	-9.3742	-41.6500	-13.1174
**70**	-13.0864	5.5582	-29.0141	-8.5717	-35.6014	-13.0864
**70**	-12.9534	0.9420	-32.6021	-8.0287	-46.4601	-12.9534
**70**	-12.8138	4.2039	-42.0227	-9.0392	-42.7179	-12.8138
**70**	-12.6226	-0.3812	-41.6789	-8.1908	-44.8631	-12.6226
**70**	-12.5749	-1.1950	-42.8061	-8.9154	-52.2435	-12.5749
**70**	-12.5177	21.5870	-42.2501	-10.9006	-17.8454	-12.5177
**70**	-12.1809	3.3880	-42.1251	-12.0814	-12.7195	-12.1809
**71**	-13.1870	2.8624	-27.5499	-8.8430	-34.3427	-13.1870

**71**	-12.9428	3.1183	-43.6675	-13.1180	-41.9389	-12.9428
**71**	-12.5911	5.8723	-46.0598	-10.6032	-30.0194	-12.5911
**71**	-12.4529	-1.7383	-41.6535	-9.2859	-45.1535	-12.4529
**71**	-12.2625	-0.5289	-42.1070	-10.0975	-40.7761	-12.2625
**71**	-12.0157	21.8958	-34.8732	-9.2158	-44.8307	-12.0157
**71**	-12.0082	12.1940	-40.1841	-10.0431	-37.3950	-12.0082
**71**	-11.9563	0.0098	-39.1102	-9.5417	-42.3112	-11.9563
**71**	-11.8663	22.5822	-41.6244	-9.1570	-14.8974	-11.8663
**71**	-11.8458	21.3186	-37.8986	-11.2390	-24.7929	-11.8458
**72**	-13.0397	9.2150	-40.0832	-9.2682	-27.8820	-13.0397
**72**	-12.8774	0.7065	-45.2566	-11.2030	-40.8816	-12.8774
**72**	-12.8314	5.6678	-33.5344	-9.5180	-22.8113	-12.8314
**72**	-12.5340	-0.3338	-30.7183	-10.6726	-21.1406	-12.5340
**72**	-12.3659	-0.0542	-38.7093	-12.1875	-19.0222	-12.3659
**72**	-12.3029	2.3869	-37.9407	-11.7040	-17.4233	-12.3029
**72**	-12.1687	7.7400	-48.0398	-12.5223	-24.5363	-12.1687
**72**	-12.0770	-0.9942	-33.5715	-9.2360	-42.9842	-12.0770
**72**	-11.9319	-0.5503	-42.4828	-9.8406	-37.1244	-11.9319
**72**	-11.7902	6.7056	-40.9639	-10.5717	-22.2223	-11.7902
**73**	-12.1242	11.0783	-44.0825	-10.9884	-30.8833	-12.1242
**73**	-12.0210	-4.3143	-42.0468	-9.7059	-49.1064	-12.0210
**73**	-11.9897	1.5939	-47.1845	-11.5558	-35.3343	-11.9897
**73**	-11.8780	0.4270	-40.2111	-9.5763	-34.9995	-11.8780
**73**	-11.7647	-1.3834	-36.7558	-11.7671	-51.0333	-11.7647
**73**	-11.6986	-5.0755	-45.2331	-8.8916	-48.0791	-11.6986
**73**	-11.3383	11.7065	-45.1391	-11.6144	-34.6996	-11.3383
**73**	-11.1974	44.0495	-36.1861	-10.3426	5.6609	-11.1974
**73**	-10.9940	2.4898	-40.9806	-12.2828	-37.2986	-10.9940
**73**	-10.9881	6.1537	-35.0667	-8.8060	-40.7933	-10.9881
**74**	-13.8489	-32.5307	-48.8934	-10.0055	-46.4677	-13.8489
**74**	-13.7663	0.4675	-43.6974	-12.7331	-28.3803	-13.7663
**74**	-13.6627	-38.9106	-47.2444	-8.2327	-52.8485	-13.6627
**74**	-13.5468	-40.5021	-59.6758	-11.9006	-52.4207	-13.5468
**74**	-13.3689	-41.0621	-47.6604	-9.5703	-49.9103	-13.3689
**74**	-12.8775	8.2213	-43.6956	-10.7965	-23.6472	-12.8775
**74**	-12.7754	-27.1977	-36.3298	-7.7630	-44.8754	-12.7754
**74**	-12.7703	-17.1736	-32.1398	-7.9922	-2.1970	-12.7703
**74**	-12.7087	-20.3114	-33.7432	-7.3479	-40.3846	-12.7087
**74**	-12.5402	-19.8285	-32.1602	-10.4698	-38.4415	-12.5402
**75**	-14.0655	-33.8764	-60.1582	-12.0739	-44.1596	-14.0655
**75**	-13.6964	-38.0821	-43.9529	-8.0344	-52.5235	-13.6964
**75**	-13.5125	-37.6699	-42.7299	-8.4829	-50.9122	-13.5125
**75**	-13.4173	-34.2776	-37.9330	-7.9206	-42.9651	-13.4173
**75**	-13.4130	-17.6552	-43.2731	-15.6438	-22.6807	-13.4130
**75**	-13.3757	-31.0101	-45.5880	-10.3648	-46.5985	-13.3757
**75**	-13.1609	-32.9860	-45.7972	-8.8278	-45.5647	-13.1609

**75**	-12.9492	-12.1716	-44.4738	-9.2814	-27.7082	-12.9492
**75**	-12.8156	-0.4991	-37.4895	-8.7986	-26.4949	-12.8156
**75**	-12.7967	-26.6143	-52.9189	-12.3132	-25.1914	-12.7967
**76**	-13.9292	-39.2634	-46.8367	-7.1933	-44.9765	-13.9292
**76**	-13.6377	-40.0015	-44.2593	-7.1998	-44.0657	-13.6377
**76**	-13.2951	-39.3029	-43.2114	-8.6893	-48.7937	-13.2951
**76**	-13.1568	-5.9274	-37.0760	-8.4328	-27.8270	-13.1568
**76**	-13.0170	-30.1591	-36.7141	-9.7943	-31.9130	-13.0170
**76**	-12.9779	-36.4143	-49.2342	-12.0558	-42.7715	-12.9779
**76**	-12.9476	-40.7417	-41.7939	-9.1464	-42.2070	-12.9476
**76**	-12.7055	-20.2260	-30.4988	-9.6003	-29.8102	-12.7055
**76**	-12.7024	-24.1763	-31.6757	-9.2527	-28.8197	-12.7024
**76**	-12.6973	-22.3260	-40.1837	-11.7373	-24.8877	-12.6973
**77**	-13.1494	-40.9720	-44.4650	-7.7308	-53.6862	-13.1494
**77**	-12.5211	-19.7384	-27.6489	-7.9245	-18.9267	-12.5211
**77**	-12.2440	-24.5346	-37.6091	-8.0572	-22.6872	-12.2440
**77**	-12.0505	-23.4653	-45.2955	-11.8167	-29.4346	-12.0505
**77**	-12.0385	-37.3485	-34.3170	-9.0752	-56.5688	-12.0385
**77**	-11.9113	-23.5212	-35.7438	-10.3223	-43.5955	-11.9113
**77**	-11.8806	-29.2447	-28.5172	-7.3497	-23.6369	-11.8806
**77**	-11.8451	-10.1206	-35.8086	-7.5769	-30.9538	-11.8451
**77**	-11.7148	-31.6879	-39.8384	-10.2185	-28.9841	-11.7148
**77**	-11.6427	-14.1719	-33.7296	-7.2354	-36.9870	-11.6427
**78**	-13.7127	-11.9609	-38.9126	-9.2917	-47.6524	-13.7127
**78**	-13.7080	-15.6706	-51.4639	-8.9759	-39.7048	-13.7080
**78**	-12.6542	-17.4139	-48.4204	-11.3897	-46.6990	-12.6542
**78**	-12.5349	0.2403	-41.5929	-11.5606	-21.2611	-12.5349
**78**	-12.3488	18.2204	-40.8288	-10.3538	-10.6153	-12.3488
**78**	-12.3158	24.4893	-35.8284	-9.3144	-13.5906	-12.3158
**78**	-12.2359	-10.4053	-47.4087	-12.8263	-40.3314	-12.2359
**78**	-12.0949	-8.6991	-44.0252	-10.8340	-39.1762	-12.0949
**78**	-12.0448	-11.0023	-56.2595	-14.1227	-38.8541	-12.0448
**78**	-11.9904	-14.2157	-50.3168	-12.2318	-35.4613	-11.9904
**79**	-13.5783	-9.6244	-28.5713	-8.1905	-32.1612	-13.5783
**79**	-13.5338	1.6005	-32.8018	-7.9744	-28.6620	-13.5338
**79**	-13.3020	-15.3187	-39.8662	-8.5615	-44.3143	-13.3020
**79**	-13.0831	-9.9464	-50.4527	-13.0995	-15.7197	-13.0831
**79**	-13.0581	-8.9146	-32.2325	-8.1431	-41.1142	-13.0581
**79**	-13.0538	-13.4696	-42.1817	-11.2506	-34.2131	-13.0538
**79**	-12.7238	-12.9127	-38.9388	-8.4341	-43.9016	-12.7238
**79**	-12.6669	7.3694	-33.2255	-7.9134	-25.0437	-12.6669
**79**	-12.3578	-10.0294	-39.3106	-8.8944	-40.4550	-12.3578
**79**	-11.8933	7.1920	-37.1769	-12.7055	-29.6640	-11.8933
**80**	-13.5807	-2.9438	-25.1830	-7.7461	-35.7039	-13.5807
**80**	-12.8395	-8.2114	-37.1248	-11.3045	-42.8457	-12.8395
**80**	-12.6798	-16.3280	-38.2686	-10.4726	-41.1080	-12.6798

**80**	-12.3440	-6.4297	-38.7004	-10.3908	-18.9840	-12.3440
**80**	-12.2792	-16.9464	-46.8010	-10.1235	-37.4462	-12.2792
**80**	-12.1758	-6.1300	-38.3413	-7.8417	-16.8723	-12.1758
**80**	-12.1220	54.1123	-37.2876	-7.8236	3.5829	-12.1220
**80**	-12.1200	0.6304	-42.1236	-8.7064	-26.9395	-12.1200
**80**	-12.0274	-8.2192	-32.0882	-9.6428	-37.5808	-12.0274
**80**	-12.0254	-12.6888	-48.2743	-12.7981	-37.0943	-12.0254
**81**	-13.8635	-0.9588	-25.7723	-8.4823	-21.8148	-13.8635
**81**	-13.3440	-19.4423	-38.7022	-8.3717	-51.1949	-13.3440
**81**	-13.2104	-9.4154	-36.7844	-9.8440	-31.7950	-13.2104
**81**	-12.9909	-14.5574	-36.3770	-7.3751	-33.1154	-12.9909
**81**	-12.9057	-20.0879	-46.3741	-12.7324	-50.0250	-12.9057
**81**	-12.6703	5.7517	-37.6269	-9.9348	-40.4023	-12.6703
**81**	-12.4983	-8.4826	-28.3721	-6.8995	-13.9851	-12.4983
**81**	-12.3620	-13.3281	-44.1061	-10.5991	-37.8319	-12.3620
**81**	-12.2562	13.0142	-36.2380	-7.2822	1.1568	-12.2562
**81**	-12.1909	1.5850	-48.5786	-9.2893	-19.2153	-12.1909
**82**	-13.3763	-11.2995	-27.8871	-7.0627	-31.7551	-13.3763
**82**	-12.9238	-11.9657	-30.7983	-6.4638	-41.2247	-12.9238
**82**	-12.7450	-11.6619	-27.9851	-7.9038	-43.7151	-12.7450
**82**	-12.6822	-19.3048	-31.9379	-8.8644	-47.4579	-12.6822
**82**	-12.0889	-13.4276	-38.0894	-8.9778	-41.3184	-12.0889
**82**	-12.0323	89.3799	-30.4810	-7.1199	27.9690	-12.0323
**82**	-11.6772	-4.4790	-32.3876	-9.2907	-42.1362	-11.6772
**82**	-11.6658	-9.6134	-33.7373	-7.7566	-39.9283	-11.6658
**82**	-11.5773	8.0243	-44.5695	-7.9707	-12.5444	-11.5773
**82**	-11.2818	-21.0810	-27.9960	-7.6907	-40.3111	-11.2818
**83**	-13.5041	1.8909	-29.2747	-8.8026	-10.2890	-13.5041
**83**	-13.3887	-5.3410	-48.4571	-9.9986	-28.2679	-13.3887
**83**	-13.2746	-2.6635	-42.9937	-10.2999	-31.7051	-13.2746
**83**	-13.2689	-3.2716	-41.2662	-12.3487	-28.8410	-13.2689
**83**	-13.1639	-15.4407	-27.1616	-11.2763	-36.2101	-13.1639
**83**	-12.9995	-12.4921	-36.6920	-9.4923	-29.4996	-12.9995
**83**	-12.5798	-20.9689	-40.9530	-9.9993	-23.7916	-12.5798
**83**	-12.3839	-18.7045	-49.1671	-13.8296	-39.0576	-12.3839
**83**	-12.3249	-5.0651	-41.6906	-12.0144	-26.8852	-12.3249
**83**	-12.1410	-9.6615	-37.0586	-10.4422	-19.9166	-12.1410
**84**	-13.7292	-43.5559	-36.4758	-10.7279	-35.9567	-13.7292
**84**	-12.4794	-42.7991	-50.0231	-12.2554	-44.2433	-12.4794
**84**	-12.2209	-46.8118	-42.0033	-9.0031	-43.5279	-12.2209
**84**	-11.9696	-40.1553	-38.3872	-9.2466	-42.2933	-11.9696
**84**	-11.9297	-49.3002	-48.8397	-8.9035	-48.0797	-11.9297
**84**	-11.8177	-42.7888	-46.1511	-11.0035	-28.1249	-11.8177
**84**	-11.7754	-35.2531	-26.8742	-10.6422	-18.3834	-11.7754
**84**	-11.6475	-36.0332	-37.4634	-8.5906	-40.7431	-11.6475
**84**	-11.3862	-30.1285	-35.6603	-9.5415	-24.7191	-11.3862

**84**	-11.3612	-25.1234	-44.7449	-10.8499	-25.9267	-11.3612
**85**	-13.9937	-37.1017	-38.9227	-9.2007	-26.8317	-13.9937
**85**	-13.6675	-47.0856	-28.4074	-9.7649	-19.4134	-13.6675
**85**	-12.8726	-39.8817	-33.2359	-9.2673	-12.6688	-12.8726
**85**	-12.6802	-48.6437	-39.3814	-11.9011	-21.6797	-12.6802
**85**	-12.1771	-24.3002	-31.4457	-10.0486	-22.8425	-12.1771
**85**	-12.0712	-48.3243	-33.3138	-7.8322	-21.6805	-12.0712
**85**	-12.0576	-49.5344	-44.1639	-11.8245	-39.1487	-12.0576
**85**	-12.0073	-42.6267	-32.3827	-9.2204	-27.7584	-12.0073
**85**	-11.8975	-45.9039	-31.2898	-8.6356	-30.4558	-11.8975
**85**	-11.8289	-37.0484	-34.4369	-10.3849	-38.0292	-11.8289
**86**	-13.4674	12.9371	-39.9251	-9.9919	-20.1446	-13.4674
**86**	-13.0039	-6.6287	-43.8492	-11.0712	-34.8960	-13.0039
**86**	-12.8396	-6.2100	-51.1604	-10.2534	-41.6212	-12.8396
**86**	-12.6036	0.6042	-45.1440	-13.0242	-8.8051	-12.6036
**86**	-12.5198	7.2407	-43.5730	-11.3119	-34.5468	-12.5198
**86**	-12.4805	11.1198	-35.9104	-11.1566	-20.5348	-12.4805
**86**	-12.4163	10.2412	-43.1777	-9.5461	-10.6918	-12.4163
**86**	-12.3050	6.9629	-33.0746	-9.6911	-36.3442	-12.3050
**86**	-12.1339	-0.3357	-41.5631	-8.6176	-4.2349	-12.1339
**86**	-12.1212	23.1674	-46.3229	-9.8474	-10.7022	-12.1212
**87**	-12.9954	-4.0605	-49.2907	-11.5911	-21.4262	-12.9954
**87**	-12.9733	-2.2964	-40.8098	-10.1865	-16.1659	-12.9733
**87**	-12.7482	-8.7386	-51.3103	-12.5613	-34.5428	-12.7482
**87**	-12.7213	-11.8251	-48.2523	-11.3425	-31.6242	-12.7213
**87**	-12.6088	-5.0393	-37.0931	-9.6164	-27.2284	-12.6088
**87**	-12.5390	7.0597	-36.7786	-8.5678	-12.8320	-12.5390
**87**	-12.5003	21.2080	-39.4921	-9.5336	-27.3395	-12.5003
**87**	-12.2936	-1.8823	-43.7573	-8.8381	-23.6493	-12.2936
**87**	-12.2008	-8.4143	-42.1121	-11.8940	-35.4405	-12.2008
**87**	-12.1806	-3.5948	-39.3524	-10.5868	-22.9031	-12.1806
**88**	-12.7342	5.8495	-40.1297	-9.6141	-22.5045	-12.7342
**88**	-12.5622	68.0577	-36.1308	-8.6487	-31.1004	-12.5622
**88**	-12.1604	-14.7695	-35.9630	-10.3657	-43.6620	-12.1604
**88**	-12.0852	7.8119	-36.9324	-9.7443	-39.6953	-12.0852
**88**	-11.9696	-8.6191	-28.4365	-10.5247	-37.8635	-11.9696
**88**	-11.9201	-7.7737	-45.7793	-9.2854	-37.5770	-11.9201
**88**	-11.5748	19.8798	-43.5620	-8.9382	-30.0991	-11.5748
**88**	-11.1686	-14.1114	-37.9269	-8.6622	-42.6069	-11.1686
**88**	-11.1375	15.9338	-31.9512	-11.0535	-6.9461	-11.1375
**88**	-10.1629	33.4688	-31.2268	-8.6143	0.9080	-10.1629
**89**	-11.2663	-19.5650	-25.4088	-11.1716	-31.0858	-11.2663
**89**	-11.2613	-23.5647	-42.9328	-12.8033	-43.1894	-11.2613
**89**	-11.2119	-9.4170	-48.1790	-12.1475	-27.8597	-11.2119
**89**	-10.7179	-7.5765	-36.9239	-13.3674	-41.9690	-10.7179
**89**	-10.6897	-22.5527	-30.8005	-12.2071	-40.8529	-10.6897

**89**	-10.6641	-16.2914	-42.0831	-13.1148	-45.9563	-10.6641
**89**	-10.5300	6.7861	-37.9527	-11.8838	-12.9789	-10.5300
**89**	-10.2582	0.4689	-39.4858	-11.5951	-16.6348	-10.2582
**89**	-10.0778	-11.2147	-44.0639	-14.5549	-34.7081	-10.0778
**89**	-9.9028	-2.4405	-35.4604	-12.0495	-0.3214	-9.9028
**90**	-13.8590	13.1680	-38.0706	-10.2550	-27.9050	-13.8590
**90**	-13.1791	8.9931	-37.2911	-14.0228	-41.0536	-13.1791
**90**	-13.1683	80.5309	-49.5756	-13.9895	-36.4972	-13.1683
**90**	-13.1632	-0.5836	-39.6205	-9.5786	-49.5375	-13.1632
**90**	-13.1429	8.5313	-37.6738	-10.2067	-33.4056	-13.1429
**90**	-12.8594	47.4779	-38.4251	-12.0024	7.5140	-12.8594
**90**	-12.6800	3.2718	-34.7189	-9.7617	-40.6281	-12.6800
**90**	-12.6717	1.0736	-64.0310	-16.4241	-44.2712	-12.6717
**90**	-12.5353	6.0032	-44.2606	-13.0404	-49.4581	-12.5353
**90**	-12.3582	1.1166	-49.6893	-12.3235	-49.7518	-12.3582
**91**	-13.3454	9.9835	-30.9117	-10.1492	-29.3657	-13.3454
**91**	-13.1630	6.7998	-48.2559	-14.4090	-33.1569	-13.1630
**91**	-12.9596	1.3747	-27.2621	-11.7099	-42.8474	-12.9596
**91**	-12.7923	14.5641	-44.7034	-12.1732	-32.7024	-12.7923
**91**	-12.7810	0.1678	-43.2299	-11.6169	-46.2483	-12.7810
**91**	-12.5531	1.3355	-37.7158	-10.4195	-44.0535	-12.5531
**91**	-12.4248	8.7583	-35.7176	-10.8665	-31.8071	-12.4248
**91**	-12.3177	6.0665	-28.2569	-11.9020	-39.8519	-12.3177
**91**	-11.9176	13.7163	-39.0770	-13.0110	-39.9833	-11.9176
**91**	-11.7936	19.8411	-21.1634	-11.9447	-14.2891	-11.7936
**92**	-13.0542	13.4183	-36.3850	-12.4458	-17.5246	-13.0542
**92**	-13.0233	12.2847	-37.9384	-12.1429	-26.6823	-13.0233
**92**	-12.6971	20.8813	-34.0421	-11.0487	-28.3829	-12.6971
**92**	-12.5321	0.4878	-42.7410	-12.7845	-36.2890	-12.5321
**92**	-12.3533	4.8483	-37.0533	-11.5794	-33.6788	-12.3533
**92**	-12.3520	3.3256	-31.9319	-11.0824	-39.4432	-12.3520
**92**	-12.2147	2.7955	-50.3960	-13.1704	-34.1562	-12.2147
**92**	-12.1991	9.8715	-37.9514	-14.9525	-15.9127	-12.1991
**92**	-12.1954	21.5887	-34.3911	-12.3635	-22.7578	-12.1954
**92**	-12.1244	12.8165	-30.1254	-15.3613	-36.1771	-12.1244
**93**	-13.2111	4.8886	-43.3855	-11.2262	-20.5617	-13.2111
**93**	-13.1524	-8.2416	-41.0684	-14.4546	-23.9217	-13.1524
**93**	-12.8903	-15.9776	-42.4820	-8.4368	-40.4906	-12.8903
**93**	-12.8487	-11.1363	-26.3779	-9.3881	-31.9574	-12.8487
**93**	-12.8018	-7.3385	-57.2448	-15.1656	-36.3498	-12.8018
**93**	-12.6979	-16.9454	-29.0977	-8.3558	-49.7025	-12.6979
**93**	-12.6234	11.1479	-34.8955	-8.3154	-27.8868	-12.6234
**93**	-12.4812	-0.4833	-46.2384	-11.1240	-33.2299	-12.4812
**93**	-12.4605	2.1008	-35.7131	-8.9620	-21.5447	-12.4605
**93**	-12.4348	-14.7429	-37.8944	-11.4776	-42.6508	-12.4348
**94**	-14.7354	-1.0279	-32.3593	-14.5450	-33.8984	-14.7354

**94**	-14.0562	-5.2422	-42.2528	-12.7699	-38.1726	-14.0562
**94**	-13.2998	6.8338	-43.0476	-11.0767	-30.9423	-13.2998
**94**	-13.2900	50.3850	-25.6118	-10.4848	-5.6198	-13.2900
**94**	-13.2433	-1.2567	-41.7737	-14.3898	-39.5145	-13.2433
**94**	-13.1513	16.5591	-40.5401	-9.5555	-4.3337	-13.1513
**94**	-12.9513	10.0144	-29.1608	-9.4250	-33.8971	-12.9513
**94**	-12.7658	-0.9663	-37.9641	-10.2871	-44.1351	-12.7658
**94**	-12.1620	0.4050	-25.9777	-8.6356	-18.4207	-12.1620
**94**	-12.0182	-2.4422	-45.8407	-11.0095	-29.6805	-12.0182
**95**	-12.3766	-12.1972	-51.2275	-17.6383	-34.0699	-12.3766
**95**	-11.8799	-9.9223	-27.5891	-7.7444	-49.4120	-11.8799
**95**	-11.8656	-14.4250	-35.3432	-9.1372	-33.3051	-11.8656
**95**	-11.8068	-10.0160	-40.7283	-10.3029	-39.7951	-11.8068
**95**	-11.6522	-9.1207	-52.8089	-12.1215	-30.0574	-11.6522
**95**	-11.5235	-15.4745	-38.7375	-10.3189	-47.0297	-11.5235
**95**	-11.4101	-11.8624	-41.8945	-9.0792	-37.4561	-11.4101
**95**	-11.1561	-5.8247	-34.9782	-9.8621	-23.7122	-11.1561
**95**	-11.1332	2.0815	-34.5314	-8.7344	-16.2902	-11.1332
**95**	-10.9658	-12.6013	-23.0802	-7.6482	-30.4575	-10.9658
**96**	-14.6195	-0.8405	-37.3090	-6.3603	-22.8470	-14.6195
**96**	-14.0935	4.1846	-41.0414	-8.6213	-34.7579	-14.0935
**96**	-13.3558	4.4736	-37.5415	-9.2392	-46.7056	-13.3558
**96**	-13.2010	2.0624	-33.4244	-6.5634	-40.1751	-13.2010
**96**	-13.0038	1.9770	-39.0518	-7.6848	-31.0678	-13.0038
**96**	-12.9538	15.9759	-42.9145	-9.0495	-20.3619	-12.9538
**96**	-12.9372	10.8657	-40.3189	-9.7336	-16.1621	-12.9372
**96**	-12.8614	-7.4547	-41.0380	-10.7541	-33.5752	-12.8614
**96**	-12.8274	23.7866	-48.8104	-11.1061	0.7036	-12.8274
**96**	-12.6846	84.7454	-44.0955	-10.3136	-23.7683	-12.6846
**97**	-13.3304	-24.5703	-49.0163	-11.0230	-30.8610	-13.3304
**97**	-13.1635	-20.0867	-37.7952	-9.8732	-28.3674	-13.1635
**97**	-12.8602	-25.4620	-38.7572	-9.8146	-39.0184	-12.8602
**97**	-12.7921	-17.9071	-41.4749	-12.1254	-31.8300	-12.7921
**97**	-12.7398	-25.4656	-43.9066	-12.2088	-29.7967	-12.7398
**97**	-12.6935	-21.8543	-40.8358	-10.1121	-39.5953	-12.6935
**97**	-12.4111	-22.7470	-45.4096	-11.0269	-6.1895	-12.4111
**97**	-12.3330	-27.4712	-47.0457	-9.9912	-46.6368	-12.3330
**97**	-12.2227	-18.9319	-49.1407	-13.3323	-34.1142	-12.2227
**97**	-12.1864	-16.1442	-41.2943	-9.1904	-34.5707	-12.1864
**98**	-13.4227	-17.5114	-40.4191	-9.3156	-36.4300	-13.4227
**98**	-13.2230	-19.9317	-33.6085	-9.8399	-35.2011	-13.2230
**98**	-12.8312	-20.1104	-46.0131	-11.0959	-40.9733	-12.8312
**98**	-12.5762	-14.6197	-46.8107	-11.8351	-33.8089	-12.5762
**98**	-12.5660	-7.9760	-56.1773	-13.5254	-37.1161	-12.5660
**98**	-12.5300	-24.2390	-37.9830	-12.2539	-48.1097	-12.5300
**98**	-11.9017	-21.3340	-32.4216	-9.5874	-46.0007	-11.9017

**98**	-10.9865	5.9273	-42.1137	-9.9681	-13.9880	-10.9865
**98**	-10.8688	-16.8738	-34.3212	-9.9346	-38.2974	-10.8688
**98**	-10.7222	-19.2029	-40.2702	-10.3189	-39.6409	-10.7222
**99**	-14.1808	-15.6865	-51.9739	-14.5404	-37.5294	-14.1808
**99**	-13.3362	-11.9272	-48.8634	-14.3463	-1.9061	-13.3362
**99**	-13.2625	-13.6035	-37.0675	-11.5392	-38.7433	-13.2625
**99**	-12.5848	4.9486	-41.5335	-12.8515	0.8082	-12.5848
**99**	-12.4844	-16.9982	-41.8407	-11.9458	-19.3008	-12.4844
**99**	-12.2211	-12.8482	-31.5898	-9.8695	-28.4952	-12.2211
**99**	-12.0000	-11.6801	-42.8295	-9.9208	-27.0926	-12.0000
**99**	-11.7579	-15.8025	-28.3239	-8.6243	-6.8642	-11.7579
**99**	-11.6953	-20.3853	-45.8889	-8.6606	-27.7201	-11.6953
**99**	-11.4940	-9.8924	-37.9150	-9.3780	-9.9677	-11.4940
**100**	-13.7395	-23.0151	-34.7119	-12.0881	-22.1072	-13.7395
**100**	-13.1641	-28.4244	-39.2442	-10.2343	-21.8913	-13.1641
**100**	-13.1256	-39.4389	-34.6059	-9.8571	-34.8704	-13.1256
**100**	-12.5709	-36.8977	-48.2958	-13.7595	-38.6504	-12.5709
**100**	-12.5023	-46.5177	-44.8318	-10.6950	-40.1470	-12.5023
**100**	-12.2203	-52.8602	-31.1938	-9.9321	-47.2122	-12.2203
**100**	-12.0710	-41.2436	-34.0261	-9.5133	-35.8714	-12.0710
**100**	-12.0382	-43.7804	-35.6730	-11.4595	-13.6700	-12.0382
**100**	-11.9937	-39.9087	-32.4278	-12.3799	-39.4459	-11.9937
**100**	-11.2824	-39.9517	-40.4745	-9.6173	-11.6118	-11.2824
**101**	-13.3825	-37.2189	-31.0806	-14.5262	-38.6110	-13.3825
**101**	-13.2907	-38.7707	-29.4022	-9.5721	-38.1629	-13.2907
**101**	-13.1736	-34.6124	-40.0989	-11.9855	-37.9284	-13.1736
**101**	-12.9531	-30.0726	-37.8847	-8.5912	-34.8291	-12.9531
**101**	-12.3782	-35.1537	-44.1844	-11.3348	-38.6330	-12.3782
**101**	-12.3331	-39.9221	-46.1734	-9.4938	-47.9343	-12.3331
**101**	-11.9863	-5.9200	-32.2947	-10.3757	-21.5055	-11.9863
**101**	-11.9631	-15.9066	-38.6443	-11.6264	-38.1876	-11.9631
**101**	-11.8252	-35.3674	-30.3239	-9.3134	-28.7323	-11.8252
**101**	-11.7870	-32.8359	-42.1618	-10.4219	-44.3118	-11.7870
**102**	-13.9996	-36.7640	-30.5189	-8.8516	-40.3827	-13.9996
**102**	-13.0901	-28.8061	-41.7748	-9.6394	-42.1042	-13.0901
**102**	-13.0091	-34.6618	-31.2806	-12.9475	-40.4160	-13.0091
**102**	-12.8506	-29.6797	-32.4309	-10.1556	-42.0117	-12.8506
**102**	-12.3459	-37.1109	-47.9727	-14.3032	-20.5489	-12.3459
**102**	-12.1038	-27.7561	-32.8162	-8.8914	-14.9359	-12.1038
**102**	-12.0589	-25.3789	-37.0422	-9.9461	-24.7876	-12.0589
**102**	-12.0402	-21.8883	-40.9404	-9.3810	-14.4076	-12.0402
**102**	-12.0375	-35.2435	-37.3068	-9.0291	-33.1743	-12.0375
**102**	-11.6383	-39.9270	-46.4264	-10.6671	-20.1731	-11.6383
**103**	-13.9227	61.9473	-36.7259	-14.0740	-34.3617	-13.9227
**103**	-13.4717	71.4960	-41.5629	-10.5234	-33.6952	-13.4717
**103**	-13.1443	84.3240	-36.3993	-11.4807	-41.3370	-13.1443

**103**	-13.1089	69.3533	-41.2107	-10.1049	-29.3865	-13.1089
**103**	-13.0656	94.8442	-39.1437	-10.5228	4.4025	-13.0656
**103**	-13.0345	68.6549	-47.7202	-11.6415	-25.8877	-13.0345
**103**	-13.0237	70.8886	-50.9413	-15.2267	-15.9869	-13.0237
**103**	-13.0026	65.7438	-53.9544	-13.7864	-15.9103	-13.0026
**103**	-12.8744	59.6733	-24.4681	-10.1935	-49.8896	-12.8744
**103**	-12.6055	71.7170	-44.2729	-9.9470	-6.9944	-12.6055
**104**	-13.2724	19.5220	-39.9818	-6.5987	-49.4039	-13.2724
**104**	-13.1122	21.5552	-39.1112	-5.6352	-28.1167	-13.1122
**104**	-13.1041	22.8839	-37.1550	-9.4690	-29.9720	-13.1041
**104**	-13.0803	33.8209	-38.4192	-8.9328	-12.2988	-13.0803
**104**	-12.9031	20.4442	-46.5151	-13.8524	-25.7183	-12.9031
**104**	-12.8882	31.8225	-31.9359	-5.8592	-7.2517	-12.8882
**104**	-12.4466	19.6747	-38.3936	-8.4242	-25.6282	-12.4466
**104**	-12.1980	32.7334	-30.4606	-5.9682	-22.8145	-12.1980
**104**	-12.1112	25.1185	-31.6749	-6.2887	-28.6773	-12.1112
**104**	-11.8779	28.2488	-33.3980	-6.0777	-21.9809	-11.8779
**105**	-13.2919	-26.3136	-29.3754	-10.0072	-34.1212	-13.2919
**105**	-13.1857	-24.1791	-31.7074	-8.1089	-40.4823	-13.1857
**105**	-12.7027	-18.4323	-41.9287	-11.5208	-36.6389	-12.7027
**105**	-12.2611	-9.3337	-34.3203	-8.9552	-22.6323	-12.2611
**105**	-11.9169	-29.0456	-43.3516	-8.5363	-42.9096	-11.9169
**105**	-11.9161	-19.0928	-41.7480	-11.8161	-32.5323	-11.9161
**105**	-11.8276	0.4808	-38.1390	-12.8112	-1.2999	-11.8276
**105**	-11.6509	-19.1721	-42.9286	-13.0212	-15.4250	-11.6509
**105**	-11.5686	-4.1587	-31.7187	-8.0392	-26.6310	-11.5686
**105**	-11.5016	-5.3967	-45.9726	-8.7194	5.8071	-11.5016
**106**	-12.8380	-31.3624	-39.3547	-8.3808	-36.8283	-12.8380
**106**	-12.6549	-32.0140	-43.9789	-8.6597	-34.1170	-12.6549
**106**	-12.0849	-35.5942	-36.1921	-9.2492	-31.7051	-12.0849
**106**	-11.8003	-32.0328	-31.7923	-8.9200	-37.4884	-11.8003
**106**	-11.5836	-25.4816	-42.5848	-8.6374	-34.3041	-11.5836
**106**	-10.8650	-24.8079	-34.0322	-8.5941	-3.0885	-10.8650
**106**	-10.7710	1.0294	-27.8828	-10.1598	-14.1422	-10.7710
**106**	-10.7505	-15.8679	-46.1101	-12.4416	-24.9690	-10.7505
**106**	-10.5953	-27.4131	-39.3755	-11.4717	-12.4948	-10.5953
**106**	-10.4992	-12.8825	-37.0383	-7.9968	-15.4262	-10.4992
**107**	-13.6560	-19.8593	-39.0524	-8.5377	-34.9460	-13.6560
**107**	-13.3772	-11.7068	-36.4815	-9.6916	-36.2841	-13.3772
**107**	-13.0635	-16.9323	-35.6122	-7.2690	-43.3134	-13.0635
**107**	-12.7387	-3.1966	-44.0839	-10.4600	-21.2817	-12.7387
**107**	-12.4258	-17.2141	-39.7807	-6.3906	-43.2329	-12.4258
**107**	-12.3761	5.3447	-38.1959	-7.9616	-19.7997	-12.3761
**107**	-11.8839	-8.8793	-30.7997	-6.3548	-33.6431	-11.8839
**107**	-11.8830	-20.4228	-48.6990	-9.8811	-39.5900	-11.8830
**107**	-11.7609	19.4223	-32.0701	-7.3757	-35.5953	-11.7609

**107**	-11.2813	5.4342	-38.0325	-8.4482	13.9282	-11.2813
**108**	-14.2532	-6.5554	-32.7844	-7.7837	-39.5474	-14.2532
**108**	-13.2128	-2.3095	-38.0058	-6.9601	-35.0498	-13.2128
**108**	-13.1052	-18.0063	-41.1221	-8.8349	-54.4797	-13.1052
**108**	-13.1012	-14.8590	-40.1797	-5.8418	-45.1053	-13.1012
**108**	-12.8433	-19.2143	-36.5176	-6.6693	-45.3297	-12.8433
**108**	-12.8241	-11.5783	-51.4954	-11.5156	-48.2989	-12.8241
**108**	-12.5439	-17.6870	-31.0016	-7.1412	-32.5006	-12.5439
**108**	-12.4551	9.6269	-44.3344	-8.7019	-21.2275	-12.4551
**108**	-11.9461	-8.4717	-28.0856	-7.4854	-51.6835	-11.9461
**108**	-11.7638	37.1281	-21.3482	-7.0689	15.1441	-11.7638
**109**	-13.6486	-15.8015	-38.3429	-9.7004	-34.1549	-13.6486
**109**	-13.1803	-19.3264	-38.0090	-9.6534	-47.5871	-13.1803
**109**	-12.4202	-16.3452	-37.0547	-10.1882	-32.0035	-12.4202
**109**	-12.3547	-17.4839	-50.4476	-12.6722	-46.0893	-12.3547
**109**	-12.3148	-16.4322	-37.3541	-9.2936	-47.6331	-12.3148
**109**	-12.1717	-8.2175	-50.9096	-14.4905	-35.8276	-12.1717
**109**	-12.1344	-16.2296	-34.7105	-8.7344	-25.2731	-12.1344
**109**	-12.0240	-15.7676	-42.4748	-11.6449	-25.7179	-12.0240
**109**	-11.9406	-8.4269	-33.4581	-8.8052	-44.3456	-11.9406
**109**	-11.9282	-9.9562	-32.8257	-9.6981	-39.7703	-11.9282
**110**	-13.6251	-16.5257	-36.8799	-7.2498	-49.8227	-13.6251
**110**	-13.3715	-14.1777	-39.9236	-12.9280	-42.3020	-13.3715
**110**	-13.3419	-21.0900	-33.0154	-10.6558	-46.0461	-13.3419
**110**	-13.3095	-20.5705	-30.2201	-7.0429	-45.6255	-13.3095
**110**	-13.1535	-5.9211	-39.0711	-6.8718	-43.0037	-13.1535
**110**	-13.0845	-19.8865	-41.2342	-8.1642	-36.3238	-13.0845
**110**	-13.0420	-10.1343	-40.8746	-7.9904	-24.1019	-13.0420
**110**	-12.8191	-18.8791	-43.5433	-10.3859	-34.9438	-12.8191
**110**	-12.5993	-5.3241	-25.3167	-9.5287	-28.4308	-12.5993
**110**	-12.3990	-18.5326	-26.1022	-9.0165	-32.8311	-12.3990
**111**	-13.8409	-43.1323	-46.8640	-15.1132	-32.6597	-13.8409
**111**	-12.9402	-45.9776	-42.0135	-9.7253	-32.8162	-12.9402
**111**	-12.7890	-45.1864	-39.3825	-8.4628	-50.8492	-12.7890
**111**	-12.7423	-48.2903	-38.4855	-7.9859	-51.1652	-12.7423
**111**	-12.0338	-36.0260	-26.8280	-8.1676	-48.6688	-12.0338
**111**	-11.7969	-37.9090	-31.3148	-11.8390	-36.4672	-11.7969
**111**	-11.6041	-26.7128	-32.9927	-8.8547	-29.5818	-11.6041
**111**	-11.4976	-42.4848	-50.0180	-12.4750	-33.0035	-11.4976
**111**	-11.2706	-35.8504	-49.9056	-12.2222	-37.2042	-11.2706
**111**	-10.8648	-42.8687	-35.6526	-8.2007	-41.5713	-10.8648

Table S7. Ten top scored docking positioning of the SIRT2 inhibitors **112-116** at the 4RMG PDB code (MOE software). The predicted ΔG value of each protein-ligand complex has been reported, as calculated in terms of final scoring function (S, as Kcal/mol).

Compound	S	E_conf	E_place	E_score1	E_refine	E_score2
**112**	-5.3404	59.8378	-16.3372	-8.6437	-26.4935	-5.3404
**112**	-5.3265	61.6343	-18.8272	-8.2548	-28.2900	-5.3265
**112**	-5.3083	60.0833	-20.3968	-8.6529	-29.6896	-5.3083
**112**	-5.2745	63.1445	-21.8302	-8.2616	-27.0410	-5.2745
**112**	-5.2461	61.5283	-23.3147	-8.4870	-28.8639	-5.2461
**112**	-5.2074	62.8579	-20.4852	-8.3638	-28.0144	-5.2074
**112**	-5.1784	56.8209	-18.8881	-8.7044	-25.9658	-5.1784
**112**	-5.0579	57.1992	-18.6478	-8.3481	-26.9594	-5.0579
**112**	-4.9180	61.9024	-19.2827	-8.4992	-29.0794	-4.9180
**112**	-4.7806	60.1270	-19.1407	-8.4167	-24.3645	-4.7806
**113**	-6.3165	44.2262	-15.2498	-8.5265	-31.4510	-6.3165
**113**	-5.4965	38.3458	-16.9127	-7.9596	-28.8435	-5.4965
**113**	-5.2981	39.7475	-16.5890	-8.1990	-27.5821	-5.2981
**113**	-5.1898	42.3413	-15.6630	-8.0201	-25.2773	-5.1898
**113**	-5.1765	40.3803	-16.6091	-8.3596	-28.0338	-5.1765
**113**	-5.1283	36.8748	-18.1882	-8.3079	-26.4272	-5.1283
**113**	-5.1130	39.1993	-18.4080	-8.6236	-29.9559	-5.1130
**113**	-5.1074	40.9832	-19.3570	-8.2924	-28.2961	-5.1074
**113**	-5.0882	39.4675	-17.5327	-8.1827	-27.2754	-5.0882
**113**	-4.8356	39.0311	-17.2206	-7.9864	-28.2777	-4.8356
**114**	-7.6047	56.1428	-19.8853	-8.4233	-32.8524	-7.6047
**114**	-7.5091	53.3907	-24.5982	-8.2436	-35.7618	-7.5091
**114**	-7.2908	48.8385	-19.2412	-8.2456	-33.5995	-7.2908
**114**	-7.2465	60.1492	-19.9816	-8.9000	-35.4482	-7.2465
**114**	-7.0725	54.4772	-22.4791	-9.8409	-32.5215	-7.0725
**114**	-7.0702	52.3370	-26.0485	-9.5573	-28.7765	-7.0702
**114**	-7.0329	58.9761	-24.8942	-8.3054	-1.6790	-7.0329
**114**	-6.7586	52.4518	-29.2516	-8.3379	-27.2222	-6.7586
**114**	-6.7520	49.1823	-17.1326	-8.4774	-25.2842	-6.7520
**114**	-6.7231	50.2336	-24.7436	-8.4636	-32.5501	-6.7231
**115**	-8.1909	56.1645	-24.7630	-8.9699	-32.9392	-8.1909
**115**	-8.0161	47.2657	-24.2299	-9.4777	-36.1067	-8.0161
**115**	-7.9050	49.7090	-20.4748	-10.7569	-34.0771	-7.9050
**115**	-7.8454	46.6672	-21.5455	-8.8880	-32.5392	-7.8454
**115**	-7.7931	42.1018	-22.5821	-8.7167	-34.2423	-7.7931
**115**	-7.6376	45.2911	-28.3659	-9.0910	-34.4934	-7.6376
**115**	-7.5658	43.7207	-26.5945	-9.5005	-35.2860	-7.5658
**115**	-7.3632	48.1657	-22.4882	-8.8172	-30.9688	-7.3632
**115**	-7.3009	43.1822	-27.0364	-10.0190	-30.9944	-7.3009
**115**	-7.2491	48.6016	-22.4063	-9.5644	-25.4465	-7.2491
**116**	-7.2539	46.7363	-19.6167	-9.5343	-36.4286	-7.2539
**116**	-7.1088	42.0126	-29.5901	-9.4017	-32.8432	-7.1088
**116**	-6.7788	43.3001	-17.9842	-9.1838	-25.6907	-6.7788
**116**	-6.7039	37.7617	-20.8041	-9.9379	-33.4346	-6.7039
**116**	-6.6723	41.8600	-18.2389	-9.2197	-31.6015	-6.6723

**116**	-6.6704	40.0015	-23.4932	-10.2214	-35.0815	-6.6704
**116**	-6.6428	37.6876	-21.8879	-9.4988	-35.0622	-6.6428
**116**	-6.4847	40.2980	-24.3624	-10.4979	-28.6348	-6.4847
**116**	-6.4783	39.2429	-26.3302	-9.7845	-33.8369	-6.4783
**116**	-6.4019	41.5733	-23.6571	-9.9247	-29.7559	-6.4019

Table S8. Ten top scored docking positioning of the SIRT2 inhibitors **112-116** at the 5MAT PDB code (MOE software). The predicted ΔG value of each protein-ligand complex has been reported, as calculated in terms of final scoring function (S, as Kcal/mol).

Compound	S	E_conf	E_place	E_score1	E_refine	E_score2
**112**	-5.4023	58.0304	-24.6803	-9.0243	-31.3765	-5.4023
**112**	-5.3284	58.0911	-29.9347	-8.9925	-28.4771	-5.3284
**112**	-5.2932	57.3992	-21.7561	-9.7625	-30.7691	-5.2932
**112**	-5.0898	60.5048	-21.2533	-9.1751	-20.4286	-5.0898
**112**	-5.0898	56.9218	-24.3986	-10.3952	-22.9202	-5.0898
**112**	-5.0698	58.6302	-21.6301	-9.1057	-27.5706	-5.0698
**112**	-4.9684	58.6663	-26.4878	-9.4748	-27.9082	-4.9684
**112**	-4.7547	61.2353	-21.2899	-8.9922	-23.9356	-4.7547
**112**	-4.7182	57.3022	-24.1629	-10.3518	-23.2956	-4.7182
**112**	-4.7069	57.8997	-23.2594	-9.5909	-24.2361	-4.7069
**113**	-5.9011	42.4399	-21.5776	-9.2598	-28.3076	-5.9011
**113**	-5.6987	39.7731	-20.0498	-9.5829	-25.0967	-5.6987
**113**	-5.5278	44.2905	-19.7953	-9.1534	-32.5271	-5.5278
**113**	-5.3895	37.7410	-20.5915	-9.9990	-24.5152	-5.3895
**113**	-5.3248	36.2321	-27.8934	-9.1819	-31.0722	-5.3248
**113**	-5.3060	39.8297	-18.1193	-10.0368	-14.2443	-5.3060
**113**	-5.1056	36.2650	-28.8735	-9.1303	-30.1118	-5.1056
**113**	-5.0768	35.2090	-25.7017	-9.7220	-24.0103	-5.0768
**113**	-5.0683	37.6896	-20.6784	-10.5521	-24.9659	-5.0683
**113**	-5.0535	37.3638	-18.6594	-9.2848	-30.8609	-5.0535
**114**	-7.5841	54.1520	-35.8740	-10.0868	-36.3495	-7.5841
**114**	-7.4988	49.1339	-23.6264	-10.5368	-34.0066	-7.4988
**114**	-7.2571	56.0720	-27.0842	-9.4126	-26.3210	-7.2571
**114**	-6.9616	49.7687	-27.4455	-10.4168	-22.8566	-6.9616
**114**	-6.9084	48.9685	-25.7429	-9.7629	-26.6270	-6.9084
**114**	-6.8979	49.6057	-28.9360	-13.0813	-28.1924	-6.8979
**114**	-6.8454	49.5683	-29.4569	-9.7046	-36.0863	-6.8454
**114**	-6.8243	50.9265	-31.4947	-9.6330	-34.2884	-6.8243
**114**	-6.7689	52.3632	-31.4140	-9.4984	-31.5505	-6.7689
**114**	-6.7566	58.4894	-25.4328	-9.3952	-21.7167	-6.7566
**115**	-8.9498	50.2785	-34.2241	-9.7881	-24.0972	-8.9498
**115**	-8.7633	43.5833	-34.4952	-10.8327	-26.8257	-8.7633
**115**	-8.4265	56.7462	-25.5619	-10.7349	-9.5371	-8.4265

**115**	-7.9018	48.0603	-34.2411	-9.4160	-28.8188	-7.9018
**115**	-7.7322	60.0283	-29.8330	-10.0257	-13.0355	-7.7322
**115**	-7.7089	44.9534	-35.7338	-9.3509	-31.4795	-7.7089
**115**	-7.6974	47.9193	-38.3521	-9.2723	-36.8062	-7.6974
**115**	-7.3669	43.7385	-23.4590	-9.8761	-29.2321	-7.3669
**115**	-7.2136	45.6139	-28.2125	-9.5690	-28.4975	-7.2136
**115**	-7.1958	44.1212	-40.2594	-9.4507	-37.3636	-7.1958
**116**	-7.8486	44.7246	-30.2459	-11.5293	-23.6337	-7.8486
**116**	-7.8246	40.9332	-33.7298	-11.7900	-27.2326	-7.8246
**116**	-7.5013	50.5289	-28.8678	-10.3889	-8.3230	-7.5013
**116**	-7.3001	40.2446	-28.6276	-10.7942	-26.2434	-7.3001
**116**	-7.0524	39.7383	-32.6974	-10.2507	-22.8952	-7.0524
**116**	-7.0260	43.3439	-33.3518	-10.2843	-29.9638	-7.0260
**116**	-6.9430	43.6470	-30.9061	-10.1495	-38.6868	-6.9430
**116**	-6.8599	44.5372	-28.3784	-11.2958	-30.0644	-6.8599
**116**	-6.7727	39.4847	-27.2874	-10.8823	-24.1711	-6.7727
**116**	-6.6726	46.7541	-28.0513	-10.6129	-23.5490	-6.6726

Table S9. Rigid protein Virtual screening. Ten top scored docking positioning of the novel identified SIRT2 inhibitors **1a**-**7a** at the 4RMG PDB code (MOE software). The SIRT2 inhibitor AGK2 has been also considered as reference compound. The predicted ΔG value of each protein-ligand complex has been reported, as calculated in terms of final scoring function (S, as Kcal/mol).

**Compound**	**S**	**E_conf**	**E_place**	**E_score1**	**E_refine**	**E_score2**
**1a**	-9.4004	34.2832	-26.4484	-8.8771	-7.4778	-9.4004
**1a**	-9.0980	39.6262	-23.6360	-10.5270	-14.8229	-9.0980
**1a**	-8.9235	37.4397	-19.8001	-8.5350	-16.4555	-8.9235
**1a**	-8.7738	46.7376	-25.1603	-8.8724	0.3711	-8.7738
**1a**	-8.2850	38.8720	-20.8246	-8.6022	-21.8182	-8.2850
**1a**	-8.0325	28.0057	-21.1364	-9.2539	-25.5420	-8.0325
**1a**	-8.0218	26.5764	-26.4965	-9.4738	-26.9227	-8.0218
**1a**	-7.9091	28.2035	-19.0495	-8.5543	-26.2193	-7.9091
**1a**	-7.8140	27.2928	-23.5073	-8.8179	-19.9696	-7.8140
**1a**	-7.7192	44.5577	-26.3606	-8.3942	-15.0075	-7.7192
**2a**	-10.0158	33.7044	-28.5256	-8.9755	-4.1568	-10.0158
**2a**	-10.0043	47.1255	-26.2476	-8.9433	2.5064	-10.0043
**2a**	-9.9162	69.4167	-17.9713	-8.7402	23.6811	-9.9162
**2a**	-9.7029	31.5735	-21.8140	-8.1540	-15.1579	-9.7029
**2a**	-9.2695	31.8094	-23.5908	-8.3551	-3.6524	-9.2695
**2a**	-8.9896	22.3920	-23.9999	-9.0055	-21.4666	-8.9896
**2a**	-8.8657	34.1510	-17.8360	-7.9587	-9.5086	-8.8657
**2a**	-8.7431	27.0064	-16.2796	-8.1977	-23.0232	-8.7431
**2a**	-8.6515	31.1323	-14.2086	-8.8127	-13.8846	-8.6515
**2a**	-8.5009	37.6623	-19.3668	-8.1114	-11.6729	-8.5009

**3a**	-9.4095	13.5647	-26.2863	-10.8934	-4.9516	-9.4095
**3a**	-9.3214	11.9038	-20.8187	-8.5315	-8.9750	-9.3214
**3a**	-9.2346	12.7771	-21.7444	-8.5093	-12.6848	-9.2346
**3a**	-9.1770	16.1019	-18.6855	-9.4505	-10.3090	-9.1770
**3a**	-9.0238	18.5116	-26.9030	-10.0900	0.3880	-9.0238
**3a**	-8.9911	16.6799	-19.2242	-8.7453	-22.7360	-8.9911
**3a**	-8.9487	20.5321	-17.3321	-8.6670	14.3788	-8.9487
**3a**	-8.8221	27.8127	-20.2365	-9.8340	16.7268	-8.8221
**3a**	-8.3943	22.0333	-21.6604	-8.8903	-6.6167	-8.3943
**3a**	-8.2425	7.9952	-23.9063	-8.6930	-25.1096	-8.2425
**4a**	-8.9714	1.8032	-20.7065	-7.9314	-7.7120	-8.9714
**4a**	-8.8710	11.6294	-21.3511	-9.6427	-11.4867	-8.8710
**4a**	-8.7651	11.6405	-29.4715	-9.8587	-15.2393	-8.7651
**4a**	-8.3924	48.8920	-15.6768	-8.5757	39.5095	-8.3924
**4a**	-8.2901	7.0567	-20.5867	-8.0299	-11.5678	-8.2901
**4a**	-8.1999	20.3162	-25.8684	-11.0583	53.5885	-8.1999
**4a**	-8.1857	0.5710	-22.8665	-9.3643	-26.9827	-8.1857
**4a**	-7.8969	12.1843	-26.1390	-9.0207	-13.4661	-7.8969
**4a**	-7.8297	17.5383	-28.9195	-8.2356	-9.4861	-7.8297
**4a**	-7.7045	27.0419	-13.6158	-8.4785	0.8708	-7.7045
**5a**	-9.0509	-5.3699	-19.7405	-9.5322	-14.4267	-9.0509
**5a**	-8.7092	-11.3884	-14.5392	-10.3442	-11.5082	-8.7092
**5a**	-8.5754	-3.5155	-22.0600	-8.7457	-4.9687	-8.5754
**5a**	-8.3696	-10.0206	-32.3261	-8.8055	-23.6363	-8.3696
**5a**	-8.2574	-4.8716	-25.9557	-10.7752	-9.6070	-8.2574
**5a**	-8.0120	-23.1228	-25.9350	-8.3756	-23.5931	-8.0120
**5a**	-7.8355	10.0989	-24.5592	-10.1955	17.2742	-7.8355
**5a**	-7.8120	2.1871	-24.3836	-8.5911	7.2494	-7.8120
**5a**	-7.7134	-11.9154	-19.3414	-10.2077	-17.1831	-7.7134
**5a**	-7.6351	30.3790	-22.1878	-8.4060	42.9440	-7.6351
**6a**	-10.2478	7.8728	-21.8975	-10.1859	3.6999	-10.2478
**6a**	-9.8349	7.8143	-30.6188	-9.4173	4.1768	-9.8349
**6a**	-9.8206	0.2044	-25.9691	-10.3372	-24.0262	-9.8206
**6a**	-9.8161	13.4661	-28.7141	-11.9312	-10.1151	-9.8161
**6a**	-9.7389	10.8004	-22.6697	-10.0088	-11.3049	-9.7389
**6a**	-9.7264	11.0218	-26.8641	-8.5777	-14.7350	-9.7264
**6a**	-9.5483	17.2348	-17.3659	-8.6805	7.1432	-9.5483
**6a**	-9.4567	8.2724	-26.6179	-9.3536	-19.4069	-9.4567
**6a**	-9.2866	-1.4136	-22.5072	-9.9414	-20.6150	-9.2866
**6a**	-9.1944	9.2750	-17.4803	-8.3166	-8.3928	-9.1944
**7a**	-10.7072	61.5467	-23.1041	-8.5819	-8.9134	-10.7072
**7a**	-10.6169	75.2277	-20.0870	-8.6678	5.8752	-10.6169
**7a**	-10.6089	58.8829	-20.4931	-9.5911	-4.9472	-10.6089
**7a**	-10.5852	43.2278	-22.9289	-8.6362	-3.6911	-10.5852
**7a**	-10.5111	55.2774	-18.9151	-9.5828	0.5999	-10.5111
**7a**	-10.4843	44.5222	-21.0537	-8.8491	-3.4183	-10.4843

**7a**	-10.3800	44.4986	-21.2328	-8.9472	-3.9157	-10.3800
**7a**	-10.3766	53.8000	-20.9551	-8.8092	8.2720	-10.3766
**7a**	-10.3617	58.3304	-27.6435	-9.3668	4.5437	-10.3617
**7a**	-10.1839	43.9191	-26.2669	-9.1892	-7.1624	-10.1839
**AGK2**	-12.3545	43.1796	-27.9120	-7.9005	-1.2242	-12.3545

Table S10. Rigid protein Virtual screening. Ten top scored docking positioning of the novel identified SIRT2 inhibitors **1a-7a** at the 5MAT PDB code (MOE software). The SIRT2 inhibitor AGK2 has been also considered as reference compound. The predicted ΔG value of each protein-ligand complex has been reported, as calculated in terms of final scoring function (S, as Kcal/mol).

Compound	S	E_conf	E_place	E_score1	E_refine	E_score2
**1a**	-8.5706	39.7190	-31.6267	-9.1109	-17.4603	-8.5706
**1a**	-8.2475	29.7915	-27.8109	-8.2177	-12.4591	-8.2475
**1a**	-8.0268	42.9923	-29.6301	-8.1718	-0.9737	-8.0268
**1a**	-7.9812	34.1350	-35.0882	-10.5088	4.1888	-7.9812
**1a**	-7.9694	29.8000	-26.6836	-8.2964	1.4244	-7.9694
**1a**	-7.7901	30.0656	-20.3196	-9.3502	-10.5859	-7.7901
**1a**	-7.6679	38.4283	-18.6014	-8.0613	-1.0470	-7.6679
**1a**	-7.6545	34.4443	-20.4333	-9.0135	-12.1696	-7.6545
**1a**	-7.6514	32.4146	-26.3872	-9.4187	5.6106	-7.6514
**1a**	-7.6252	35.6247	-28.3564	-12.4230	-5.9837	-7.6252
**2a**	-9.4364	35.2733	-35.3793	-8.6924	3.9134	-9.4364
**2a**	-9.4276	27.6578	-35.4135	-8.1682	-12.1070	-9.4276
**2a**	-9.2711	31.7483	-25.4143	-8.1453	-20.1741	-9.2711
**2a**	-9.2337	48.8123	-26.4006	-9.0498	11.0988	-9.2337
**2a**	-9.0424	36.7183	-36.0633	-10.4274	-5.8903	-9.0424
**2a**	-8.9952	46.9316	-35.4645	-9.2158	-2.0447	-8.9952
**2a**	-8.9628	43.4355	-26.3714	-8.1427	14.3257	-8.9628
**2a**	-8.9233	33.1059	-35.0816	-9.2701	1.3757	-8.9233
**2a**	-8.6349	42.3913	-32.4158	-8.5784	-4.2029	-8.6349
**2a**	-8.5070	54.7463	-33.5414	-8.4096	32.2391	-8.5070
**3a**	-8.8986	14.2885	-21.5164	-9.8610	4.9477	-8.8986
**3a**	-8.8334	17.8821	-29.8018	-9.9412	29.0717	-8.8334
**3a**	-8.4582	23.4765	-33.4347	-9.2954	15.3096	-8.4582
**3a**	-8.4518	9.6196	-29.1194	-9.4483	-6.3696	-8.4518
**3a**	-8.3834	13.0392	-30.4483	-9.8047	-3.0555	-8.3834
**3a**	-8.3659	15.7303	-34.8093	-9.8573	-3.2631	-8.3659
**3a**	-8.3309	14.0364	-32.2264	-9.7231	-14.8324	-8.3309
**3a**	-8.3302	18.8577	-32.4724	-10.4734	-18.3485	-8.3302
**3a**	-8.0978	19.3630	-35.6233	-9.8700	-17.9022	-8.0978
**3a**	-7.9813	20.6845	-36.2188	-9.7647	-7.8962	-7.9813
**4a**	-9.7765	8.9145	-28.6109	-10.2765	-18.7757	-9.7765
**4a**	-9.7092	13.1769	-33.3017	-9.6286	-12.7891	-9.7092
**4a**	-9.4539	17.1454	-36.0267	-11.4404	27.1018	-9.4539
**4a**	-9.1791	4.3688	-31.1896	-10.4415	-16.5454	-9.1791

**4a**	-9.1107	11.1215	-40.8968	-9.9579	-17.2215	-9.1107
**4a**	-9.0711	12.7261	-30.8175	-10.3442	-14.2492	-9.0711
**4a**	-8.9619	21.3356	-29.3996	-9.7596	17.6367	-8.9619
**4a**	-8.9590	5.4430	-33.1400	-10.6194	-3.1835	-8.9590
**4a**	-8.7561	5.7262	-28.7116	-10.8634	-14.9002	-8.7561
**4a**	-8.5978	5.0440	-25.7699	-9.8994	-8.2443	-8.5978
**5a**	-9.9680	-13.4394	-26.9203	-9.0680	-20.1495	-9.9680
**5a**	-9.8936	-9.2862	-34.5184	-8.7033	-10.2397	-9.8936
**5a**	-9.4852	-11.6869	-33.0015	-10.6444	-19.3875	-9.4852
**5a**	-9.3342	-11.0063	-25.1340	-9.4906	-16.7457	-9.3342
**5a**	-9.2818	-6.4384	-30.3503	-8.8831	-12.4089	-9.2818
**5a**	-9.2224	-10.6289	-27.7229	-11.8456	-10.7057	-9.2224
**5a**	-9.0125	-8.9704	-27.9633	-9.2092	1.7897	-9.0125
**5a**	-8.9629	-3.1532	-29.7299	-9.6717	17.6341	-8.9629
**5a**	-8.8553	-17.9331	-24.4935	-10.0497	-13.4442	-8.8553
**5a**	-8.5990	17.7923	-27.9206	-11.3114	66.8918	-8.5990
**6a**	-10.9884	10.8527	-39.5053	-10.7373	-16.0098	-10.9884
**6a**	-10.8360	3.7936	-30.9982	-10.7509	-14.6279	-10.8360
**6a**	-10.7763	24.2042	-32.5021	-9.7274	27.5252	-10.7763
**6a**	-10.6794	8.1983	-27.2891	-10.0424	-5.1963	-10.6794
**6a**	-10.5061	10.6062	-29.3086	-11.2600	-11.8971	-10.5061
**6a**	-10.4118	13.6811	-35.3761	-11.0359	-11.4316	-10.4118
**6a**	-9.7078	9.6792	-30.7673	-9.6506	2.2642	-9.7078
**6a**	-9.5490	19.0121	-33.4839	-9.7753	44.5090	-9.5490
**6a**	-9.5220	28.8933	-35.4535	-10.1640	6.9597	-9.5220
**6a**	-9.2936	23.3174	-25.7204	-9.6846	12.8830	-9.2936
**7a**	-9.9227	54.2778	-34.6917	-10.7258	3.1148	-9.9227
**7a**	-9.5259	55.9304	-25.8540	-8.9765	11.5004	-9.5259
**7a**	-9.4850	53.3920	-37.1103	-8.7369	4.6319	-9.4850
**7a**	-9.4821	66.4844	-29.7207	-9.1317	25.4970	-9.4821
**7a**	-9.3514	71.9428	-31.3255	-8.4030	2.5715	-9.3514
**7a**	-9.1916	54.6667	-27.3826	-9.1572	12.3761	-9.1916
**7a**	-9.1491	64.0590	-32.5278	-8.3140	1.9188	-9.1491
**7a**	-9.1452	69.7133	-27.9359	-10.4305	61.2354	-9.1452
**7a**	-9.0220	72.5005	-33.1113	-8.4135	2.6576	-9.0220
**7a**	-8.6434	67.3448	-22.7825	-8.3873	27.7506	-8.6434
**AGK2**	-11.7728	62.7179	-28.1687	-7.6774	40.1252	-11.7728

Table S11. Flexible protein Virtual screening. Five top scored docking positioning of the novel identified SIRT2 inhibitors **1a**-**7a** at the 4RMG PDB code (MOE software). The SIRT2 inhibitor AGK2 has been also considered as reference compound. The predicted ΔG value of each protein-ligand complex has been reported, as calculated in terms of final scoring function (S, as Kcal/mol).

Compound	S	E_conf	E_place	E_score1	E_refine	E_score2
**1a**	-9.5617	30.1341	-24.8953	-10.5752	-19.1446	-9.5617
**1a**	-9.5488	29.3902	-27.6763	-9.1281	-19.0177	-9.5488
**1a**	-9.4884	30.6250	-23.8048	-8.3081	-19.0965	-9.4884
**1a**	-8.6303	35.2950	-27.9394	-8.3756	-18.3631	-8.6303
**1a**	-8.4510	36.3321	-26.9235	-8.5330	-23.6778	-8.4510
**2a**	-10.5439	25.8577	-24.2694	-8.2227	-3.8073	-10.5439
**2a**	-10.3583	38.9237	-25.5446	-8.0893	-2.8473	-10.3583
**2a**	-10.3562	38.3786	-28.0415	-8.5344	-17.9208	-10.3562
**2a**	-10.3326	44.1909	-18.3727	-8.3089	-0.7735	-10.3326
**2a**	-10.1423	31.8108	-28.2591	-10.5753	15.9216	-10.1423
**3a**	-9.5571	8.7658	-26.6166	-9.8003	-13.9148	-9.5571
**3a**	-9.4864	14.3085	-26.5698	-9.8797	-13.9755	-9.4864
**3a**	-9.4290	8.0651	-22.8752	-8.8471	-19.1138	-9.4290
**3a**	-9.2953	17.4309	-25.9498	-8.8664	-21.3621	-9.2953
**3a**	-9.2666	13.5796	-16.8761	-8.8021	-24.3819	-9.2666
**4a**	-9.4795	12.7061	-25.8140	-9.0960	-13.9820	-9.4795
**4a**	-9.3082	9.7623	-21.8115	-8.9848	-13.0469	-9.3082
**4a**	-9.2204	9.5686	-13.0012	-10.4389	-5.5669	-9.2204
**4a**	-9.0953	19.4831	-24.8256	-8.7022	-18.3639	-9.0953
**4a**	-8.9591	8.0038	-24.6987	-8.6367	-12.7982	-8.9591
**5a**	-9.8703	-14.6966	-24.8607	-9.8611	-1.1262	-9.8703
**5a**	-9.1115	-9.5073	-24.4054	-10.5811	-20.8337	-9.1115
**5a**	-8.9825	-11.2181	-18.8741	-9.7312	-21.6268	-8.9825
**5a**	-8.4246	-20.7734	-24.6435	-8.7084	-7.7907	-8.4246
**5a**	-8.2710	-8.8776	-26.1773	-9.3543	-13.5277	-8.2710
**6a**	-10.3828	13.7101	-26.5277	-9.7503	-20.0673	-10.3828
**6a**	-10.3184	15.6072	-28.9513	-10.3406	-3.2860	-10.3184
**6a**	-10.2685	11.6418	-20.3297	-8.6215	-22.0868	-10.2685
**6a**	-10.0371	10.4418	-30.7654	-9.9595	-23.8399	-10.0371
**6a**	-9.8793	8.3028	-27.7035	-8.8663	-19.4066	-9.8793
**7a**	-10.9785	56.5435	-17.8592	-8.8760	-8.7181	-10.9785
**7a**	-10.6662	57.6456	-22.3537	-10.9067	-20.4469	-10.6662
**7a**	-10.6378	52.9520	-31.1625	-10.4170	-17.9076	-10.6378
**7a**	-10.5375	47.8347	-20.8423	-9.3579	-11.9879	-10.5375
**7a**	-10.2985	53.1118	-26.2195	-10.1949	-6.5685	-10.2985
**AGK2**	-11,5559	43.7486	-25.3648	-8.7591	12.0598	-11.5559

Table S12. Flexible protein Virtual screening. Five top scored docking positioning of the novel identified SIRT2 inhibitors **1a**-**7a** at the 5MAT PDB code (MOE software). The SIRT2 inhibitor AGK2 has been also considered as reference compound. The predicted ΔG value of each protein-ligand complex has been reported, as calculated in terms of final scoring function (S, as Kcal/mol).

mseq	S	E_conf	E_place	E_score1	E_refine	E_score2
**1a**	-8.5504	35.8090	-27.4786	-9.3227	-23.9326	-8.5504
**1a**	-8.0335	41.8086	-25.0820	-9.1672	10.4411	-8.0335
**1a**	-8.0008	38.8853	-35.6432	-10.6364	-16.4839	-8.0008
**1a**	-7.4592	33.5926	-25.0119	-9.2003	-23.2686	-7.4592
**1a**	-7.4078	26.0521	-28.1108	-11.1153	-15.4990	-7.4078
**2a**	-9.2688	34.1839	-31.3363	-9.5055	-13.4631	-9.2688
**2a**	-9.2660	33.1133	-37.1513	-9.7663	-17.8508	-9.2660
**2a**	-9.1151	44.0130	-37.4914	-9.8515	-12.3586	-9.1151
**2a**	-9.0434	30.5912	-36.5789	-11.0853	-28.2654	-9.0434
**2a**	-8.9878	25.2055	-33.9332	-10.2823	-12.3852	-8.9878
**3a**	-8.7362	9.0098	-29.7506	-9.6353	-20.9744	-8.7362
**3a**	-8.5721	11.6969	-32.7220	-10.0383	-21.4420	-8.5721
**3a**	-8.3286	18.8645	-34.1676	-10.0219	-7.1967	-8.3286
**3a**	-8.2745	16.7642	-21.2420	-10.0014	10.4518	-8.2745
**3a**	-7.9579	15.2782	-30.4212	-9.3204	-23.0374	-7.9579
**4a**	-11.4785	11.4543	-34.4188	-11.2323	-17.4558	-11.4785
**4a**	-9.2090	6.0519	-44.9981	-12.8925	-25.0428	-9.2090
**4a**	-9.1721	2.5929	-28.8207	-10.7807	-17.6604	-9.1721
**4a**	-8.9668	6.5661	-26.4576	-10.5660	-19.8077	-8.9668
**4a**	-8.6980	4.8363	-36.4894	-10.3089	-23.3939	-8.6980
**5a**	-9.3488	-16.3453	-36.5596	-10.9406	-26.6234	-9.3488
**5a**	-8.3594	-9.6579	-30.5816	-10.6039	-14.0273	-8.3594
**5a**	-8.3582	-17.5326	-31.7400	-12.9923	-23.3486	-8.3582
**5a**	-7.7948	-18.6275	-21.7908	-10.0312	-14.3554	-7.7948
**5a**	-7.7711	-19.1781	-25.8513	-10.2661	-16.3643	-7.7711
**6a**	-10.3401	6.2178	-40.5129	-10.4845	-21.7718	-10.3401
**6a**	-9.4694	9.7539	-29.7817	-11.2655	-8.4563	-9.4694
**6a**	-9.2092	10.4676	-30.5862	-10.1482	2.5850	-9.2092
**6a**	-9.0809	3.4790	-25.3108	-9.6256	-16.1220	-9.0809
**6a**	-8.8716	3.3537	-34.1313	-11.6179	-14.0789	-8.8716
**7a**	-10.3930	71.2262	-34.2017	-10.5311	14.9904	-10.3930
**7a**	-10.0579	47.7010	-35.7014	-10.1342	-17.5108	-10.0579
**7a**	-9.9081	50.9382	-35.1110	-9.0981	-8.4001	-9.9081
**7a**	-9.8345	70.1906	-30.3143	-9.7857	12.3281	-9.8345
**7a**	-9.8066	49.0991	-31.0803	-9.2944	-12.4422	-9.8066
**AGK2**	-11.3868	38.5745	-23.2340	-8.2233	-3.2912	-11.3868

Table S13. Calculated propertied based on the Lipinski's and Veber's rules as referred to the novel SIRT2 inhibitors **1a-7a** and to the reference compounds AGK2, SirReal2. Reliability index values for a number of descriptors are shown as R.I. (values higher than 0.30 are ranked as reliable by the software).

Comp.	MW[a]	N. H-bond acceptor[b]	N. H-bond donor[c]	N. rotable bonds[d]	cLogP GALAS[e] (R.I. ≥ 0.46)	TPSA[f]	HIA (%)[g]	Vd (l/kg)[h]	%PPB[i] (R.I. ≥ 0.34)	LogKa[l HAS] (R.I. ≥ 0.34)	%F (oral)[m] 50mg
**1a**	355.50	3	1	4	4.99	119.59	100	4.3	99.59	4.91	83.9
**2a**	360.93	2	1	5	5.88	78.65	100	4.8	99.69	5.07	64.3
**3a**	332.87	2	1	4	5.40	78.46	100	3.8	99.69	5.05	67.1
**4a**	353.46	4	2	4	4.06	107.56	100	2.3	99.13	4.42	96.5
**5a**	371.50	4	2	4	4.17	132.86	100	0.69	99.55	4.60	90.0
**6a**	407.43	4	2	5	4.32	107.65	100	2.7	99.71	4.77	81.4
**7a (YM08)**	367.49	4	0	2	3.14	87.04	100	2.2	98.07	4.73	98.5
**AGK2**	434.27	5	1	5	4.85	78.92	100	3.1	99.93	5.15	13.9
**SirReal2**	420.55	5	1	6	4.99	121.31	100	2.5	98.71	4.69	21.9

a Molecular weight; b Number of H-bond acceptors; c Number of H-bond donors; d Number of rotatable bonds; e Logarithmic ratio of the octanol–water partitioning coefficient; f Topological polar surface area; g HIA represents the human intestinal absorption, expressed as percentage of the molecule able to pass through the intestinal membrane; h prediction of Volume of Distribution (Vd) of the compound in the body; i percentage of plasmatic protein bound drug; l Ligand affinity toward human serum albumin (HSA); m Percentage oral bioavailability.

Table S14. Calculated ADMET descriptors concerning the novel SIRT2 inhibitors **1a**-**7a** and the reference compounds AGK2, SirReal2 (n.d.; not determined). Reliability index values for a number of descriptors are shown as R.I. (values higher than 0.30 are ranked as reliable by the software).

**Comp.**	**CYP3A4**[a]		**LD_{50} (mg/kg)**[b] Mouse oral (R.I. ≥ 0.3)	**PAINS** (Pan Assay Interference structures)
	**Inhibitor** **(IC_{50} < 10mM)** (R.I. ≥ 0.3)	**Substrate** (R.I. ≥ 0.3)		
**1a**	0.06	0.84	800	0 ALERT
**2a**	0.08	0.86	730	0 ALERT
**3a**	0.08	0.87	n.d.	0 ALERT
**4a**	0.07	0.88	n.d.	0 ALERT
**5a**	0.03	0.87	n.d.	0 ALERT
**6a**	0.10	0.90	n.d.	0 ALERT
**7a (YM-08)**	0.16	0.93	360	0 ALERT
**AGK2**	0.45	0.95	900	0 ALERT
**SirReal2**	0.89	0.97	1100	0 ALERT

a Prediction of the ligand inhibitor/substrate behavior towards cytochrome CYP3A4; b Acute toxicity (LD_{50}) for mouse after oral administration
